# Catalyst-Controlled Selectivity Switch in Three-Component Reaction: An NHC-Catalyzed Strategy for the Synthesis of δ-Lactone-Fused Spirobenzofuran-3-ones

**DOI:** 10.3390/molecules27185952

**Published:** 2022-09-13

**Authors:** Zhanyong Wang, Ting Yang, Dongfang Liu, Rongxiang Chen, Nan Wang, Hong Liu, Jiarong Li, Kaikai Wang, Hongxin Liu

**Affiliations:** 1 School of Pharmacy, Xinxiang University, Xinxiang 453003, China; 2 Nursing College, Xinxiang University, Xinxiang 453003, China; 3 Xinxiang Runyu Material Co., Ltd., Xinxiang 453003, China; 4 College of Chemistry and Materials Engineering, Wenzhou University, Wenzhou 325035, China; 5 Institute of New Materials & Industrial Technology, Wenzhou University, Wenzhou 325035, China

**Keywords:** N-heterocyclic carbenes, α,β-unsaturated acylazoliums, Spirobenzofuran-3-one, three-component reaction, δ-Lactones

## Abstract

An efficient, three-component reaction of aldehydes and benzofuran-3-ones was developed. This process provides a new approach for the preparation of synthetically and biologically important spirobenzofuran-3-one derivatives with moderate-to-good yields under mild conditions. A switch of intramolecular to intermolecular domino Michael–aldol–lactonization leading to differential product formation was achieved by different NHCs catalysis.

## 1. Introduction

**Figure 1:**
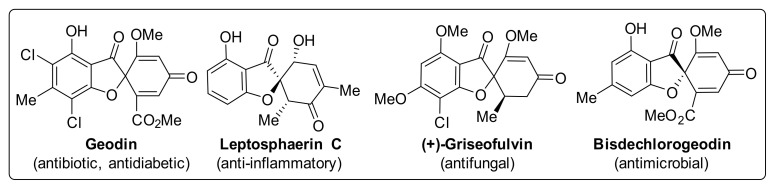
Naturally occurring bioactive products with spirobenzofuran-3-one core.

Spirobenzofuran-3-ones are an important class of structural scaffolds and widely occur in various natural products, bioactive molecules and pharmaceuticals [1,2,3,4,5,6,7,8,9,10]. In particular, the spiro-bicyclic skeleton has attracted considerable attention due to its outstanding bioactivity that includes, for example, antibiotic, antidiabetic, anti-inflammatory, antifungal and antimicrobial activities (Figure 1) [11,12,13,14,15]. Due to its widespread biological activity and inherent structural importance, great efforts have been devoted to effectively access spirobenzofuran-3-one derivatives [16,17,18,19,20,21,22,23], and a handful of synthetic transformations for the construction of spiro-bicyclic benzofuran-3-ones have been developed [24,25,26,27]. However, most of these reported strategies suffer from many deficiencies including multistep procedures, the requirement of a prefunctionalized benzofuran ring, expensive catalysts and in some cases harsh reaction conditions. Further development of a mild and facile method for the formation of spirobenzofuran-3-one starting from readily available materials is still very much needed.

**Scheme 1:**
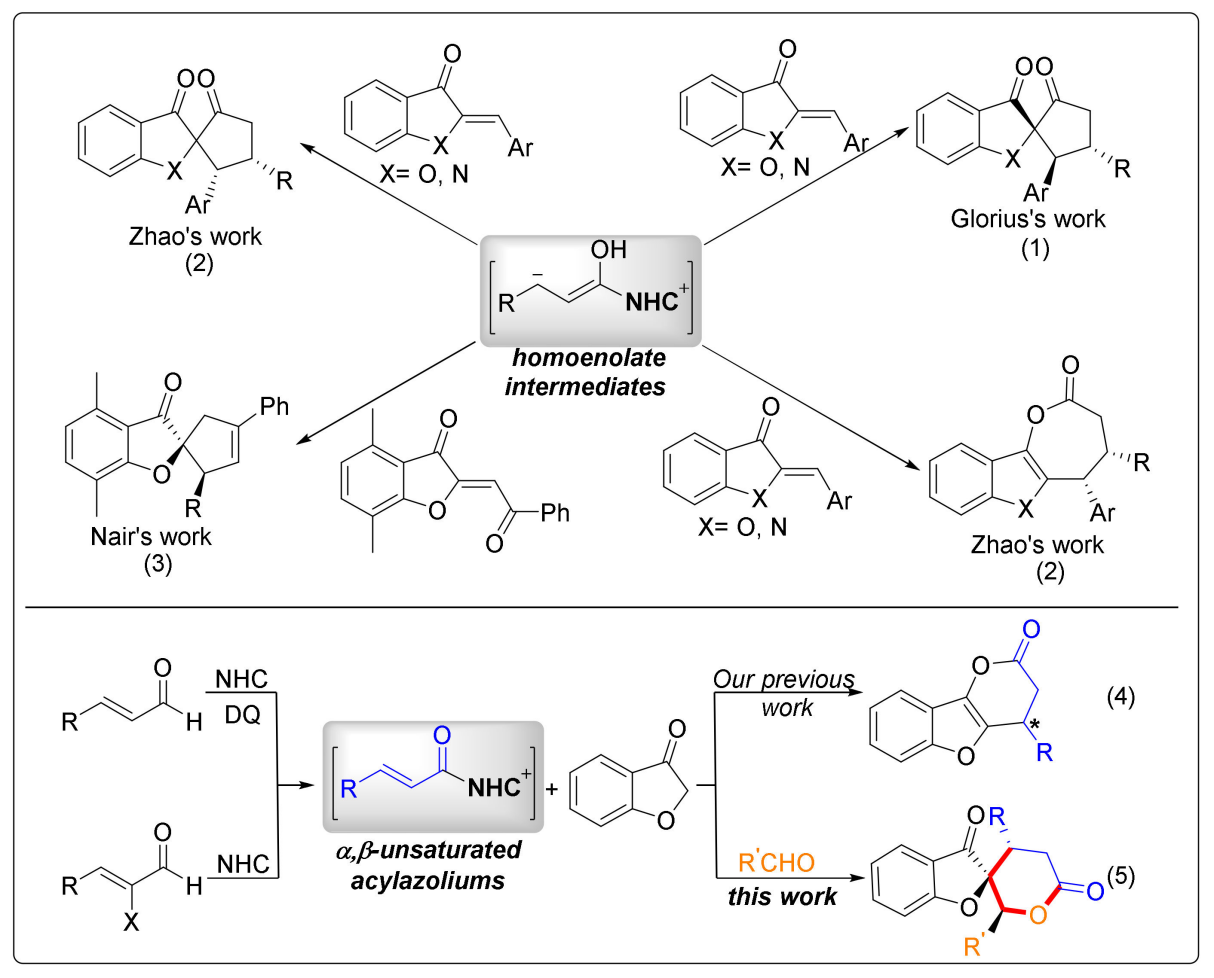
NHC-catalyzed annulation reactions of benzofuran-3-ones or their derivatives.

N-heterocyclic carbene (NHC) catalysis has emerged as one of the most popular fields for the construction of various structurally diverse carbocycles and heterocycles in the past two decades [28,29,30,31,32,33,34,35]. A wide variety of catalytic transformations proceeding via various NHC-catalyzed umpolung [36,37,38,39,40,41,42] or non-umpolung [43,44,45,46,47] strategies have been achieved. In general, there are four important modes for NHCs involved in organocatalysis, including (i) Breslow intermediates [48,49], (ii) homoenolate intermediates [50,51], (iii) enolates [52,53] and (iv) α,β-unsaturated acylazolium intermediates [54,55]. As shown in Scheme 1, the state of the art for preparing spiro-bicyclic benzofuran-3-ones utilizing NHC catalysis was represented by Glorius and co-workers; it was observed that homoenolates generated from enals by NHCs underwent facile annulation to aurones to give bis-spirofuranones (eq 1) [56]. At the same time, the Zhao group reported an elegant method for the stereoselective construction of spiro-heterocycles from enals and heterocyclic enones, in which the homoenolate intermediate plays a vital role in the control of the reaction pathway (eq 2) [57]. Simultaneously, the Nair group described the formation of cyclopentene-fused spirobenzofuran-3-ones through an NHC-involved generation of homoenolate equivalents with aurone analogs (eq 3) [58]. All these good results have caught our attention for preparing spiro-bicyclic benzofuran-3-one compounds via a homoenolate intermediate. Very recently, our group implemented the concept in the construction of benzofuran-fused δ-lactones using benzofuran-3-one substrates acting as dinucleophilic reagents to react with the α,β-unsaturated acylazoliums (eq 4) [59]. To the best of our knowledge, direct and valuable strategies using benzofuran-3-one as a simple starting bisnucleophile for the corresponding NHC-catalyzed spirocyclization reactions remain unexplored. This is part of our ongoing interest in developing new strategies for the synthesis of structurally diverse products by changing the structure of the catalyst and the substrate. Herein, we describe a very simple and convenient method for an NHC-promoted Michael–intramolecular aldol–lactonization sequence to deliver the spirocyclic products (eq 5).

## 2. Results and Discussion

We initiated our studies with the readily available benzofuran-3-one **1a** and two molecules of α-bromoenal **2a** as the starting materials in the presence of 20 mol % of NHC in toluene at room temperature for optimizing the reaction conditions (Table 1, entries 1–10).

Various NHC precursors were investigated by using Cs_2_CO_3_ as a base. In the presence of the precatalyst **A,** the desired product **3a** was formed in only 32% yield. In some cases, such as when **F**, **H** and **I** were employed, the degradation of the reactant was observed along the traces of the targeted compound (Table 1, entries 6, 8, 9); in other cases, the reactions were complicated and only small amounts of products were isolated (Table 1, entries 7, 10). Further adjustment of other NHC catalysts revealed that precatalyst **B** exhibited the highest catalytic activity, and the desired spirobenzofuranone derivative **3a** was isolated in 55% yield (Table 1, entry 2 vs. entries 1, 3–10). These results show that precatalyst B exhibited the highest catalytic activity. It is possible that due to the partially non-aromatic ring structure of B, the electrophilicity of the carbonyl attached to the partially aromatic ring structure of B was not as strong as that of other NHCs, which resulted in intermolecular aldol reaction rather than intramolecular cyclization [59]. Then, a wide range of organic and inorganic bases were investigated. DABCO, DIPEA and NaOAc could not push the reaction forward effectively and gave the isolated product in poor yields. The screening of various bases revealed that Cs_2_CO_3_ was the optimal choice (Table 1, entry 2 vs. entries 11–18). Subsequently, several solvents were further screened, but no better result was obtained (Table 1, entry 2 vs. entries 19–23). The use of 4 Å MS did give some improvement in reactivity (Table 1, entry 24). It should be noted that the desired product **3a** that we obtained in these screening cases are single diastereomers (dr >20:1). Finally, the optimal reaction conditions with respect to yield was established (see Figure S1 in Supplementary Materials).

**Scheme 2:**
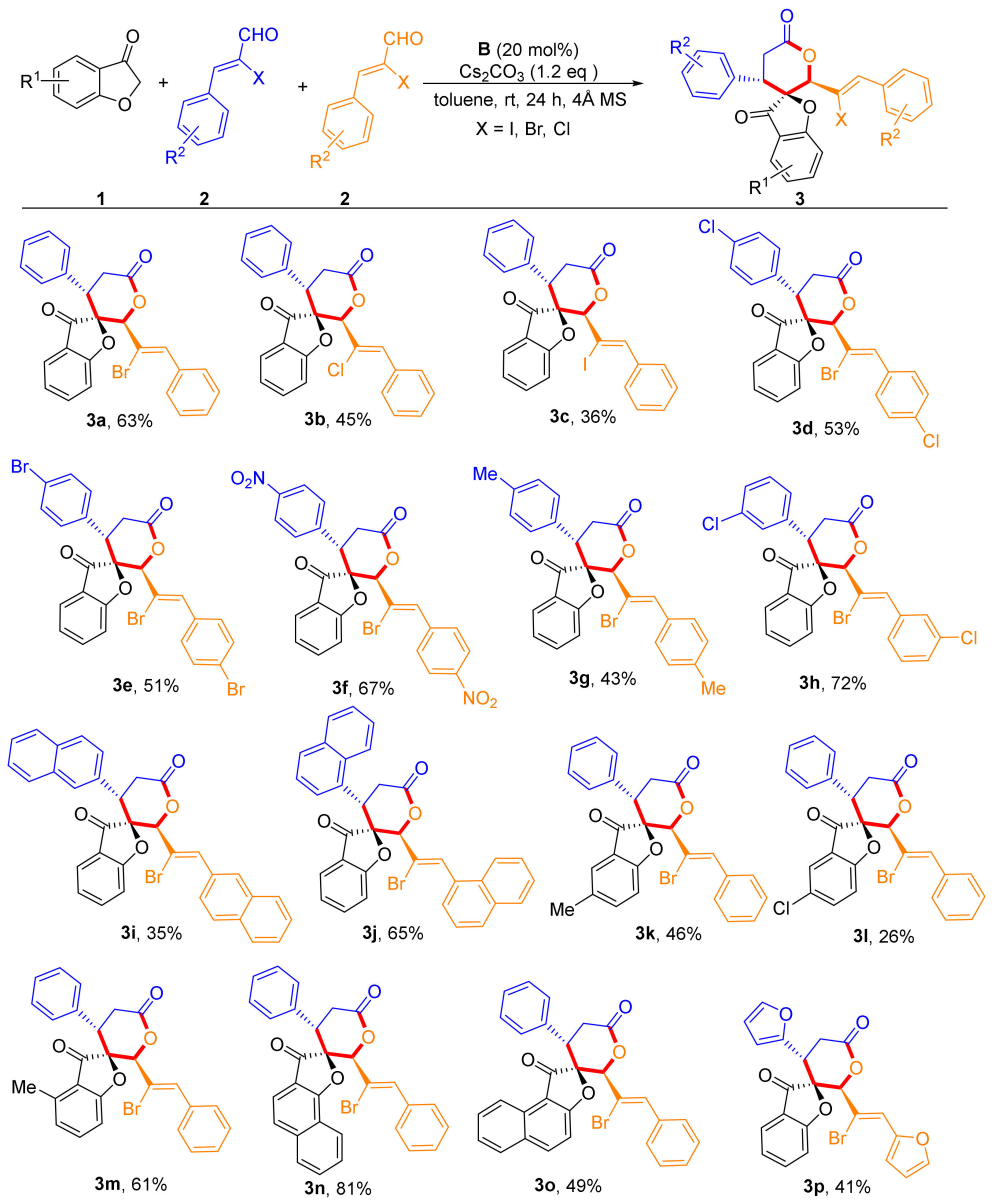
Substrate scope of the reaction between benzofuran-3-ones **1** and enals **2**. All reactions were carried out as stated in Table 1, entry 24. Isolated yields. Dr values (all products > 20:1) were determined by crude ^1^H NMR.

With the optimized reaction conditions, the generality of the reaction was further evaluated using enals **2** with various substitution patterns (see Figure S3 in Supplementary Materials). As can be seen from Scheme 2, both electron-donating and electron-withdrawing substituents all proceeded smoothly to give the desired spiro products in moderate-to-good yields under the optimized conditions (**3a**–**3o**). In addition, enals **2** bearing different halogen groups, e.g., I, Br and Cl, were all tolerated in the reaction (**3a**–**3c**). Enals bearing strong electron-withdrawing substituents, such as 4-NO_2_, could be well-tolerated to give a high yield of the corresponding product **3f**. Moreover, enals with a meta-substituent on the phenyl ring did not affect the reaction outcome and gave the cycloadduct in good yield (**3h**); however, the ortho-substituent of the enal gave the corresponding product in quite a low yield. Due to the electronic properties of naphthalene, 1-naphthaleneacrolein resulted in higher reactivity (**3i**–**3j**). Subsequently, the easily accessible benzofuran-3-ones **1** also underwent a smooth cascade reaction leading to the formation of the desired products in good yields (**3k**–**3o**). In addition, when the enals were heterocyclic-substituted, the protocol could still work well with a moderate yield (**3p**).

**Scheme 3:**
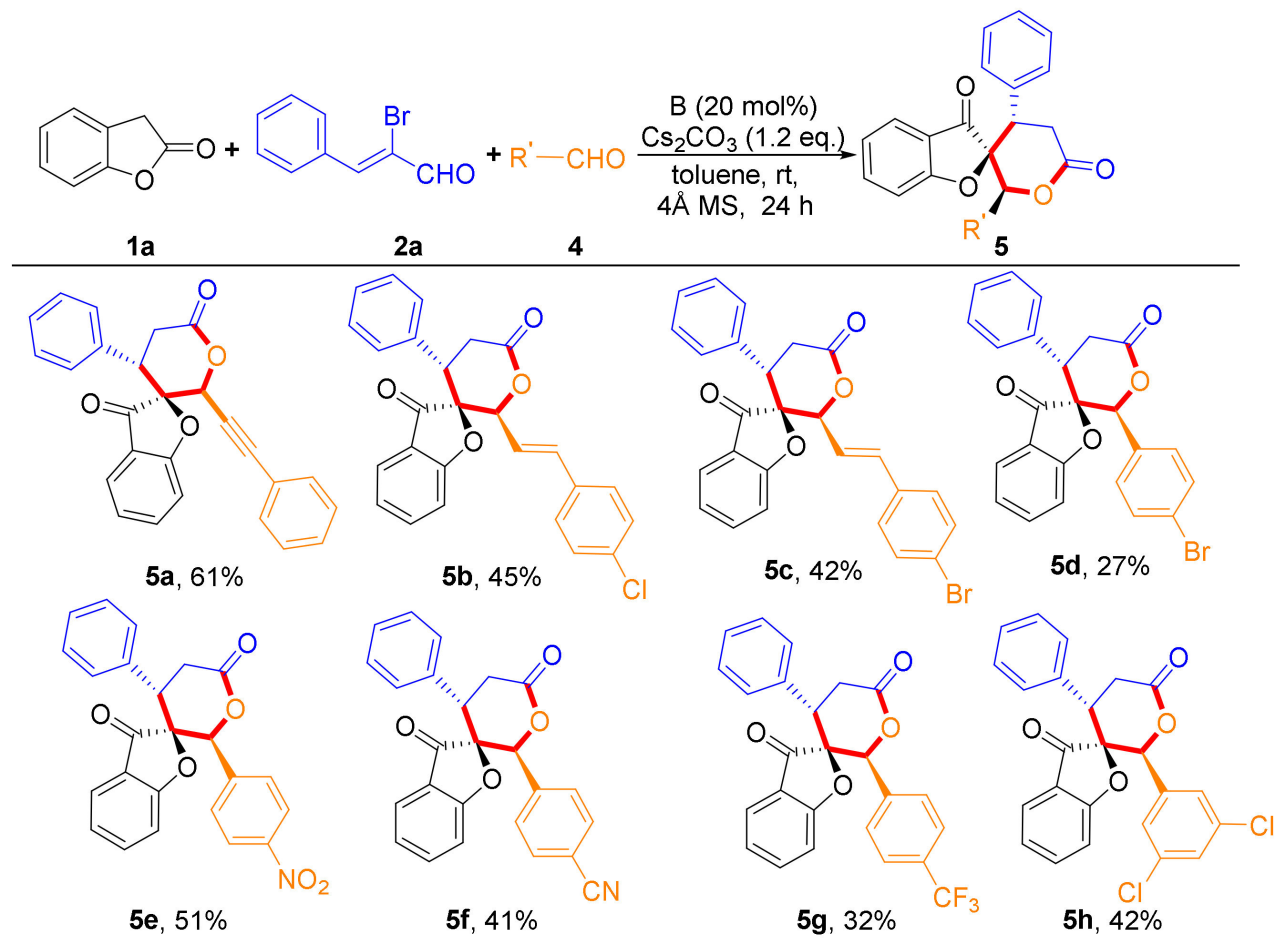
Three-component cascade reactions with two different aldehydes. All reactions were carried out as stated in Table 1, entry 24. Isolated yields. Dr values (all products > 20:1) were determined by crude ^1^H NMR.

To further extend the substrate scope of this methodology, we turned our attention to the three-component annulation with two different aldehydes. It was found that this method was successful in the preparation of spiro-bicyclic benzofuran-3-ones in moderate yields (Scheme 3). Substitution at the 4-position with electron-withdrawing groups gave the products **5d** to **5g** with moderate yields. The same result of 3,5-Dichlorobenzaldehyde could work in this cycloaddition reaction, with the corresponding product **5h**. Probably affected by steric hindrance, the ortho-substituents were not effective for this transformation.

**Scheme 4:**
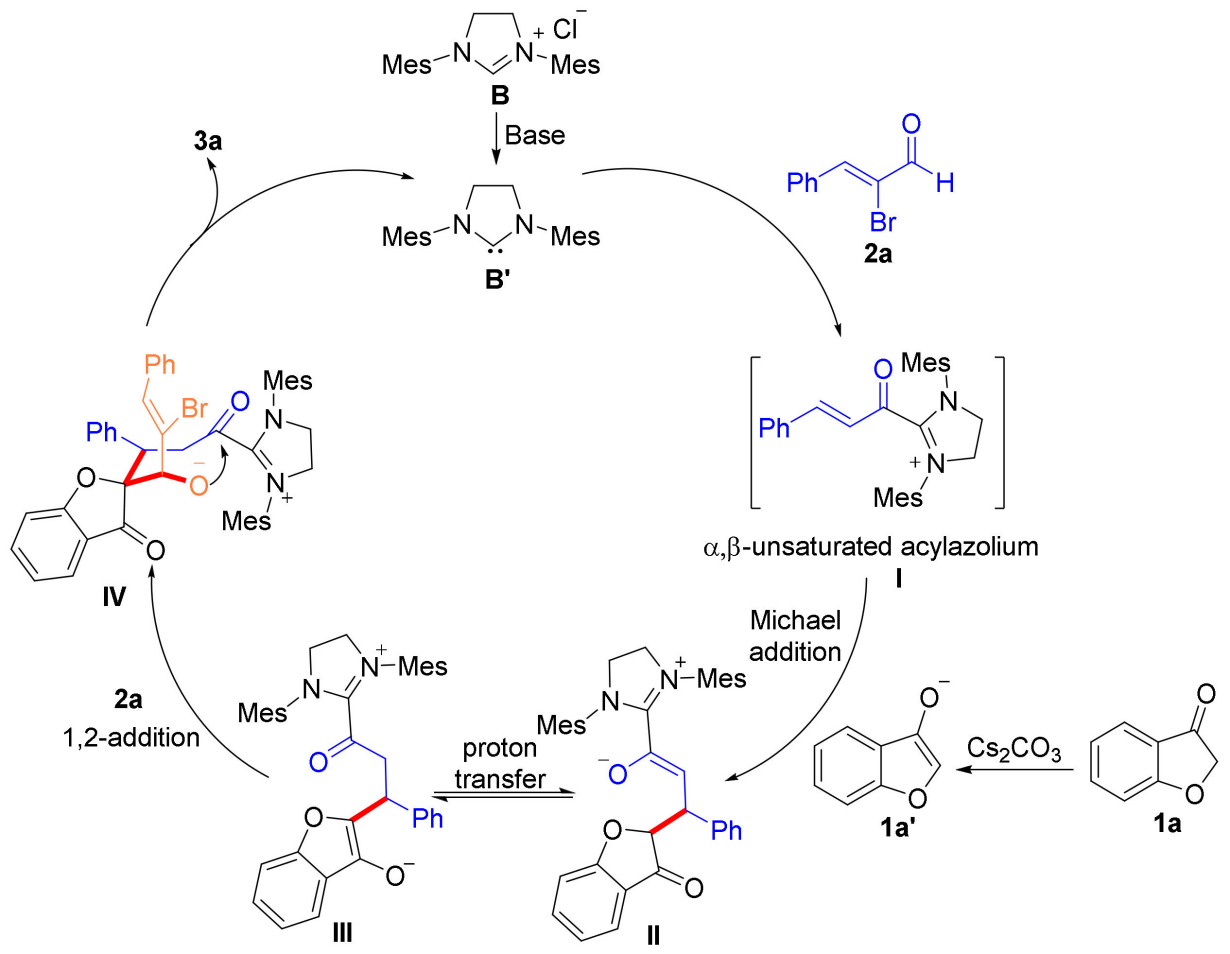
Plausible catalytic cycle.

Based on the above results of the study and previous reports [60,61,62], we propose a mechanistic rationalization for the construction of spiro-bicyclic benzofuran-3-one as follows (Scheme 4). Initially, the reaction proceeds via the free carbene nucleophilic attack on α-bromoenal **2a** and the debromination to generate the key α,β-unsaturated acylazolium intermediate **I** under basic reaction conditions. The substrate **1a** forms the enolate **1a’**. Subsequently, the Michael addition of the enolate **1a’** to intermediate **I** forms the intermediate **II**, and an intramolecular proton transfer gives the intermediate **III**. After this, intermediate **III** underwent an intermolecular aldol reaction with another molecule of α-bromoenal **2a** to form **IV**. Finally, intermediate **IV** via intramolecular lactonization results in the formation of the desired spirobenzofuranones **3**.

## 3. Materials and Methods

NMR spectra were obtained on a Bruker Avance 400 spectrometer (Bruker Corporation, Billerica, MA, USA); 400 for ^1^H NMR or 100 MHz for ^13^C NMR. ^1^H NMR spectra J-values were reported in Hz. Toluene was dried and fractionally distilled from CaH_2_. Commercially obtained reagents were used as received. Column chromatography was performed using Huanghai 300–400 mesh silica gel (Huanghai Corporation, Yantai, China) at increased pressure. HRMS (*m*/*z*) was measured using a Thermo Scientific™ Q Exactive (Thermo Scientific, New York, NY, USA).

## 4. Conclusions

In conclusion, we accomplished a novel NHC-catalyzed three-component annulation reaction for the efficient synthesis of the medicinally important spirobenzofuranone derivatives containing three contiguous stereocenters and one all-carbon quaternary spirocenter. The interception of the α-bromoenals with the catalytically generated α,β-unsaturated acylazoliums proceeds in a Michael addition–aldol reaction–cyclization sequence. This protocol can tolerate a series of available substrates and spiro-bicyclic benzofuran-3-ones were obtained in moderate-to-good yields with excellent diastereoselectivities (all products > 20:1 dr). Given the importance of the spirobenzofuranone derivatives, it is conceivable that the method outlined here may be a practical way to access these relevant molecules.

## Figures and Tables

**Table 1:** Optimization of reaction conditions ^a^.

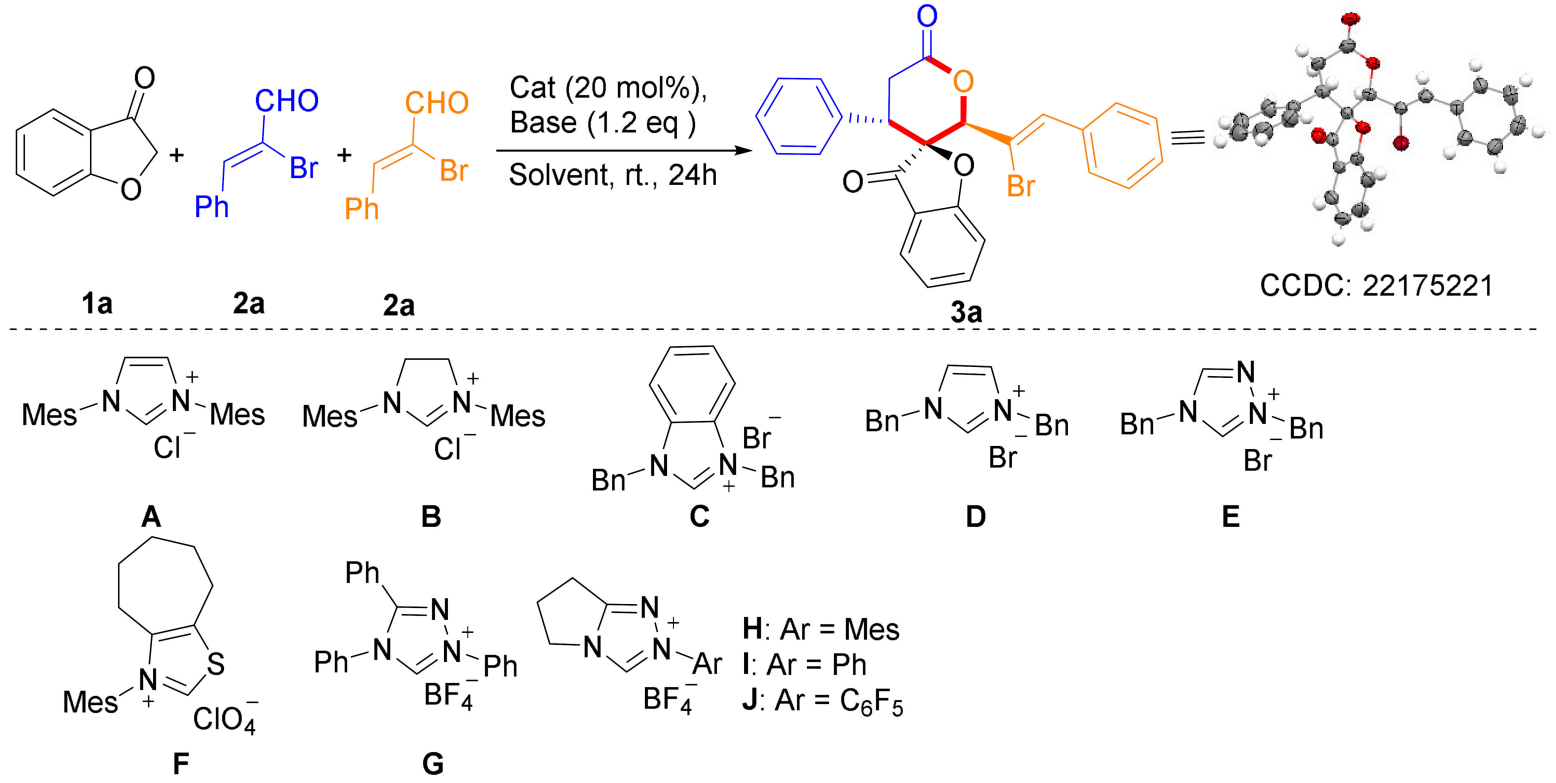				
Entry	Catalyst	Base	Solvent	Yield (%) ^b^
1	**A**	Cs_2_CO_3_	toluene	32
2	**B**	Cs_2_CO_3_	toluene	55
3	**C**	Cs_2_CO_3_	toluene	22
4	**D**	Cs_2_CO_3_	toluene	11
5	**E**	Cs_2_CO_3_	toluene	38
6	**F**	Cs_2_CO_3_	toluene	Trace ^d^
7	**G**	Cs_2_CO_3_	toluene	<5
8	**H**	Cs_2_CO_3_	toluene	Trace ^d^
9	**I**	Cs_2_CO_3_	toluene	Trace ^d^
10	**J**	Cs_2_CO_3_	toluene	<5
11	**B**	DABCO	toluene	<5
12	**B**	DBU	toluene	Trace ^d^
13	**B**	DIPEA	toluene	<5
14	**B**	DMAP	toluene	20
15	**B**	Et_3_N	toluene	34
16	**B**	NaOAc	toluene	<5
17	**B**	K_2_CO_3_	toluene	22
18	**B**	KOBu *^t^*	toluene	Trace ^d^
19	**B**	Cs_2_CO_3_	THF	34
20	**B**	Cs_2_CO_3_	DCM	12
21	**B**	Cs_2_CO_3_	CH_3_CN	Trace ^d^
22	**B**	Cs_2_CO_3_	anisole	35
23	**B**	Cs_2_CO_3_	MTBE	46
24 ^c^	**B**	Cs_2_CO_3_	toluene	63
25	**-**	Cs_2_CO_3_	toluene	Trace ^d^

^a^ Reaction conditions: **1a** (0.1 mmol), **2a** (0.25 mmol), cat (0.02 mmol), base (0.12 mmol), solvent (1.0 mL), room temperature, 24 h. Diastereoselectivity ratio (d.r) values (all products > 20:1) were determined by crude ^1^H NMR. ^b^ Isolated yields. ^c^ 4 A MS (50 mg) was used. ^d^ Degradation of the reactant and traces of the targeted compound.

## Data Availability

Data are contained within the article and Supplementary Materials.

## Supplementary Materials

The following supporting information can be downloaded at: https://www.mdpi.com/article/10.3390/molecules27185952/s1. Figure S1: General procedure for synthesis of δ-Lactone-fused spirobenzofuran-3-ones 3 and 5, Figure S2: Crystal data and structural refinement for 3a, Figure S3: Copies of NMR spectra. References [63,64,65,66,67,68,69,70,71,72,73,74,75,76,77] are cited in the Supplementary Materials.
