# Supplementary material for: Catalyst-Controlled Selectivity Switch in Three-Component Reaction: An NHC-Catalyzed Strategy for the Synthesis of δ-Lactone-Fused Spirobenzofuran-3-ones
Source: Molecules. 2022 Sep 13;27(18):5952. doi: 10.3390/molecules27185952 (PMC9503435; doi:10.3390/molecules27185952)
Supplement: Supplementary file 1 [file molecules-27-05952-s001.zip › molecules-1903145-supplementary.pdf]

Catalyst-Controlled Selectivity Switch in Three-Component Reaction: An NHC-Catalyzed Strategy for the Synthesis of δ-Lactone-Fused Spirobenzofuran-3-ones

Zhanyong Wang[1], Ting Yang[2], Dongfang Liu[3], Rongxiang Chen [1], Nan Wang[1], Hong Liu[1], Jiarong Li[1], Kaikai, Wang*[1] and Hongxin Liu*[4]

[1] School of Pharmacy, Xinxiang University, Xinxiang 453003, China; wangzhanyong@xxu.edu.cn (Z.-Y.W.); chenrx@xxu.edu.cn (R.C.); wangnan54321@163.com (N.W.); liuhong340@163.com (H.L.); lijiarong0419@163.com (J.-R.L.)

[2] Nursing College, Xinxiang University, Xinxiang 453003, China; tingy0720@126.com (T.Y.)

[3] Xinxiang Runyu Material Co., Ltd., Xinxiang 453003, China; liudongfang122920@163.com (D.L.)

[4] College of Chemistry and Materials Engineering, Wenzhou University, Wenzhou 325035, China

[5] Institute of New Materials & Industrial Technology, Wenzhou University, Wenzhou 325035, China

* Correspondence: wangkaikai@xxu.edu.cn (K.-K.W.); hongxin-107@163.com (H.-X.L.)

Supporting Information

Table of contents

1. General methods 2

2. The data of the products 3, 5 5

3. The NMR spectra of 3, 5 14

4 The data of crystal structure 3a 38

1. General methods

The benzofuran-3-ones **1** [1–5] were prepared according to the literature procedures. The α-bromoenals **2** [6–8] were prepared according to the literature procedures. The NHC precursors were synthesized according to procedure described in the literature [9–15]. Solvents and reagents were purchased from Adamas or Sigma Aldrich Column chromatography was performed using Huanghai 300-400 mesh silica gel. Dr values for all the products were determined by the integration of the alkyl proton of singlet signal as the comparison in crude ^{1}H NMR.

1. Sun, H., Ding, W., Song, X., Wang, D., Chen, M., Wang, K., Zhang, Y., Yuan, P., Ma, Y., Wang, R., et al. Synthesis of 6-hydroxyaurone analogues and evaluation of their alpha-glucosidase inhibitory and glucose consumption-promoting activity: Development of highly active 5,6-disubstituted derivatives. *Bioorg Med Chem Lett*. **2017**, *27*, 3226-3230.
2. Manjulatha, K., Srinivas, S., Mulakayala, N., Rambabu, D., Prabhakar, M., Arunasree, K.M., Alvala, M., Basaveswara Rao, M.V., and Pal, M. Ethylenediamine diacetate (EDDA) mediated synthesis of aurones under ultrasound: their evaluation as inhibitors of SIRT1. *Bioorg Med Chem Lett*. **2012**, *22*, 6160-6165.
3. Wu, Y., Guo, T., Shu, D., Zhang, W., Luan, F., Shi, L., and Guo, D. Synthesis and luminescence properties of novel 8-hydroxyquinoline derivatives and their Eu(III) complexes. *Luminescence*. **2018**, *33*, 855-862.
4. Jiménez, F., Cruz, M.d.C., Zúñiga, C., Martínez, M.A., Chamorro, G., Díaz, F., and Tamariz, J. Aryloxyacetic esters structurally related to α-Asarone as potential antifungal agents. *Med. Chem. Res.* **2009**, *19*, 33-57.
5. Rambabu, D., Srinivas, S., Manjulatha, K., Basavoju, S., Rao, M.V.B., and Pal, M. Synthesis and Structural Characterization of 2-Benzylidenebenzofuran-3-(2H)-Ones. *Mol. Cryst. Liq. Cryst.* **2013**, *577*, 83-94.
6. Song, H., Li, Y., Yao, Q.J., Jin, L., Liu, L., Liu, Y.H., and Shi, B.F. Synthesis of Axially Chiral Styrenes through Pd-Catalyzed Asymmetric C-H Olefination Enabled by an Amino Amide Transient Directing Group. *Angew. Chem., Int. Ed*. **2020**, *13*2, 6638-6642.
7. Liu, Y., Chen, J., Zhang, Z., Qin, J., Zhao, M., and Zhang, W. One-pot sequential asymmetric hydrogenation of β-aryl-β-aryloxy acroleins. *Org. Biomol. Chem*. **2016**, *14*, 7099-7102.
8. Gilley, C.B., Buller, M.J., and Kobayashi, Y. New entry to convertible isocyanides for the ugi

reaction and its application to the stereocontrolled formal total synthesis of the proteasome inhibitor Omuralide. *Org. Lett.* **2007**, *9*, 3631-3634.

9. Kyan, R., Sato, K., Mase, N., Watanabe, N., and Narumi, T. Tuning the Catalyst Reactivity of Imidazolylidene Catalysts through Substituent Effects on the N-Aryl Groups. *Org. Lett.* **2017**, *19*, 2750-2753.
10. Gülcemal, S., Gülcemal, D., Whitehead, G.F., and Xiao, J. Acceptorless Dehydrogenative Oxidation of Secondary Alcohols Catalysed by Cp* IrIII-NHC Complexes. *Chem. - Eur. J.* **2016**, *22*, 10513-10522.
11. Enders, D., Breuer, K., Kallfass, U., and Balensiefer, T. Preparation and application of 1, 3, 4-triphenyl-4, 5-dihydro-1H-1, 2, 4-triazol-5-ylidene, a stable carbene. *Synthesis* **2003**, *8*, 1292-1295.
12. Vlahakis, J.Z., Lazar, C., Crandall, I.E., and Szarek, W.A. Anti-Plasmodium activity of imidazolium and triazolium salts. *Bioorg Med Chem.* **2010**, *18*, 6184-6196.
13. Romanov-Michailidis, F., Besnard, C., and Alexakis, A. N-Heterocyclic carbene-catalyzed annulation of α-cyano-1, 4-diketones with ynals. *Org. Lett.* **2012**, *14*, 4906-4909.
14. Thomson, J.E., Campbell, C.D., Concellón, C., Duguet, N., Rix, K., Slawin, A.M., and Smith, A.D. Probing the efficiency of N-heterocyclic carbene promoted O-to C-carboxyl transfer of oxazolyl carbonates. *J. Org. Chem.* 2**008**, *73*, 2784-2791.
15. Lu, H., Lin, J.B., Liu, J.Y., and Xu, P.F. One‐Pot Asymmetric Synthesis of Quaternary Pyrroloindolones through a Multicatalytic N-Allylation/Hydroacylation Sequence. *Chem. - Eur. J.* **2014**, *20*, 11659-11663.

Typical procedure procedure for the NHC-catalyzed spirocyclization reaction of benzofuran-3-ones 1 with enals 2.

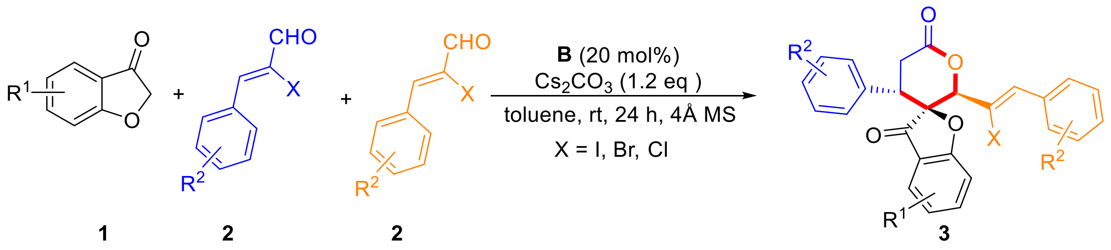

Under N_2 atmosphere, benzofuran-3-ones **1** (0.1 mmol), α-bromoenals **2** (0.25 mmol), NHC precursor **B** (0.02 mmol), 4 Å MS (50 mg), and Cs_2CO_3 (0.12 mmol) were successively added into

a 10 ml reaction tube, then dry toluene (1 ml) was added with stirring. The resulting mixture was continuously stirred at room temperature. After completion and removal of the solvent, the product **3** was obtained by column chromatography on a silica gel column eluting with petroleum ether-ethyl acetate mixture (PE:EA = 6:1).

Typical procedure for the NHC-catalyzed three-component reaction with two different aldehydes.

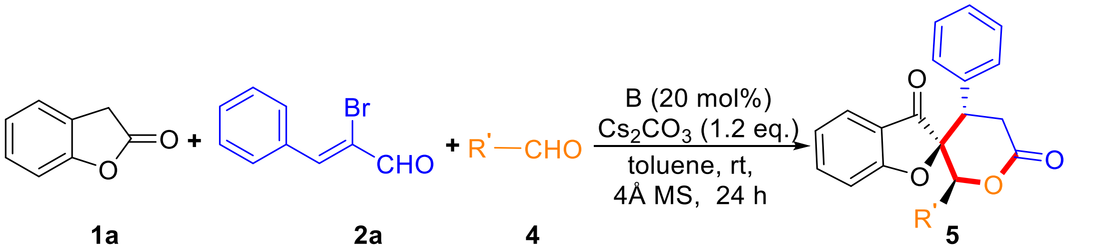

1a **2a** **4** **5**

Under N_2 atmosphere, benzofuran-3-one **1a** (0.1 mmol), α-bromoenal **2a** (0.125 mmol), **4** (0.125 mmol), NHC precursor **B** (0.02 mmol), 4 Å MS (50 mg), and Cs_2CO_3 (0.12 mmol) were successively added into a 10 ml reaction tube, then dry toluene (1 ml) was added with stirring. The resulting mixture was continuously stirred at room temperature. After completion and removal of the solvent, the product **5** was obtained by column chromatography on a silica gel column eluting with petroleum ether-ethyl acetate mixture (PE:EA = 6:1).

2. The data of the products 3, 5

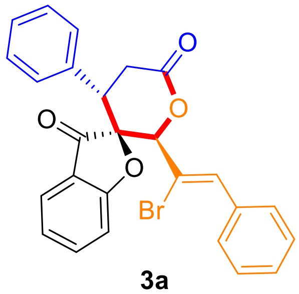

3a

3a: white solid, 63%; ^{1}H NMR (400 MHz, $CDCl_3$) δ 7.57 – 7.50 (m, 1H), 7.36 (dd, *J* = 11.7, 5.1 Hz, 3H), 7.31 – 7.26 (m, 3H), 7.20 – 7.13 (m, 4H), 7.13 – 7.06 (m, 3H), 6.93 (t, *J* = 7.5 Hz, 1H), 5.52 (s, 1H), 3.77 (dd, *J* = 12.4, 3.7 Hz, 1H), 3.56 (dd, *J* = 16.4, 12.4 Hz, 1H), 3.00 (dd, *J* = 16.4, 3.8 Hz, 1H); ^{13}C NMR (100 MHz, $CDCl_3$) δ 199.1, 172.0, 170.0, 138.5, 134.5, 134.2, 132.5, 129.0, 128.6, 128.5, 128.4, 128.22, 128.16, 124.2, 122.6, 120.9, 114.9, 112.8, 89.3, 83.4, , 44.9, 32.4; HRMS (FT-APCI): $[M+H]^+$ calcd for $C_{26}H_{20}BrO_4^+$: 475.0545; found: 475.0540.

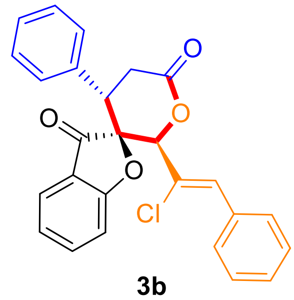

3b

3b: white solid, 45%; ^{1}H NMR (400 MHz, $CDCl_3$) δ 7.44 (t, *J* = 7.7 Hz, 1H), 7.37 (d, *J* = 7.1 Hz, 2H), 7.24 (dd, *J* = 18.4, 7.8 Hz, 4H), 7.08 (s, 3H), 7.01 (dd, *J* = 10.3, 5.9 Hz, 3H), 6.89 – 6.81 (m, 2H), 5.39 (s, 1H), 3.70 (dd, *J* = 12.5, 3.4 Hz, 1H), 3.50 (dd, *J* = 16.3, 12.6 Hz, 1H), 2.92 (dd, *J* = 16.4, 3.6 Hz, 1H); ^{13}C NMR (100 MHz, $CDCl_3$) δ 199.0, 172.0, 170.0, 138.5, 134.1, 133.4, 129.3, 128.8, 128.6, 128.5, 128.4, 128.3, 128.2, 124.2, 123.8, 122.5, 120.8, 112.7, 89.0, 82.5, 44.9, 32.4; HRMS (FT-APCI): $[M+H]^+$ calcd for $C_{26}H_{20}ClO_4^+$: 431.1050; found: 431.1047.

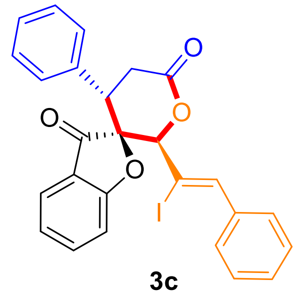

3c

3c: white solid, 36%; ^{1}H NMR (400 MHz, $CDCl_3$) δ 7.61 (t, *J* = 7.8 Hz, 1H), 7.40 (d, *J* = 7.7 Hz, 1H), 7.28 (d, *J* = 4.5 Hz, 3H), 7.22 (d, *J* = 4.9 Hz, 3H), 7.18 (d, *J* = 8.7 Hz, 1H), 7.14 (d, *J* = 6.5 Hz, 4H), 7.05 (s, 1H), 7.00 (t, *J* = 7.4 Hz, 1H), 5.41 (s, 1H), 3.76 (dd, *J* = 11.6, 3.2 Hz, 1H), 3.55 (dd, *J* = 16.3, 12.0 Hz, 1H), 3.07 (dd, *J* = 16.5, 3.8 Hz, 1H); ^{13}C NMR (100 MHz, $CDCl_3$) δ 199.1, 171.8, 169.9,

139.9, 138.6, 136.9, 134.4, 128.5, 128.41, 128.37, 128.2, 128.1, 124.3, 122.6, 121.1, 113.0, 93.7, 90.0, 84.4, 44.9, 32.6; HRMS (FT-APCI): $[M+H]^+$ calcd for $C_{26}H_{20}IO_4^+$: 523.0406; found: 523.0402.

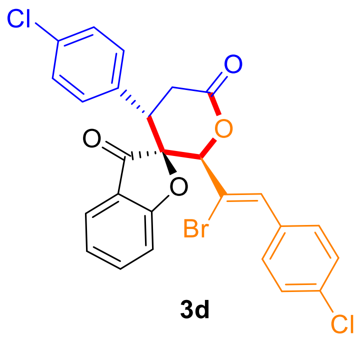

3d

3d: white solid, 53%; ^{1}H NMR (400 MHz, $CDCl_3$) δ 7.49 (t, *J* = 7.8 Hz, 1H), 7.30 (d, *J* = 7.7 Hz, 1H), 7.23 (d, *J* = 8.4 Hz, 2H), 7.18 (d, *J* = 7.1 Hz, 2H), 7.11 – 7.03 (m, 3H), 7.01 (d, *J* = 8.4 Hz, 1H), 6.96 (d, *J* = 8.3 Hz, 2H), 6.90 (t, *J* = 7.5 Hz, 1H), 5.40 (s, 1H), 3.67 (dd, *J* = 12.6, 3.4 Hz, 1H), 3.46 (dd, *J* = 16.2, 12.8 Hz, 1H), 2.89 (dd, *J* = 16.3, 3.5 Hz, 1H); ^{13}C NMR (100 MHz, $CDCl_3$) δ 198.9, 171.9, 169.5, 138.9, 134.5, 134.3, 132.74, 132.65, 131.4, 130.3, 129.7, 128.7, 128.5, 124.4, 122.9, 120.8, 115.3, 112.7, 89.1, 83.4, 44.3, 32.3; HRMS (FT-APCI): $[M+H]^+$ calcd for $C_{26}H_{18}BrCl_2O_4^+$: 542.9766; found: 542.9761.

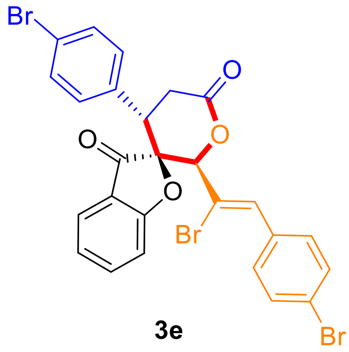

3e

3e: light yellow solid, 51%; ^{1}H NMR (400 MHz, $CDCl_3$) δ 7.49 (t, *J* = 7.7 Hz, 1H), 7.32 (dd, *J* = 12.1, 8.2 Hz, 3H), 7.23 (d, *J* = 8.3 Hz, 2H), 7.16 (d, *J* = 8.3 Hz, 2H), 7.01 (d, *J* = 10.6 Hz, 2H), 6.91 (t, *J* = 8.5 Hz, 3H), 5.40 (s, 1H), 3.65 (dd, *J* = 12.6, 3.4 Hz, 1H), 3.45 (dd, *J* = 16.2, 12.7 Hz, 1H), 2.88 (dd, *J* = 16.3, 3.6 Hz, 1H); ^{13}C NMR (100 MHz, $CDCl_3$) δ 198.8, 171.9, 169.5, 138.9, 133.2, 131.7, 131.5, 131.4, 130.5, 130.0, 124.4, 122.9, 122.7, 122.5, 120.8, 115.4, 112.7, 89.0, 83.4, 44.3, 32.3; HRMS (FT-APCI): $[M+H]^+$ calcd for $C_{26}H_{18}Br_3O_4^+$: 630.8755; found: 630.8745.

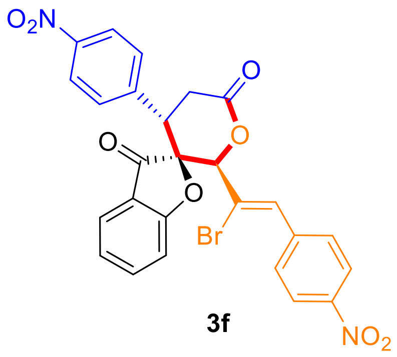

3f

3f: white solid, 67%; ^{1}H NMR (400 MHz, $CDCl_3$) δ 8.18 (d, *J* = 8.3 Hz, 2H), 8.06 (d, *J* = 8.3 Hz, 2H), 7.62 (t, *J* = 7.7 Hz, 1H), 7.53 (d, *J* = 8.4 Hz, 2H), 7.39 (d, *J* = 7.7 Hz, 1H), 7.32 (d, *J* = 7.5 Hz, 3H),

7.14 (d, J = 8.4 Hz, 1H), 7.02 (t, J = 7.4 Hz, 1H), 5.57 (s, 1H), 3.93 (d, J = 10.7 Hz, 1H), 3.77 – 3.56 (m, 1H), 3.03 (dd, J = 16.2, 2.6 Hz, 1H); ^{13}C NMR (100 MHz, $CDCl_3$) δ 198.5, 171.9, 168.7, 147.7, 147.4, 141.3, 140.7, 139.4, 130.7, 129.8, 129.4, 124.5, 123.6, 123.5, 123.4, 120.6, 118.1, 112.7, 88.8, 83.3, 44.7, 32.0; HRMS (FT-APCI): $[M+ H]^+$ calcd for $C_{26}H_{18}BrN_2O_8^+$: 565.0247; found: 565.0236.

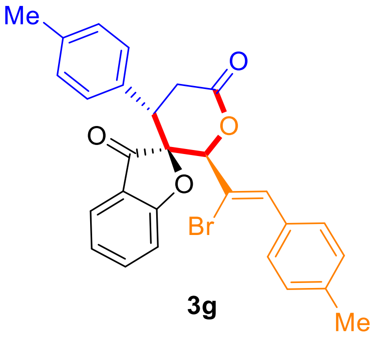

3g

3g: white solid, 43%; ^{1}H NMR (400 MHz, $CDCl_3$) δ 7.46 (t, J = 7.8 Hz, 1H), 7.30 (d, J = 7.7 Hz, 1H), 7.21 (s, 2H), 7.01 (d, J = 7.4 Hz, 4H), 6.90 (d, J = 8.1 Hz, 4H), 6.87 (t, J = 7.5 Hz, 1H), 5.41 (s, 1H), 3.63 (dd, J = 11.6, 3.8 Hz, 1H), 3.42 (dd, J = 16.5, 11.7 Hz, 1H), 2.94 (dd, J = 16.5, 3.9 Hz, 1H), 2.23 (s, 3H), 2.15 (s, 3H); ^{13}C NMR (100 MHz, $CDCl_3$) δ 199.0, 171.9, 170.1, 138.7, 138.6, 137.9, 132.7, 131.6, 131.3, 129.2, 129.0, 128.9, 128.3, 124.3, 122.5, 121.0, 114.1, 112.8, 89.3, 83.5, 44.5, 32.6, 21.3, 21.0; HRMS (FT-APCI): $[M+ H]^+$ calcd for $C_{28}H_{24}BrO_4^+$: 503.0858; found: 503.0851.

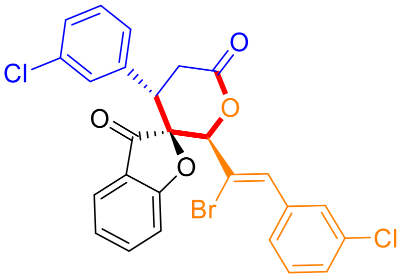

3h

3h: white solid, 72%; ^{1}H NMR (400 MHz, $CDCl_3$) δ 7.60 (t, J = 7.8 Hz, 1H), 7.45 – 7.36 (m, 2H), 7.26 (s, 3H), 7.14 (dd, J = 13.0, 4.1 Hz, 5H), 7.00 (t, J = 4.8 Hz, 2H), 5.52 (s, 1H), 3.78 (dd, J = 12.7, 2.7 Hz, 1H), 3.66 – 3.49 (m, 1H), 2.99 (dd, J = 16.3, 2.8 Hz, 1H); ^{13}C NMR (100 MHz, $CDCl_3$) δ 198.8, 171.9, 169.4, 138.9, 136.1, 134.4, 134.1, 131.2, 129.7, 129.5, 128.8, 128.7, 128.6, 128.5, 127.2, 126.4, 124.4, 122.9, 120.8, 116.2, 112.8, 88.9, 44.5, 32.1; HRMS (FT-APCI): $[M+ H]^+$ calcd for $C_{26}H_{18}BrCl_2O_4^+$: 542.9766; found: 542.9761.

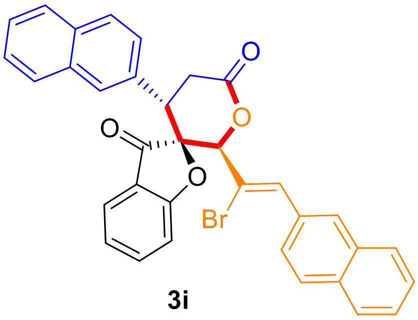

3i

3i: white solid, 35%; ^{1}H NMR (400 MHz, $CDCl_3$) δ 7.77 – 7.71 (m, 2H), 7.69 (d, J = 8.6 Hz, 1H), 7.58 (s, 1H), 7.54 (t, J = 7.8 Hz, 1H), 7.46 – 7.41 (m, 2H), 7.37 (t, J = 8.2 Hz, 2H), 7.19 (dd, J = 18.9, 7.3 Hz, 4H), 7.13 (dd, J = 12.2, 8.1 Hz, 3H), 6.93 (t, J = 7.5 Hz, 1H), 6.79 (d, J = 16.0 Hz, 1H), 6.20 (dd, J

= 15.9, 7.0 Hz, 1H), 5.37 (d, *J* = 7.0 Hz, 1H), 3.73 (dd, *J* = 10.8, 4.8 Hz, 1H), 3.45 (dd, *J* = 16.7, 11.0 Hz, 1H), 3.08 (dd, *J* = 16.5, 4.7 Hz, 1H); ^{13}C NMR (100 MHz, $CDCl_3$) δ 198.7, 171.6, 170.6, 138.7, 136.0, 134.9, 133.29, 133.25, 132.8, 128.5, 128.3, 128.2, 128.14, 128.11, 127.6, 127.4, 126.42, 126.39, 124.3, 123.3, 122.6, 120.9, 119.9, 112.9, 89.9, 81.3, 44.6, 32.8; HRMS (FT-APCI): $[M+ H]^+$ calcd for $C_{34}H_{24}BrO_4^+$: 575.0858; found: 575.0855.

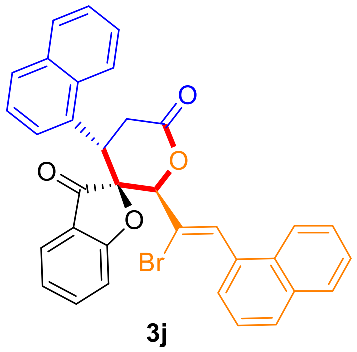

3j

3j: white solid, 65%; ^{1}H NMR (400 MHz, $CDCl_3$) δ 8.12 (d, *J* = 8.6 Hz, 1H), 7.70 (dd, *J* = 14.2, 8.2 Hz, 2H), 7.63 – 7.57 (m, 2H), 7.55 (s, 1H), 7.50 (d, *J* = 8.3 Hz, 1H), 7.40 – 7.33 (m, 3H), 7.32 – 7.28 (m, 3H), 7.23 (d, *J* = 7.7 Hz, 1H), 7.19 – 7.13 (m, 3H), 6.83 (d, *J* = 8.4 Hz, 1H), 6.70 (t, *J* = 7.4 Hz, 1H), 5.70 (s, 1H), 4.75 (dd, *J* = 12.5, 3.0 Hz, 1H), 3.71 (dd, *J* = 16.0, 12.9 Hz, 1H), 2.96 (dd, *J* = 16.2, 3.2 Hz, 1H); ^{13}C NMR (100 MHz, $CDCl_3$) δ 199.2, 171.8, 170.4, 138.2, 133.5, 133.3, 132.6, 132.3, 131.9, 130.8, 130.7, 128.9, 128.7, 128.6, 128.5, 126.6, 126.32, 126.25, 126.04, 125.97, 125.7, 125.2, 125.1, 124.4, 124.0, 123.3, 122.5, 120.5, 118.9, 112.7, 90.4, 83.2, 39.4, 33.6; HRMS (FT-APCI): $[M+ H]^+$ calcd for $C_{34}H_{24}BrO_4^+$: 575.0858; found: 575.0851.

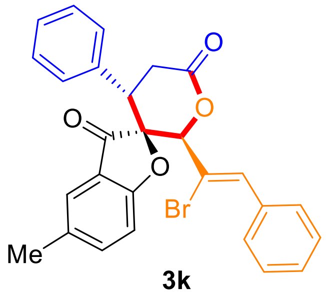

3k

3k: white solid, 46%; ^{1}H NMR (400 MHz, $CDCl_3$) δ 7.41 – 7.32 (m, 3H), 7.29 (dd, *J* = 8.9, 3.2 Hz, 3H), 7.21 – 7.16 (m, 3H), 7.14 (d, *J* = 8.1 Hz, 2H), 7.12 – 7.07 (m, 2H), 6.98 (d, *J* = 8.5 Hz, 1H), 5.49 (s, 1H), 3.74 (dd, *J* = 12.1, 3.7 Hz, 1H), 3.55 (dd, *J* = 16.4, 12.2 Hz, 1H), 3.00 (dd, *J* = 16.4, 3.8 Hz, 1H), 2.22 (s, 3H); ^{13}C NMR (100 MHz, $CDCl_3$) δ 199.0, 170.4, 170.1, 139.9, 134.5, 134.4, 132.6, 132.2, 129.1, 128.51, 128.46, 128.4, 128.1, 123.6, 120.9, 115.0, 112.4, 89.4, 83.6, 44.9, 32.5, 20.5; HRMS (FT-APCI): $[M+ H]^+$ calcd for $C_{27}H_{22}BrO_4^+$: 489.0701; found: 489.0698.

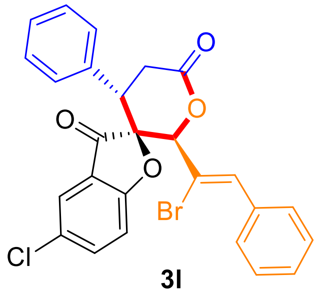

3l

3l: white solid, 26%; ^{1}H NMR (400 MHz, $CDCl_3$) δ 7.47 (d, *J* = 8.5 Hz, 1H), 7.41 (d, *J* = 6.0 Hz, 2H), 7.31 (d, *J* = 6.0 Hz, 4H), 7.21 – 7.17 (m, 4H), 7.06 (dd, *J* = 13.8, 6.7 Hz, 3H), 5.50 (s, 1H), 3.76 (dd, *J* = 12.7, 3.2 Hz, 1H), 3.56 (dd, *J* = 16.1, 12.9 Hz, 1H), 2.98 (dd, *J* = 16.3, 3.3 Hz, 1H); ^{13}C NMR (100 MHz, $CDCl_3$) δ 198.0, 170.3, 169.8, 138.4, 134.3, 133.8, 132.5, 129.1, 128.7, 128.6, 128.5, 128.4, 128.2, 128.1, 123.5, 122.1, 114.3, 114.0, 90.5, 83.4, 45.0, 32.3; HRMS (FT-APCI): $[M+H]^+$ calcd for $C_{26}H_{19}BrClO_4^+$: 509.0155; found: 509.0152.

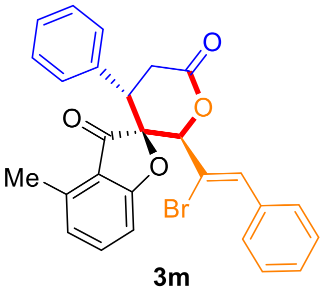

3m

3m: white solid, 61%; ^{1}H NMR (400 MHz, $CDCl_3$) δ 7.33 (dd, *J* = 5.7, 4.1 Hz, 3H), 7.30 – 7.26 (m, 3H), 7.18 (dd, *J* = 4.8, 3.0 Hz, 4H), 7.10 (dd, *J* = 9.1, 6.5 Hz, 3H), 6.83 (t, *J* = 7.5 Hz, 1H), 5.50 (s, 1H), 3.78 (dd, *J* = 12.1, 3.8 Hz, 1H), 3.57 (dd, *J* = 16.4, 12.2 Hz, 1H), 3.03 (dd, *J* = 16.5, 3.9 Hz, 1H), 2.32 (s, 3H); ^{13}C NMR (100 MHz, $CDCl_3$) δ 199.4, 170.7, 170.1, 138.9, 134.5, 134.4, 132.7, 128.9, 128.50, 128.47, 128.3, 128.20, 128.17, 123.0, 122.5, 121.4, 120.3, 115.3, 89.1, 83.5, 44.8, 32.4, 14.3; HRMS (FT-APCI): $[M+H]^+$ calcd for $C_{27}H_{22}BrO_4^+$: 489.0701; found: 489.0696.

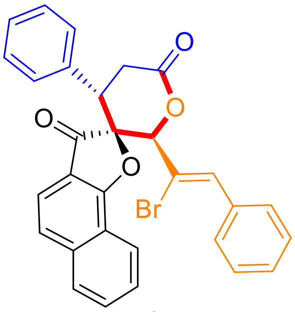

3n

3n: white solid, 81%; ^{1}H NMR (400 MHz, $CDCl_3$) δ 8.31 (d, *J* = 8.2 Hz, 1H), 7.82 (d, *J* = 8.1 Hz, 1H), 7.71 (t, *J* = 7.6 Hz, 1H), 7.62 (t, *J* = 7.6 Hz, 1H), 7.31 (d, *J* = 8.5 Hz, 1H), 7.22 (d, *J* = 6.2 Hz, 3H), 7.18 (d, *J* = 5.4 Hz, 3H), 7.12 (d, *J* = 5.4 Hz, 6H), 5.59 (s, 1H), 3.90 (dd, *J* = 12.1, 3.5 Hz, 1H), 3.64 (dd, *J* = 16.4, 12.3 Hz, 1H), 3.08 (dd, *J* = 16.5, 3.6 Hz, 1H); ^{13}C NMR (100 MHz, $CDCl_3$) δ 197.9, 172.8, 170.0, 138.8, 134.4, 134.3, 132.9, 131.2, 128.9, 128.52, 128.49, 128.45, 128.2, 128.1, 127.2, 123.2, 122.3, 120.9, 118.3, 115.8, 115.3, 90.6, 83.5, 44.7, 32.5; HRMS (FT-APCI): $[M+H]^+$ calcd for $C_{30}H_{22}BrO_4^+$: 525.0701; found: 525.0696.

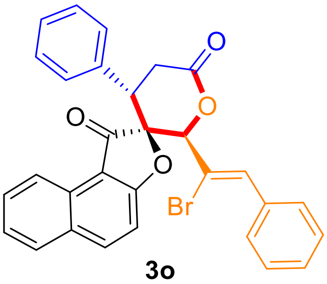

3o

3o: light yellow solid, 49%; ^{1}H NMR (400 MHz, $CDCl_3$) δ 8.47 (d, *J* = 8.2 Hz, 1H), 8.00 (d, *J* = 9.0 Hz, 1H), 7.74 (d, *J* = 8.1 Hz, 1H), 7.57 (t, *J* = 7.6 Hz, 1H), 7.45 – 7.39 (m, 1H), 7.34 – 7.29 (m, 2H), 7.22 (t, *J* = 6.6 Hz, 4H), 7.13 (dd, *J* = 15.1, 7.9 Hz, 5H), 7.07 (dd, *J* = 8.1, 5.1 Hz, 1H), 5.61 (s, 1H), 3.82 (dd, *J* = 12.1, 3.7 Hz, 1H), 3.65 (dd, *J* = 16.4, 12.1 Hz, 1H), 3.05 (dd, *J* = 16.5, 3.8 Hz, 1H); ^{13}C NMR (100 MHz, $CDCl_3$) δ 198.1, 174.7, 170.1, 140.7, 134.4, 134.3, 132.7, 130.1, 129.3, 129.0, 128.62, 128.56, 128.5, 128.39, 128.36, 128.2, 128.1, 125.7, 123.0, 115.0, 113.4, 113.0, 89.8, 83.7, 44.8, 32.5; HRMS (FT-APCI): $[M+ H]^+$ calcd for $C_{30}H_{22}BrO_4^+$: 525.0701; found: 525.0698.

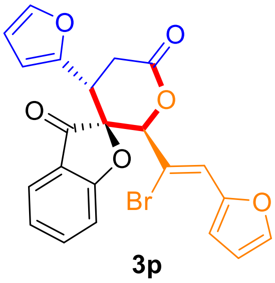

3p

3p: light yellow solid, 41%; ^{1}H NMR (400 MHz, $CDCl_3$) δ 7.64 – 7.57 (m, 2H), 7.37 (d, *J* = 23.4 Hz, 2H), 7.14 (dd, *J* = 22.1, 13.7 Hz, 3H), 6.96 (d, *J* = 3.0 Hz, 1H), 6.35 (d, *J* = 32.1 Hz, 2H), 6.17 (d, *J* = 2.5 Hz, 1H), 5.53 (s, 1H), 3.64 (t, *J* = 6.1 Hz, 1H), 3.28 – 3.13 (m, 2H); ^{13}C NMR (100 MHz, $CDCl_3$) δ 196.8, 171.1, 167.8, 149.6, 148.3, 143.1, 142.9, 138.6, 124.8, 122.9, 120.3, 113.4, 112.9, 112.0, 111.5, 110.6, 109.6, 86.3, 82.9, 38.9, 31.5; HRMS (FT-APCI): $[M+ H]^+$ calcd for $C_{22}H_{16}BrO_6^+$: 455.0130; found: 455.0127.

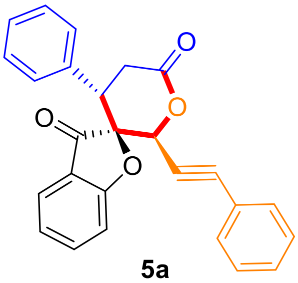

5a

5a: white solid, 61%; ^{1}H NMR (400 MHz, $CDCl_3$) δ 7.61 (t, *J* = 7.8 Hz, 1H), 7.47 (d, *J* = 7.7 Hz, 1H), 7.29 (d, *J* = 7.4 Hz, 1H), 7.20 (t, *J* = 8.2 Hz, 6H), 7.14 (dd, *J* = 8.3, 4.8 Hz, 4H), 7.02 (t, *J* = 7.5 Hz, 1H), 5.55 (s, 1H), 3.90 (dd, *J* = 11.2, 5.2 Hz, 1H), 3.46 (dd, *J* = 16.9, 11.3 Hz, 1H), 3.10 (dd, *J* = 17.0, 5.3 Hz, 1H); ^{13}C NMR (100 MHz, $CDCl_3$) δ 197.9, 172.0, 169.4, 138.7, 134.6, 131.8, 129.3, 128.6, 128.3, 124.5, 122.6, 121.0, 120.9, 113.1, 90.4, 88.0, 79.6, 72.0, 43.6, 32.7; HRMS (FT-APCI): $[M+ H]^+$ calcd for $C_{26}H_{19}O_4^+$: 395.1283; found: 395.1277.

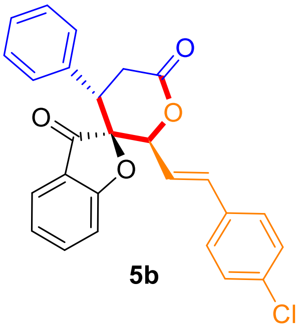

5b

5b: white solid, 45%; ^{1}H NMR (400 MHz, $CDCl_3$) δ 7.57 (t, *J* = 7.3 Hz, 1H), 7.38 (d, *J* = 7.6 Hz, 1H), 7.20 (d, *J* = 8.2 Hz, 5H), 7.15 (s, 1H), 7.10 (d, *J* = 8.2 Hz, 4H), 6.96 (t, *J* = 7.4 Hz, 1H), 6.59 (d, *J* =

15.9 Hz, 1H), 6.04 (dd, J = 16.0, 7.0 Hz, 1H), 5.30 (d, J = 6.9 Hz, 1H), 3.69 (dd, J = 10.7, 4.8 Hz, 1H), 3.42 (dd, J = 16.7, 10.7 Hz, 1H), 3.06 (dd, J = 16.7, 4.8 Hz, 1H); ^{13}C NMR (100 MHz, $CDCl_3$) δ 198.6, 171.5, 170.5, 138.7, 134.8, 134.6, 134.3, 133.9, 128.8, 128.6, 128.3, 128.2, 128.0, 124.4, 122.7, 120.8, 120.3, 112.9, 89.7, 81.0, 44.6, 32.8; HRMS (FT-APCI): [M+ H]$^+$ calcd for $C_{26}H_{20}ClO_4^+$: 431.1050; found: 431.1042.

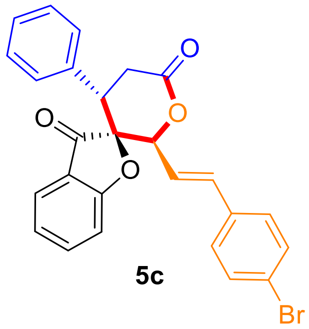

5c

5c: white solid, 42%; ^{1}H NMR (400 MHz, $CDCl_3$) δ 7.59 – 7.54 (m, 1H), 7.39 – 7.32 (m, 3H), 7.25 – 7.17 (m, 3H), 7.13 (d, J = 8.4 Hz, 1H), 7.12 – 7.07 (m, 2H), 7.04 (d, J = 8.4 Hz, 2H), 6.96 (t, J = 7.4 Hz, 1H), 6.57 (d, J = 15.9 Hz, 1H), 6.06 (dd, J = 15.9, 6.9 Hz, 1H), 5.30 (d, J = 6.9 Hz, 1H), 3.69 (dd, J = 10.7, 4.8 Hz, 1H), 3.42 (dd, J = 16.7, 10.8 Hz, 1H), 3.06 (dd, J = 16.7, 4.8 Hz, 1H); ^{13}C NMR (100 MHz, $CDCl_3$) δ 198.6, 171.5, 170.4, 138.8, 134.8, 134.6, 134.3, 131.7, 128.6, 128.3, 128.2, 124.4, 122.7, 122.5, 120.8, 120.4, 112.9, 89.7, 81.0, 44.5, 32.8; HRMS (FT-APCI): [M+ H]$^+$ calcd for $C_{26}H_{20}BrO_4^+$: 475.0545; found: 475.0536.

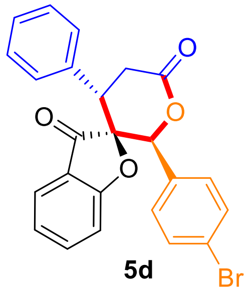

5d

5d: white solid, 27%; ^{1}H NMR (400 MHz, $CDCl_3$) δ 7.48 – 7.42 (m, 1H), 7.32 – 7.26 (m, 3H), 7.25 – 7.21 (m, 3H), 7.13 – 7.06 (m, 4H), 6.88 (t, J = 8.6 Hz, 2H), 5.75 (s, 1H), 3.71 (dd, J = 10.7, 4.5 Hz, 1H), 3.47 (dd, J = 16.5, 10.7 Hz, 1H), 3.13 (dd, J = 16.5, 4.6 Hz, 1H); ^{13}C NMR (100 MHz, $CDCl_3$) δ 198.5, 171.2, 170.7, 138.7, 134.8, 131.13, 131.07, 129.2, 128.6, 128.5, 128.3, 128.2, 124.0, 123.0, 122.5, 120.8, 112.7, 89.9, 81.8, 44.9, 33.0; HRMS (FT-APCI): [M+ H]$^+$ calcd for $C_{24}H_{18}BrO_4^+$: 449.0388; found: 449.0380.

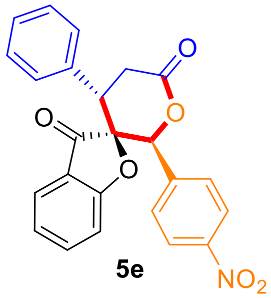

5e

5e: white solid, 51%; ^{1}H NMR (400 MHz, $CDCl_3$) δ 8.02 (d, *J* = 8.2 Hz, 2H), 7.43 (dd, *J* = 13.5, 7.9 Hz, 3H), 7.25 (d, *J* = 7.7 Hz, 4H), 7.11 (d, *J* = 3.0 Hz, 2H), 6.88 (dd, *J* = 15.4, 8.0 Hz, 2H), 5.90 (s, 1H), 3.74 (dd, *J* = 10.5, 4.4 Hz, 1H), 3.49 (dd, *J* = 16.6, 10.6 Hz, 1H), 3.17 (dd, *J* = 16.6, 4.4 Hz, 1H); ^{13}C NMR (100 MHz, $CDCl_3$) δ 198.2, 171.0, 170.1, 148.0, 139.1, 139.0, 134.5, 128.7, 128.44, 128.41, 128.3, 124.1, 123.1, 122.8, 112.6, 89.7, 81.3, 45.0, 33.0; HRMS (FT-APCI): $[M+ H]^+$ calcd for $C_{24}H_{18}NO_6^+$: 416.1134; found: 416.1126.

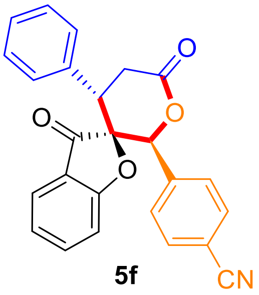

5f

5f: white solid, 41%; ^{1}H NMR (400 MHz, $CDCl_3$) δ 7.48 – 7.41 (m, 3H), 7.34 (d, *J* = 8.3 Hz, 2H), 7.24 (dd, *J* = 8.8, 5.4 Hz, 4H), 7.13 – 7.06 (m, 2H), 6.93 – 6.82 (m, 2H), 5.84 (s, 1H), 3.73 (dd, *J* = 10.7, 4.4 Hz, 1H), 3.48 (dd, *J* = 16.5, 10.7 Hz, 1H), 3.15 (dd, *J* = 16.5, 4.5 Hz, 1H); ^{13}C NMR (100 MHz, $CDCl_3$) δ 198.2, 171.1, 170.3, 138.9, 137.2, 134.6, 131.7, 128.7, 128.4, 128.24, 128.15, 124.1, 122.8, 120.7, 118.2, 112.8, 112.6, 89.7, 81.5, 44.9, 33.0; HRMS (FT-APCI): $[M+ H]^+$ calcd for $C_{25}H_{18}NO_4^+$: 396.1236; found: 396.1229.

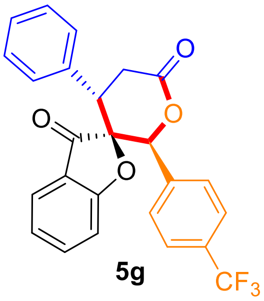

5g

5g: white solid, 32%; ^{1}H NMR (400 MHz, $CDCl_3$) δ 7.43 (dd, *J* = 12.1, 4.9 Hz, 3H), 7.35 (d, *J* = 8.3 Hz, 2H), 7.26 – 7.19 (m, 4H), 7.10 (dd, *J* = 6.6, 2.6 Hz, 2H), 6.90 – 6.82 (m, 2H), 5.85 (s, 1H), 3.73 (dd, *J* = 10.8, 4.5 Hz, 1H), 3.50 (dd, *J* = 16.5, 10.8 Hz, 1H), 3.15 (dd, *J* = 16.5, 4.5 Hz, 1H); ^{13}C NMR (100 MHz, $CDCl_3$) δ 198.4, 171.2, 170.5, 138.7, 136.0, 134.7, 130.9 (q, *J* = 33 Hz), 128.6, 128.30, 128.27, 127.9, 124.9 (q, *J* = 4 Hz), 124.0, 123.7 (q, *J* = 270 Hz), 122.6, 120.7, 112.6, 89.8, 81.7, 44.9, 33.0; HRMS (FT-APCI): $[M+ H]^+$ calcd for $C_{25}H_{18}F_3O_4^+$: 439.1157; found: 439.1148.

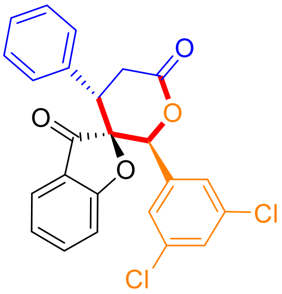

5h

5h: white solid, 42%; ^{1}H NMR (400 MHz, $CDCl_3$) δ 7.48 (t, *J* = 7.8 Hz, 1H), 7.32 (d, *J* = 7.7 Hz, 1H), 7.25 – 7.21 (m, 3H), 7.13 (s, 3H), 7.12 – 7.07 (m, 2H), 6.92 (dd, *J* = 14.0, 7.8 Hz, 2H), 5.71 (s, 1H), 3.70 (dd, *J* = 10.5, 4.5 Hz, 1H), 3.45 (dd, *J* = 16.6, 10.6 Hz, 1H), 3.14 (dd, *J* = 16.6, 4.6 Hz, 1H); ^{13}C NMR (100 MHz, $CDCl_3$) δ 198.2, 171.1, 170.2, 138.9, 135.3, 134.62, 134.58, 129.1, 128.7, 128.4, 128.3, 126.0, 124.2, 122.7, 120.8, 112.7, 89.5, 81.0, 44.8, 32.9; HRMS (FT-APCI): $[M+H]^+$ calcd for $C_{24}H_{17}Cl_2O_4^+$: 439.0504; found: 439.0496.

3. **The NMR spectra of 3, 5**

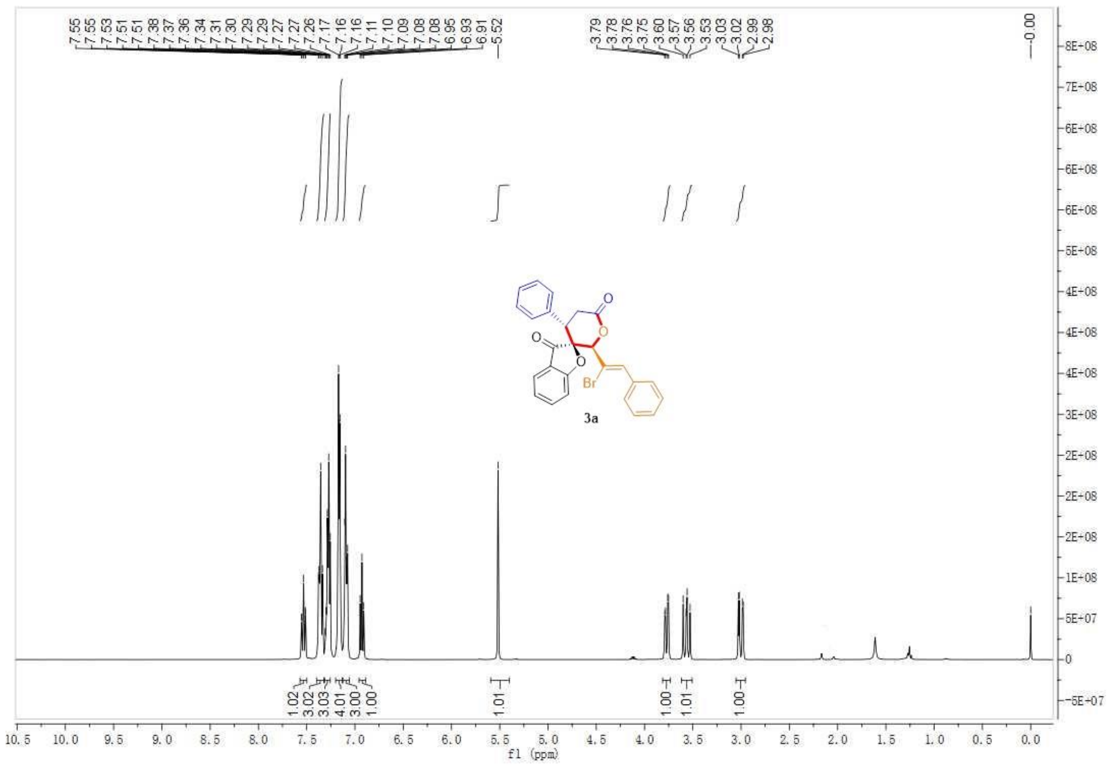

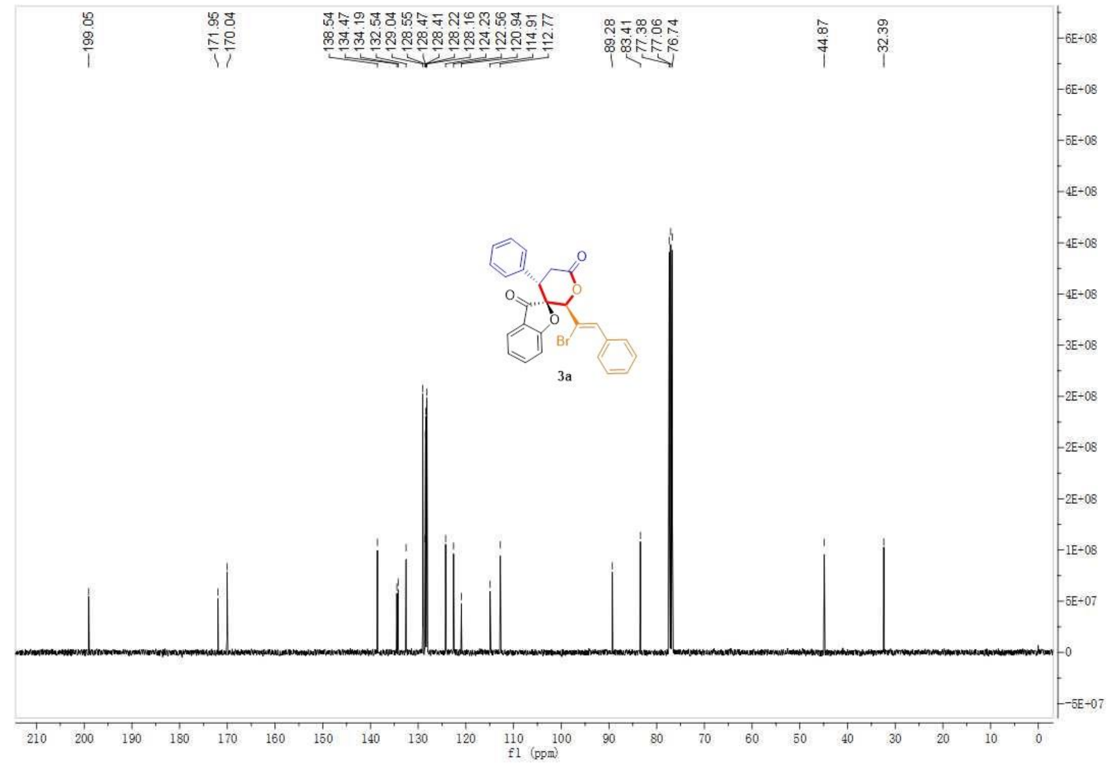

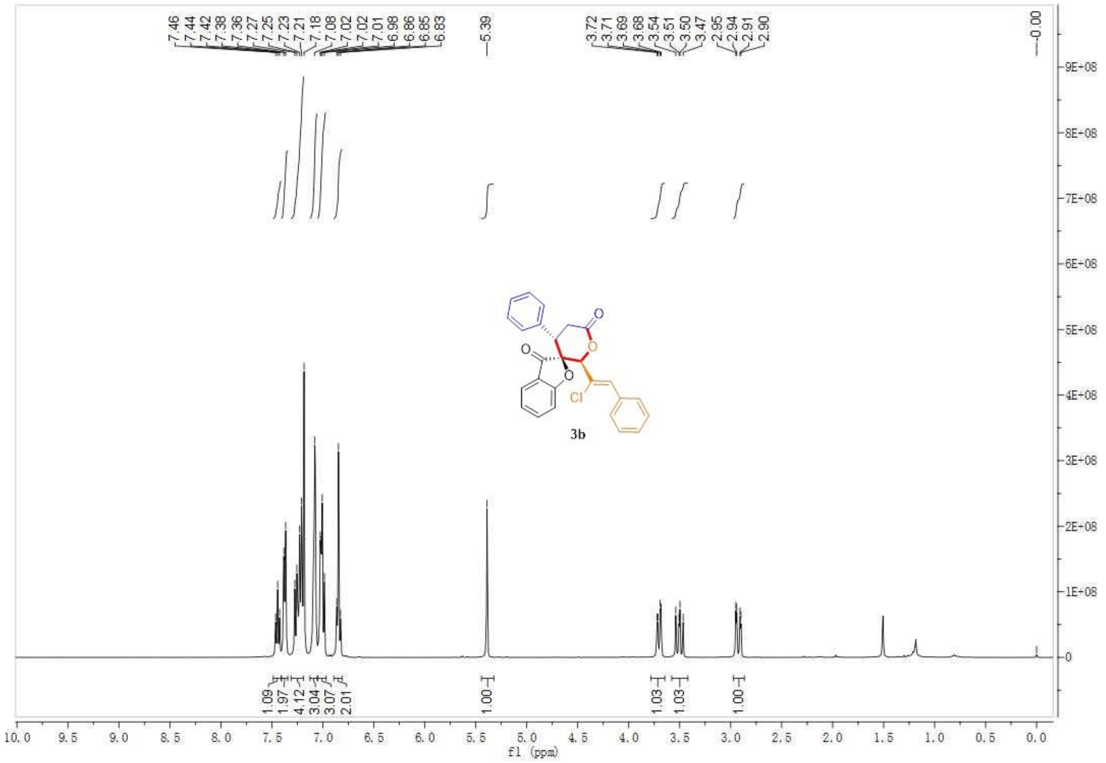

7.46 7.44 7.42 7.38 7.36 7.27 7.25 7.23 7.21 7.18 7.08 7.02 7.01 6.98 6.86 6.85 6.83
5.39
3.72 3.71 3.69 3.68 3.54 3.51 3.50 3.47 2.95 2.94 2.91 2.90
0.00
3b
1.09 1.97 4.12 3.04 3.07 2.01
1.00
1.03 1.03 1.00
f1 (ppm)

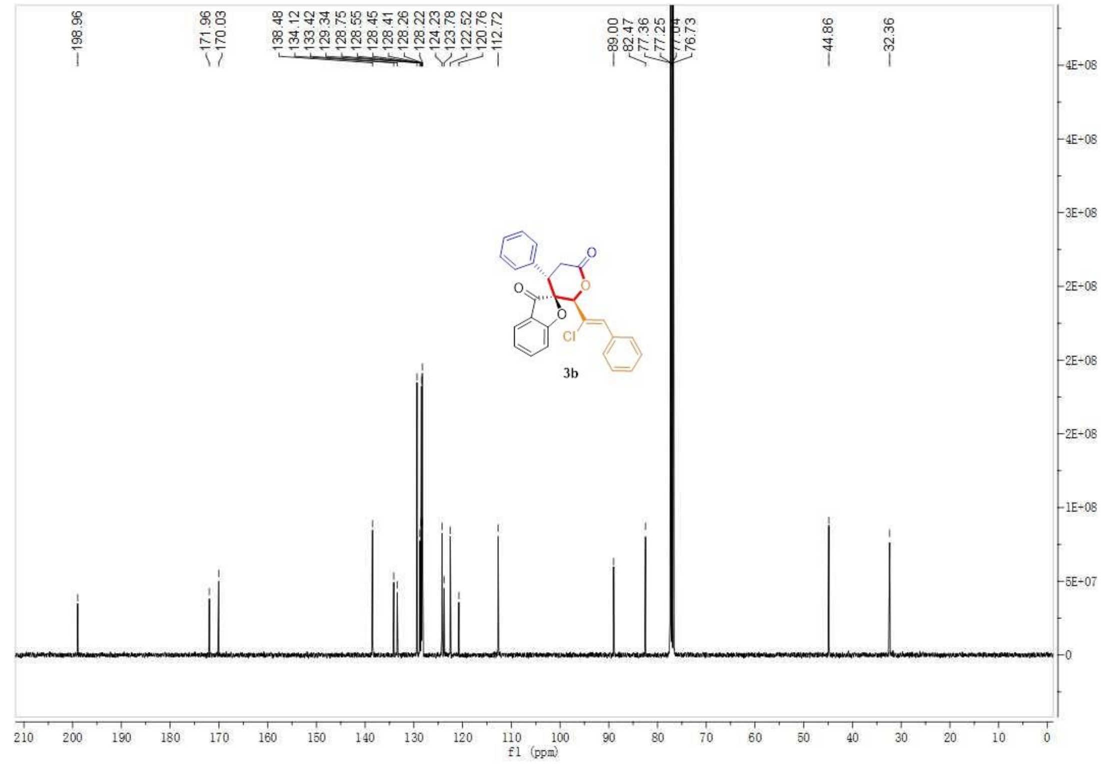

198.96
171.96 170.03
138.48 134.12 133.42 129.34 128.75 128.55 128.45 128.41 128.26 128.22 124.23 123.78 122.52 120.76 112.72
89.00 82.47 77.36 77.25 77.04 76.73
44.86
32.36
3b
f1 (ppm)

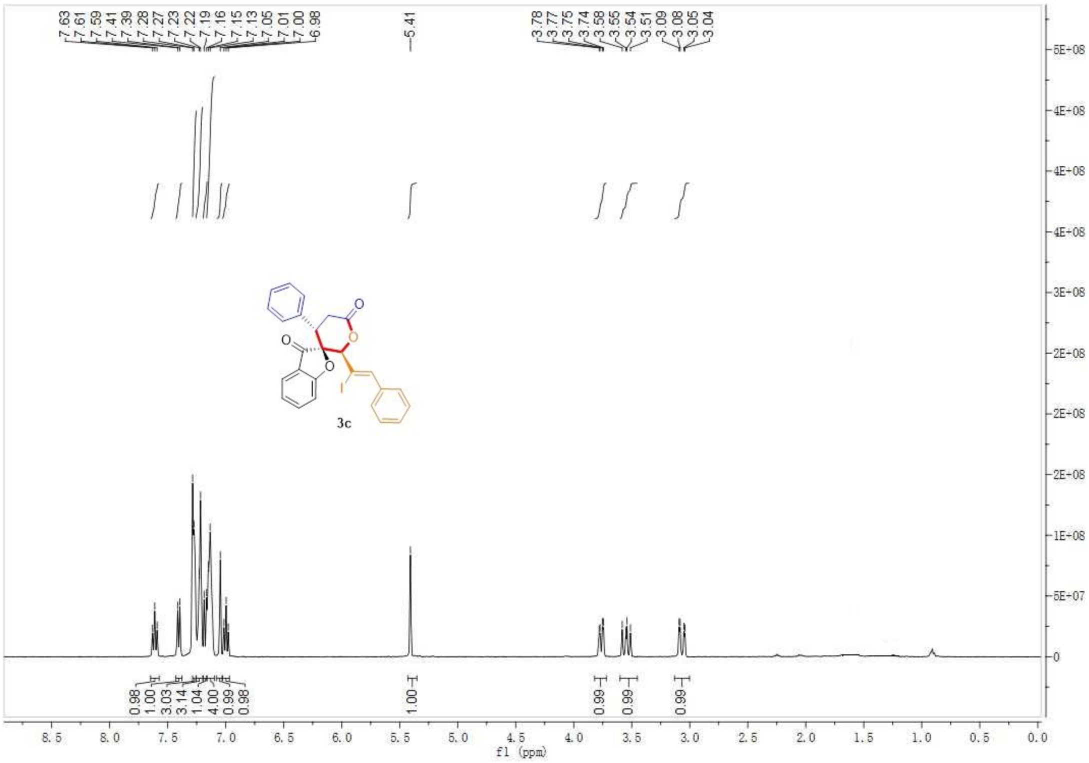
7.63
7.61
7.59
7.41
7.39
7.28
7.27
7.23
7.22
7.19
7.16
7.15
7.13
7.05
7.01
7.00
6.98
5.41
3.78
3.77
3.75
3.74
3.58
3.55
3.54
3.51
3.09
3.08
3.05
3.04
3c
0.98
1.00
3.03
3.14
1.04
4.00
0.99
0.98
1.00
0.99
0.99
0.99
f1 (ppm)

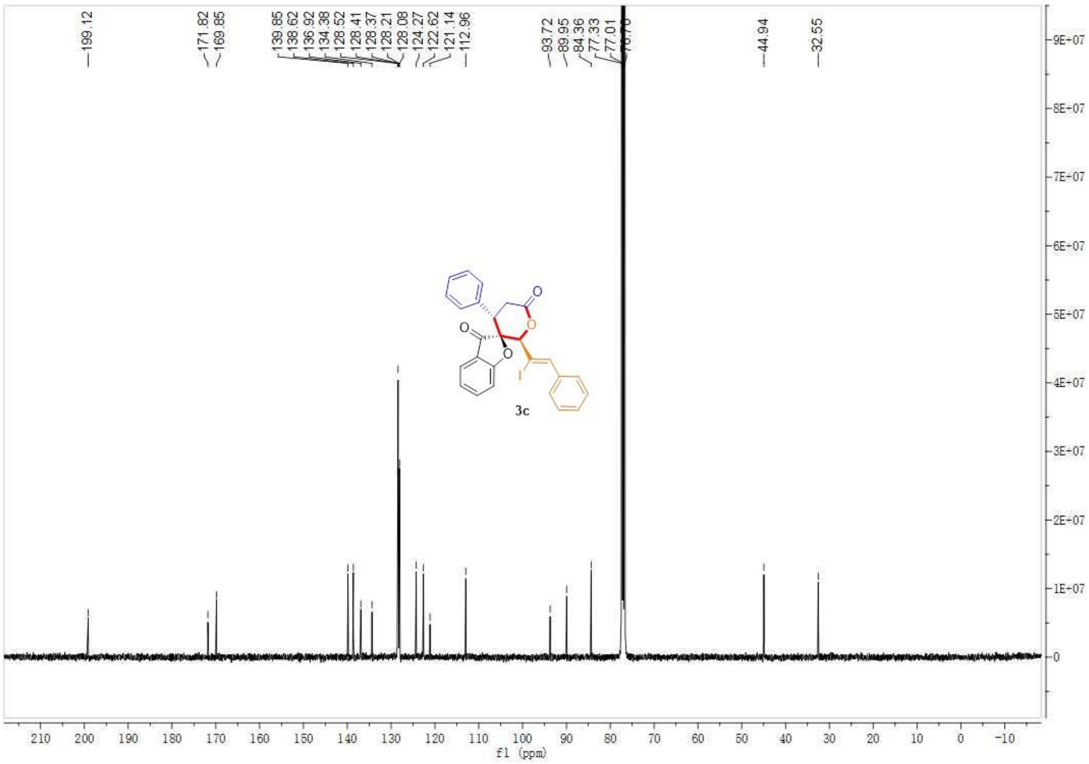
199.12
171.82
169.85
139.85
138.62
136.92
134.38
128.52
128.41
128.37
128.21
128.08
124.27
122.62
121.14
112.96
93.72
89.95
84.36
77.33
77.01
76.70
44.94
32.55
3c
f1 (ppm)

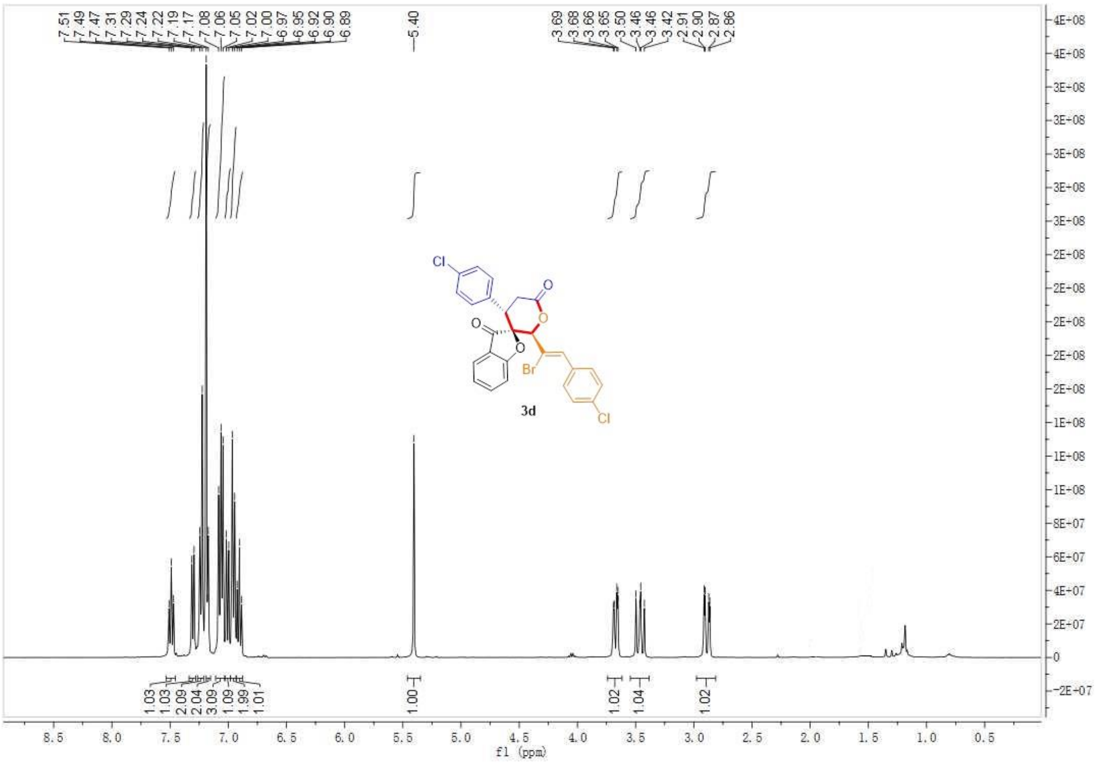
3d
f1 (ppm)

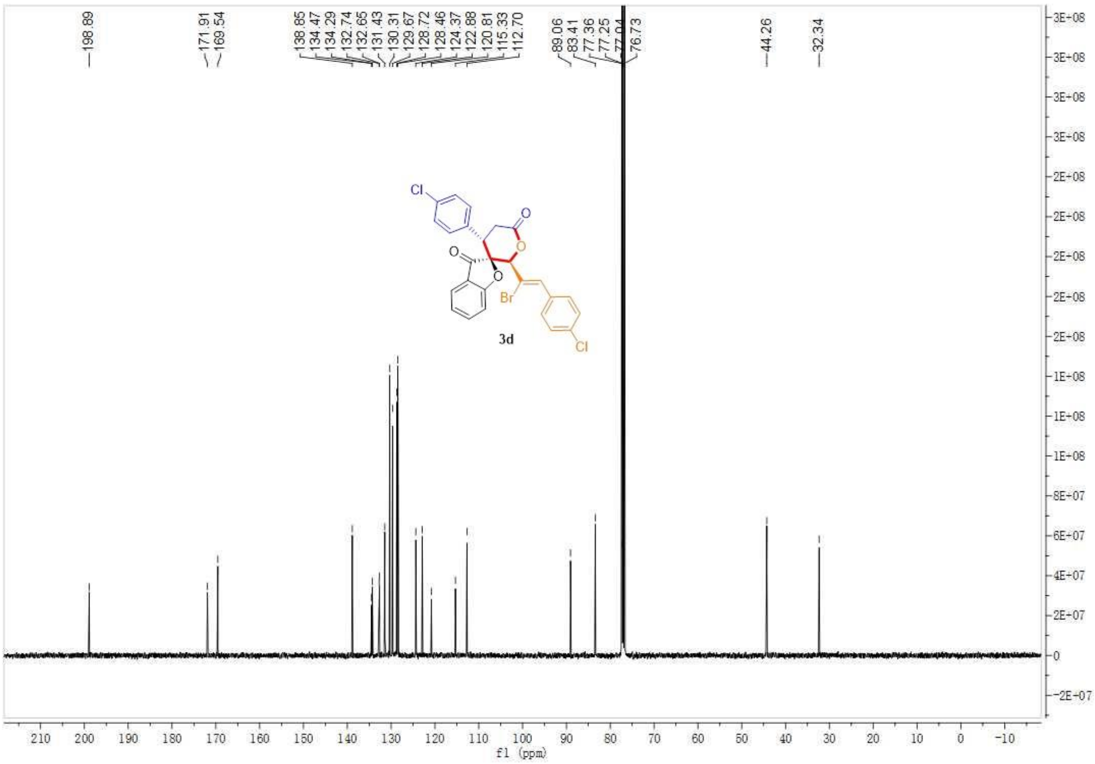
3d
f1 (ppm)

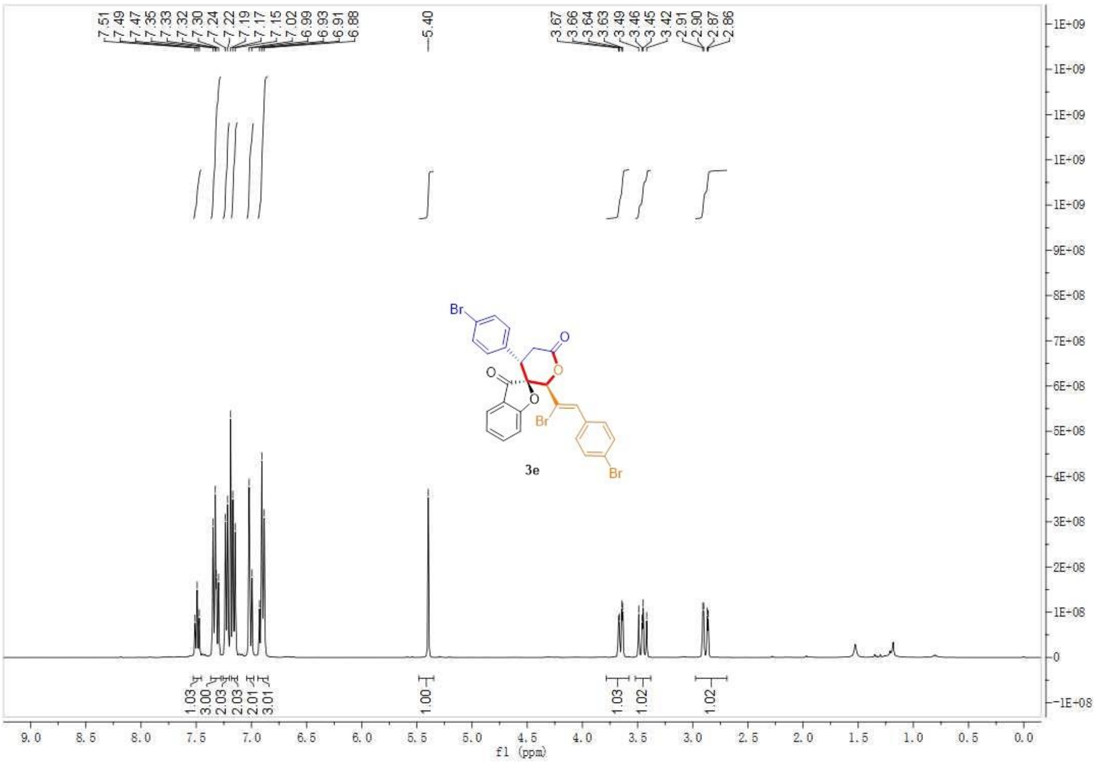
7.51 7.49 7.47 7.35 7.33 7.32 7.30 7.24 7.22 7.19 7.17 7.15 7.02 6.99 6.93 6.91 6.88
5.40
3.67 3.66 3.64 3.63 3.49 3.46 3.45 3.42 2.91 2.90 2.87 2.86
3e
1.03 3.00 2.03 2.03 2.01 3.01
1.00
1.03 1.02
1.02
f1 (ppm)

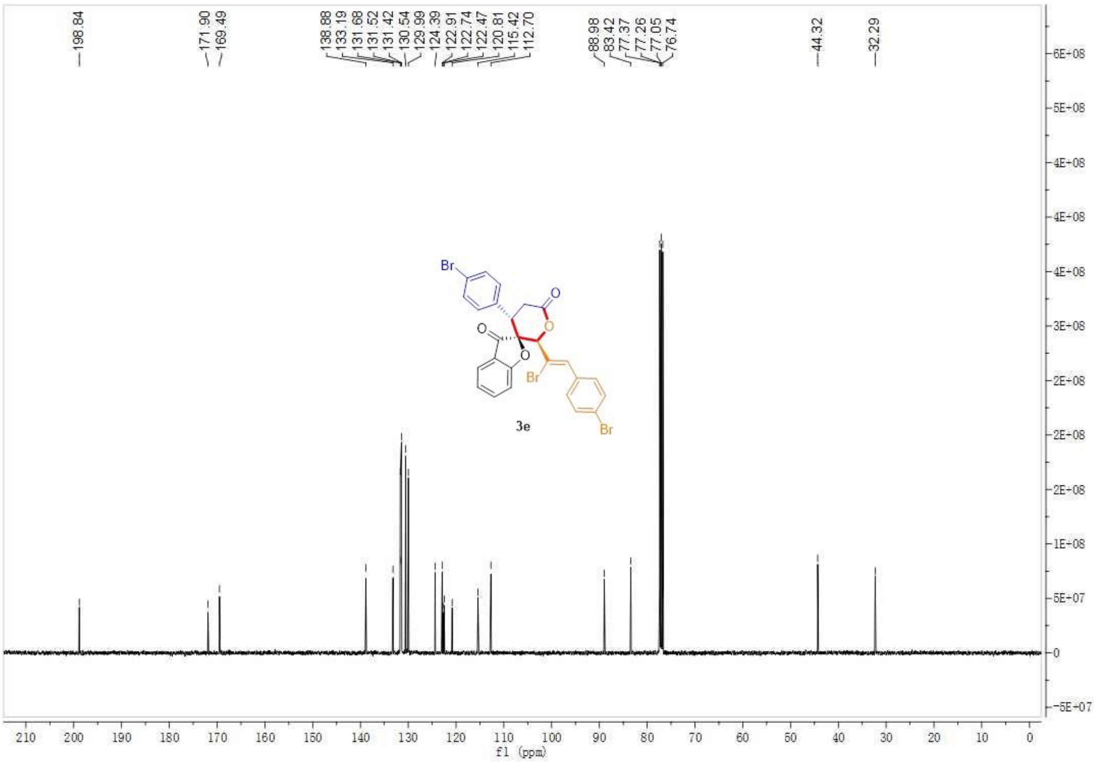
198.84
171.90 169.49
138.88 133.19 131.68 131.52 131.42 130.54 129.99 124.39 122.91 122.74 122.47 120.81 115.42 112.70
88.98 83.42 77.37 77.26 77.05 76.74
44.32
32.29
3e
f1 (ppm)

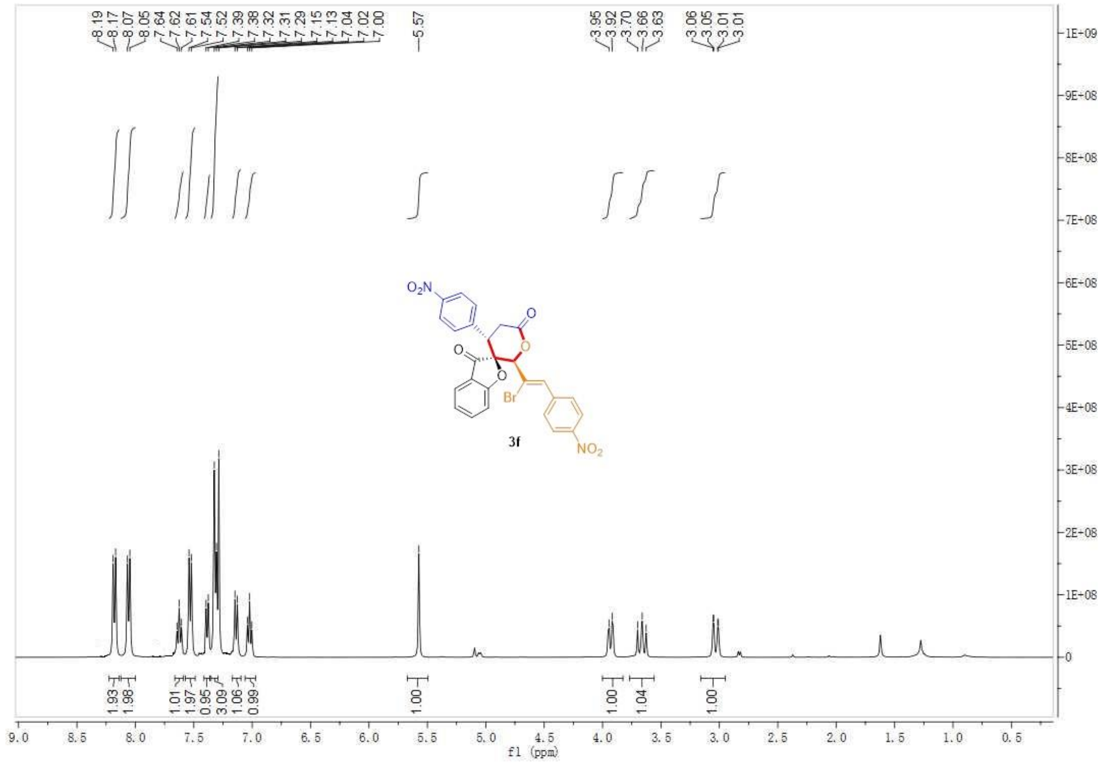
8.19
8.17
8.07
8.05
7.64
7.62
7.61
7.54
7.52
7.39
7.38
7.32
7.31
7.29
7.15
7.13
7.04
7.02
7.00
5.57
3.95
3.92
3.70
3.66
3.63
3.06
3.05
3.01
3.01
3f
1.93
1.98
1.01
1.97
0.95
3.09
1.06
0.99
1.00
1.00
1.04
1.00
f1 (ppm)

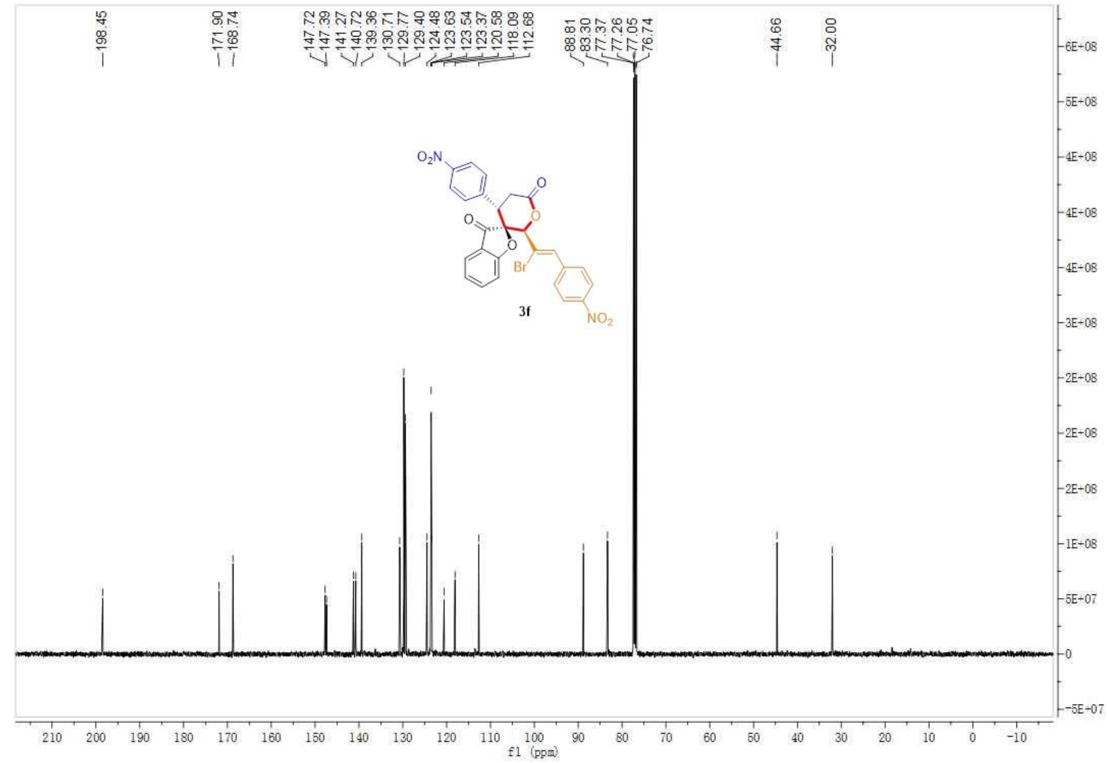
198.45
171.90
168.74
147.72
147.39
141.27
140.72
139.36
130.71
129.77
129.40
124.48
123.63
123.54
123.37
120.58
118.09
112.68
88.81
83.30
77.37
77.26
77.05
76.74
44.66
32.00
3f
f1 (ppm)

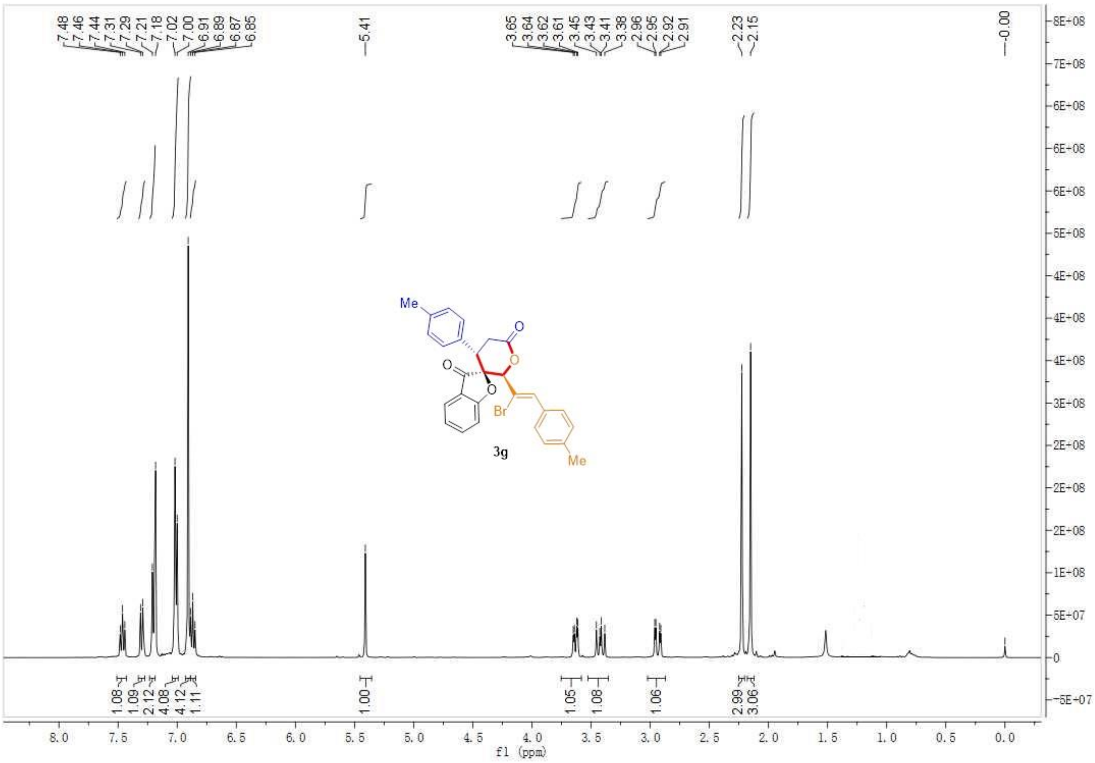
7.48
7.46
7.44
7.31
7.29
7.21
7.18
7.02
7.00
6.91
6.89
6.87
6.85
5.41
3.65
3.64
3.62
3.61
3.45
3.43
3.41
3.38
2.96
2.95
2.92
2.91
2.23
2.15
0.00
1.08
1.09
2.12
4.08
4.12
1.11
1.00
1.05
1.08
1.06
2.99
3.06
Me
O
O
O
O
Br
Me
3g
f1 (ppm)

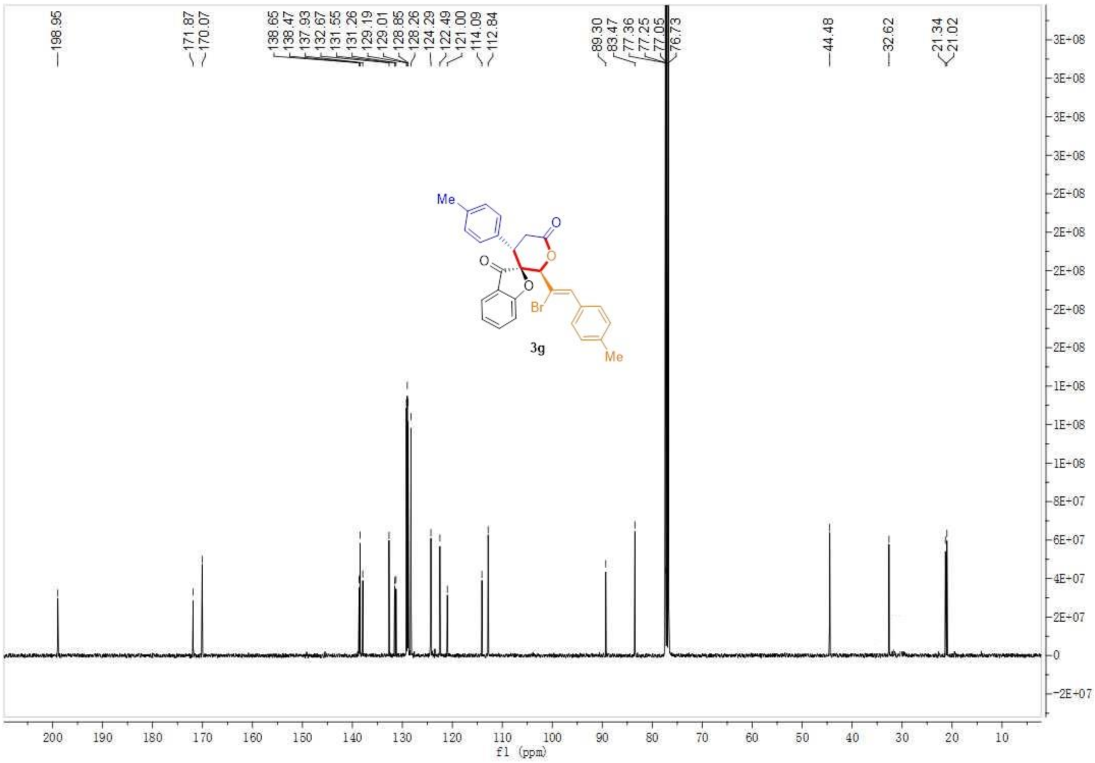
198.95
171.87
170.07
138.65
138.47
137.93
132.67
131.55
131.26
129.19
129.01
128.85
128.26
124.29
122.49
121.00
114.09
112.84
89.30
83.47
77.36
77.25
77.05
76.73
44.48
32.62
21.34
21.02
Me
O
O
O
O
Br
Me
3g
f1 (ppm)

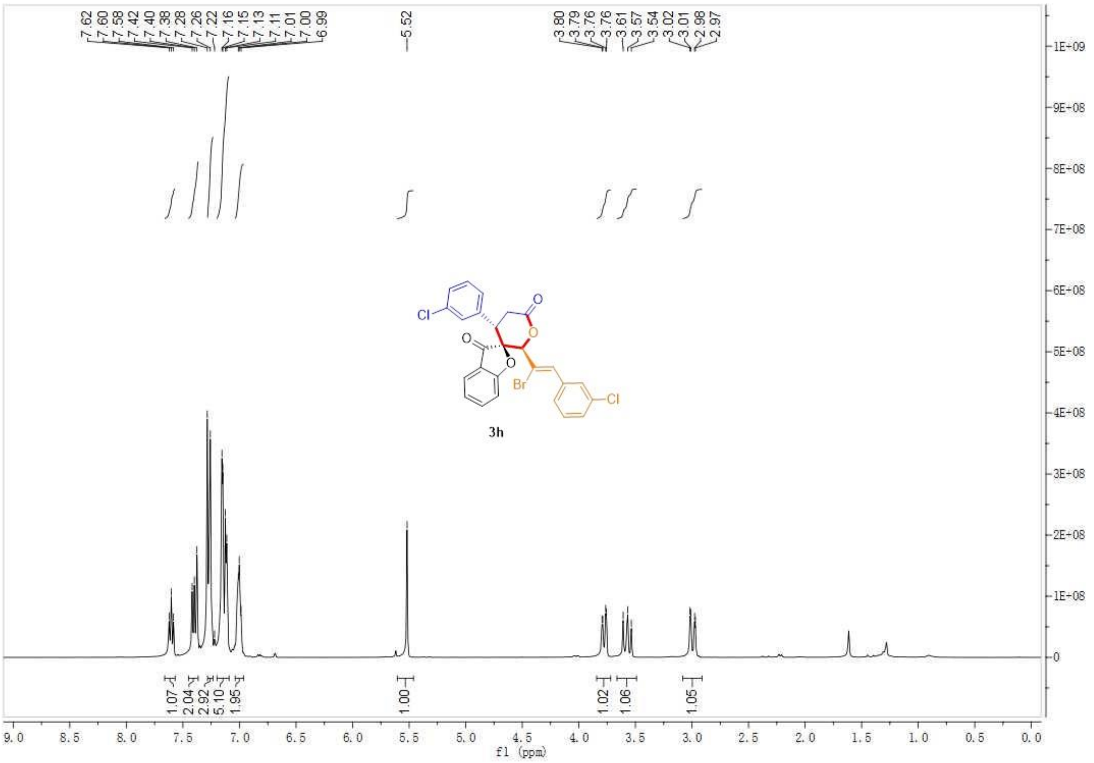
7.62
7.60
7.58
7.42
7.40
7.38
7.28
7.26
7.22
7.16
7.15
7.13
7.11
7.01
7.00
6.99
5.52
3.80
3.79
3.76
3.76
3.61
3.57
3.54
3.02
3.01
2.98
2.97
Cl
O
O
O
O
Br
Cl
3h
1.07
2.04
2.92
5.10
1.95
1.00
1.02
1.06
1.05
f1 (ppm)

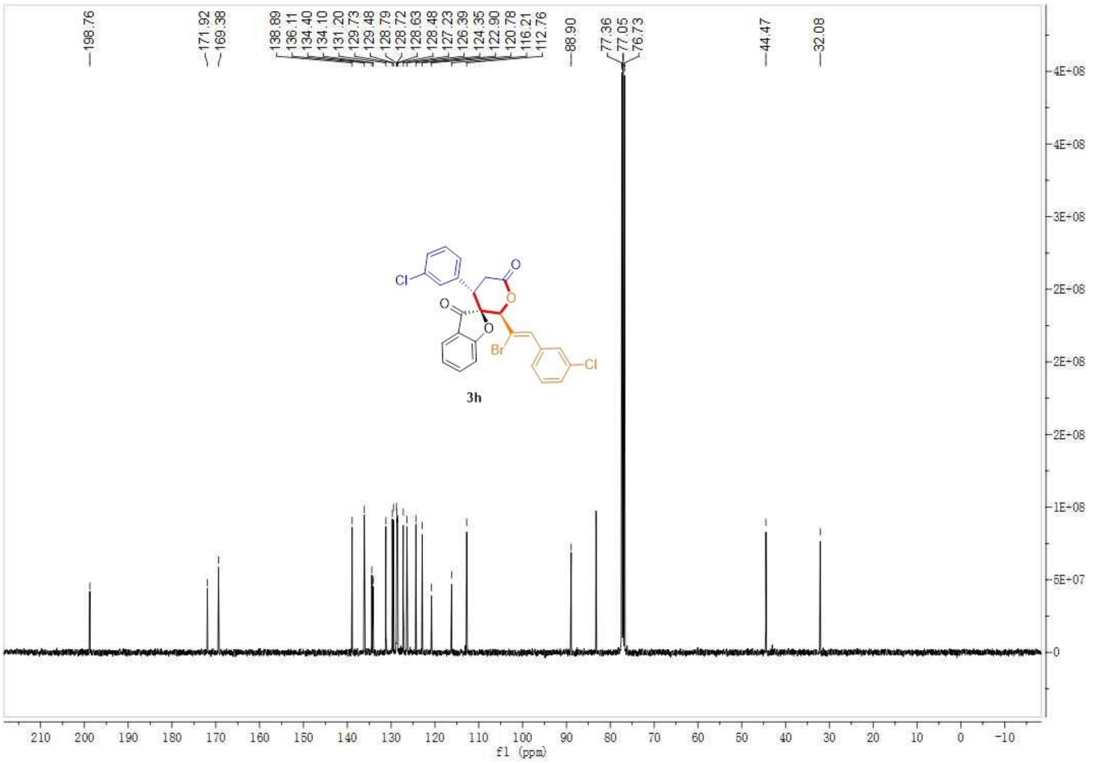
198.76
171.92
169.38
138.89
136.11
134.40
134.10
131.20
129.73
129.48
128.79
128.72
128.63
128.48
127.23
126.39
124.35
122.90
120.78
116.21
112.76
88.90
77.36
77.05
76.73
44.47
32.08
Cl
O
O
O
O
Br
Cl
3h
f1 (ppm)

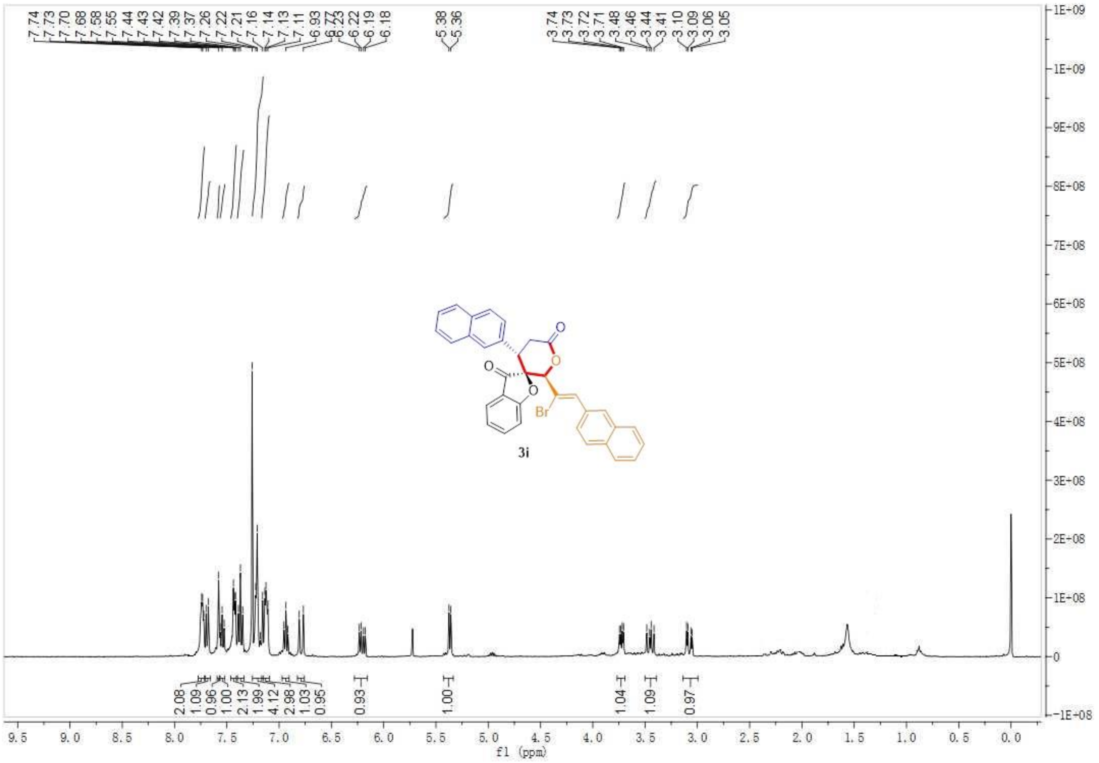
7.74 7.73 7.70 7.68 7.58 7.55 7.44 7.43 7.42 7.39 7.37 7.26 7.22 7.21 7.16 7.14 7.13 7.11 6.93 6.77 6.23 6.22 6.19 6.18
5.38 5.36
3.74 3.73 3.72 3.71 3.48 3.46 3.44 3.41 3.10 3.09 3.06 3.05
2.08 1.09 0.96 1.00 2.13 1.99 4.12 2.98 1.03 0.95
0.93
1.00
1.04
1.09
0.97
O
O
O
O
Br
3i
f1 (ppm)

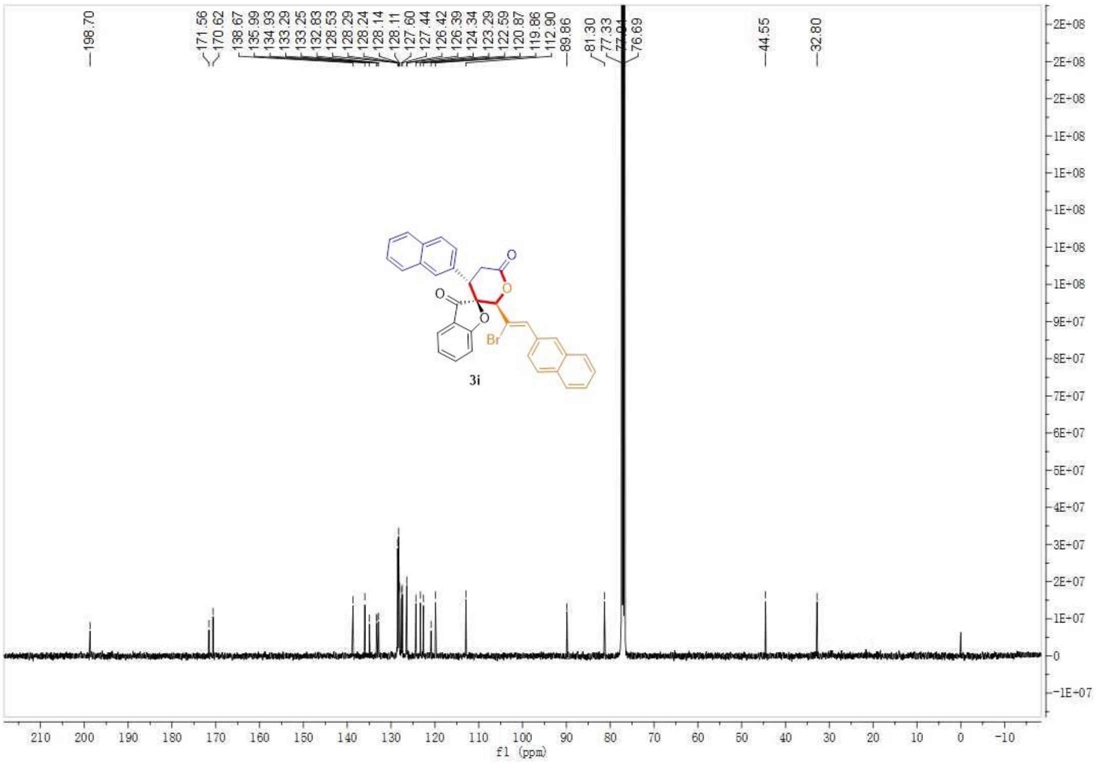
198.70
171.56 170.62 138.67 135.99 134.93 133.29 133.25 132.83 128.53 128.29 128.24 128.14 128.11 127.60 127.44 126.42 126.39 124.34 123.29 122.59 120.87 119.86 112.90 89.86
81.30 77.33 77.01 76.69
44.55
32.80
O
O
O
O
Br
3i
f1 (ppm)

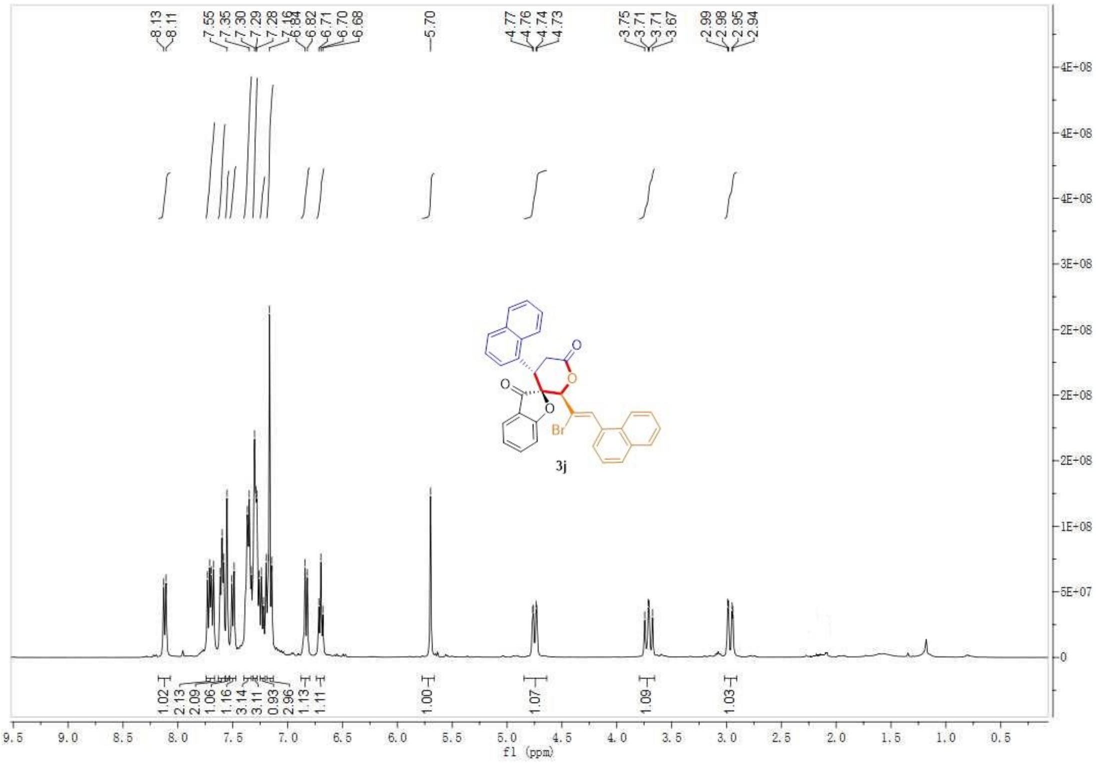
8.13
8.11
7.55
7.35
7.30
7.29
7.28
7.16
6.84
6.82
6.71
6.70
6.68
5.70
4.77
4.76
4.74
4.73
3.75
3.71
3.71
3.67
2.99
2.98
2.95
2.94
3j
Br
O
1.02
2.13
2.09
1.06
1.16
3.14
3.11
0.93
2.96
1.13
1.11
1.00
1.07
1.09
1.03
f1 (ppm)

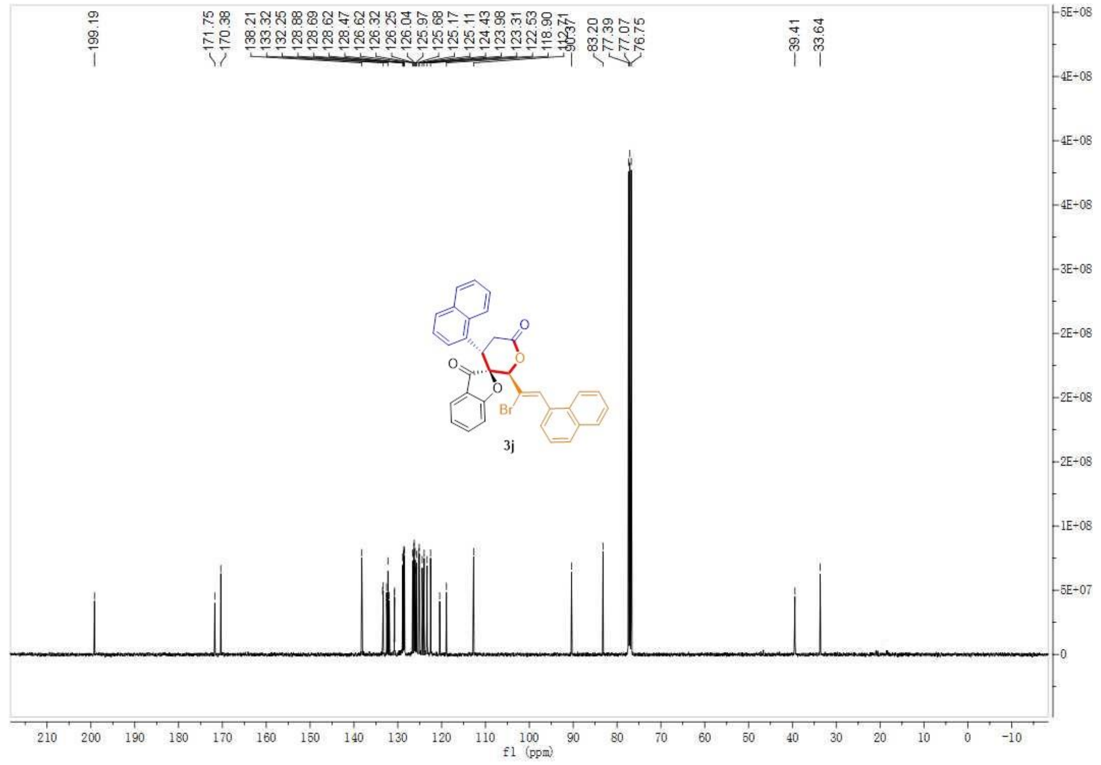
199.19
171.75
170.38
138.21
133.32
132.25
128.88
128.69
128.62
128.47
126.62
126.32
126.25
126.04
125.97
125.68
125.17
125.11
124.43
123.98
123.31
122.53
118.90
112.71
90.37
83.20
77.39
77.07
76.75
39.41
33.64
3j
Br
O
f1 (ppm)

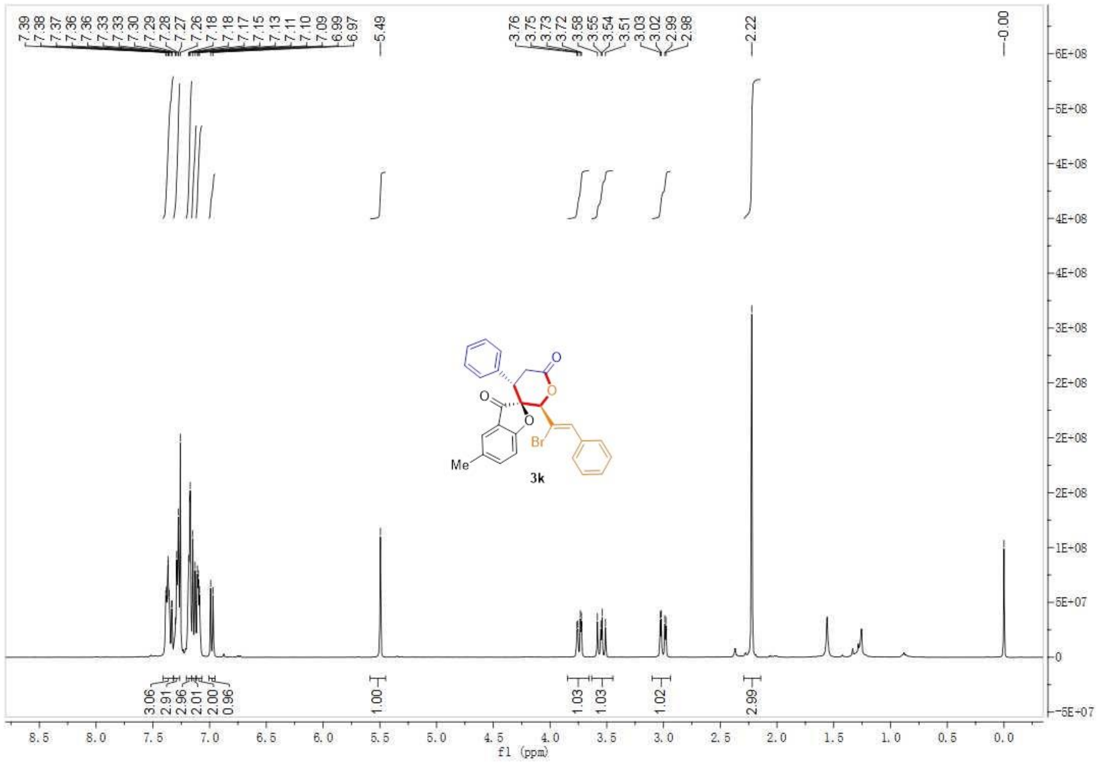
3k
Me
Br
O
fl (ppm)

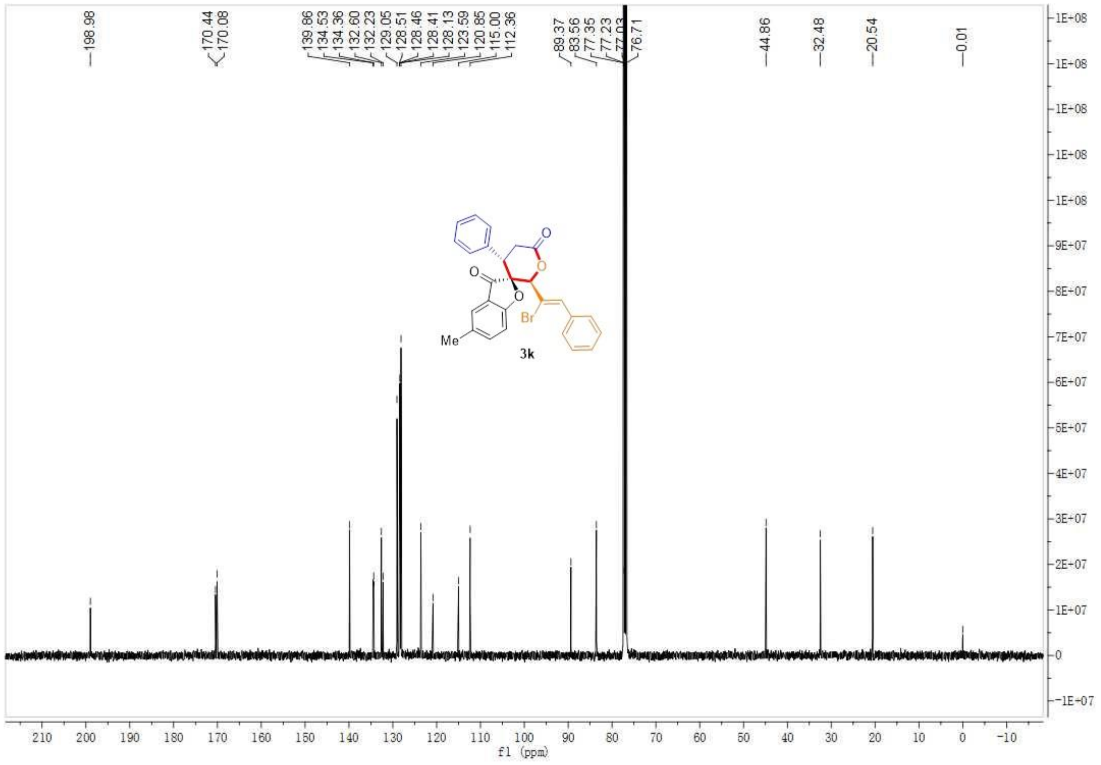
3k
Me
Br
O
fl (ppm)

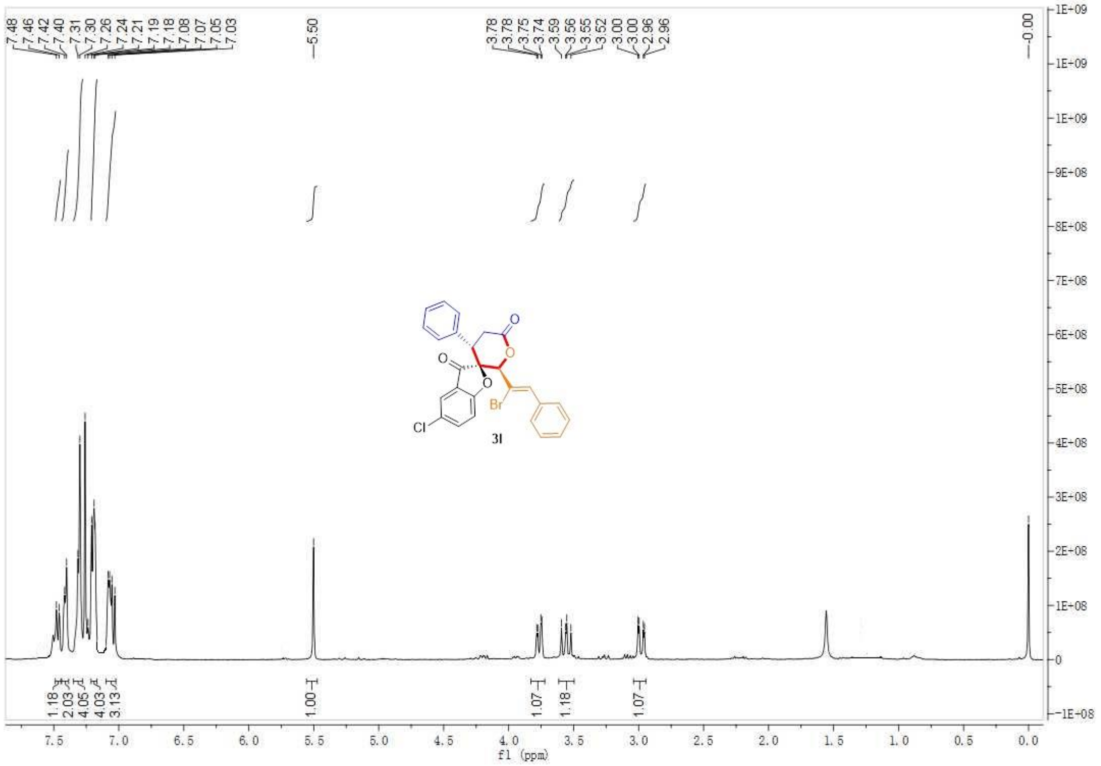

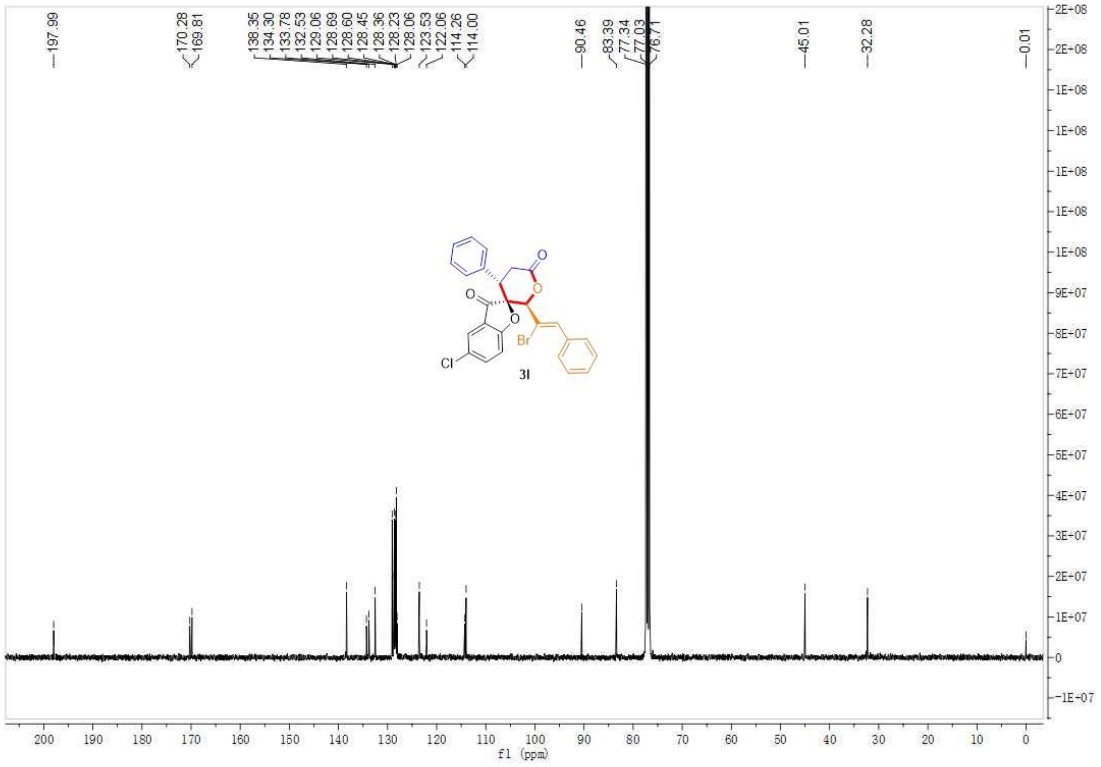

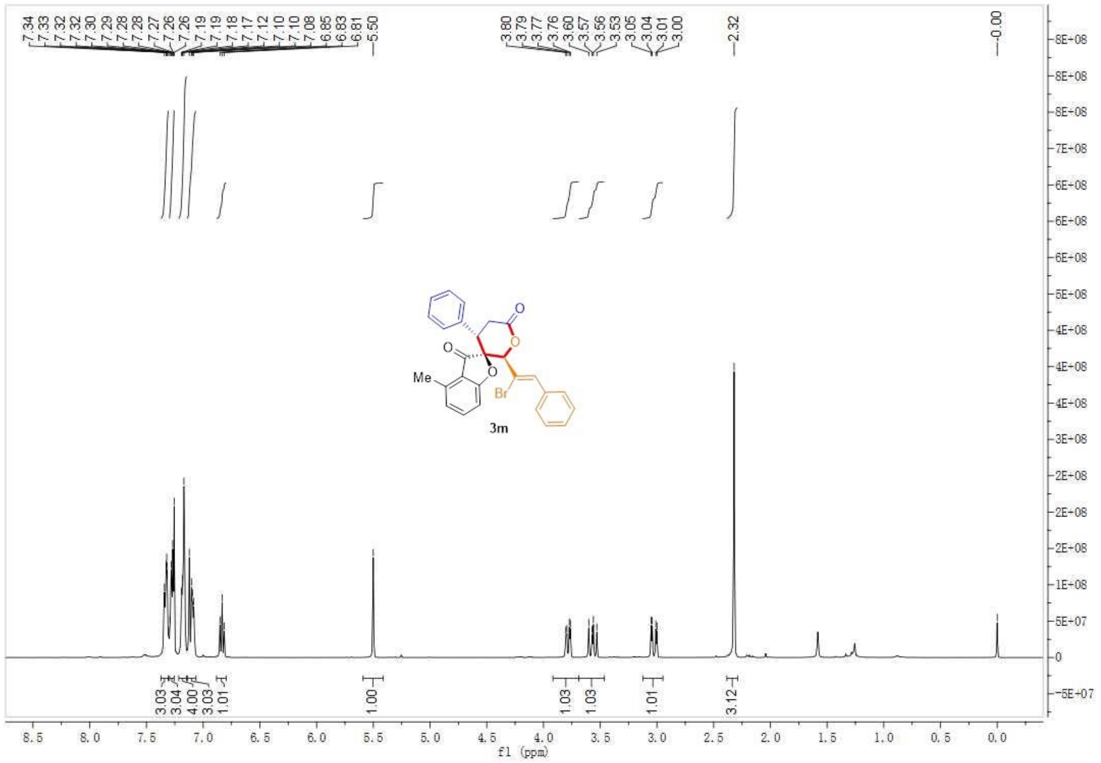

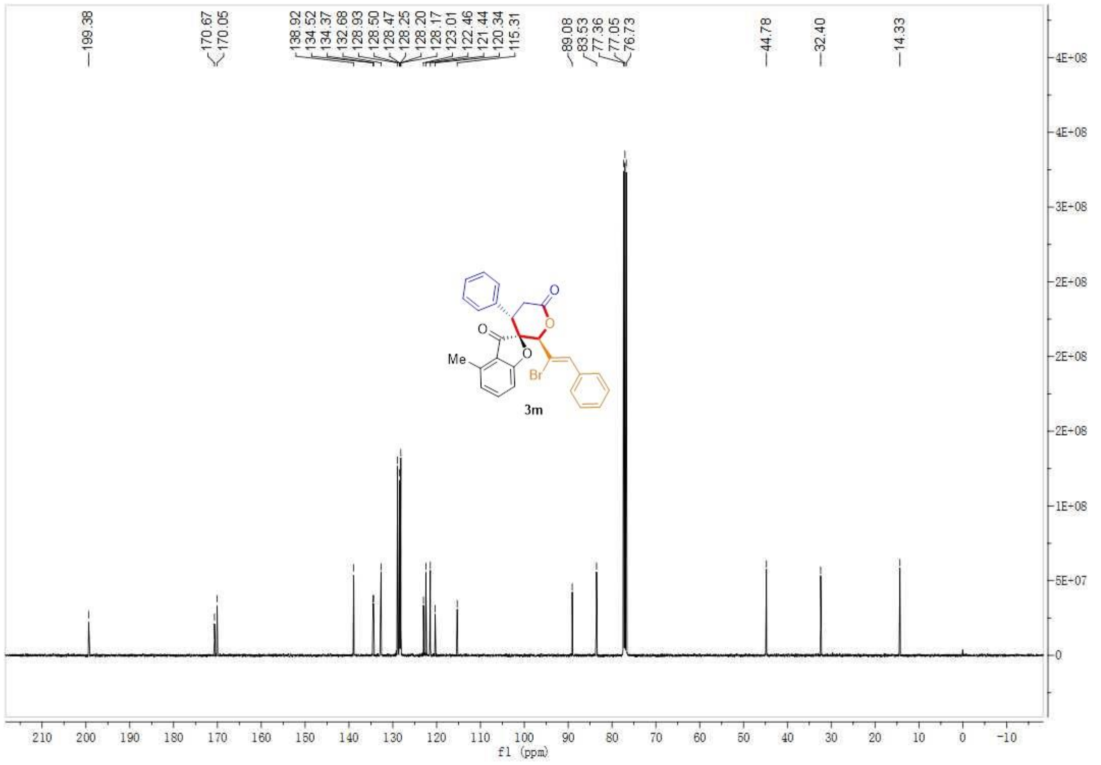

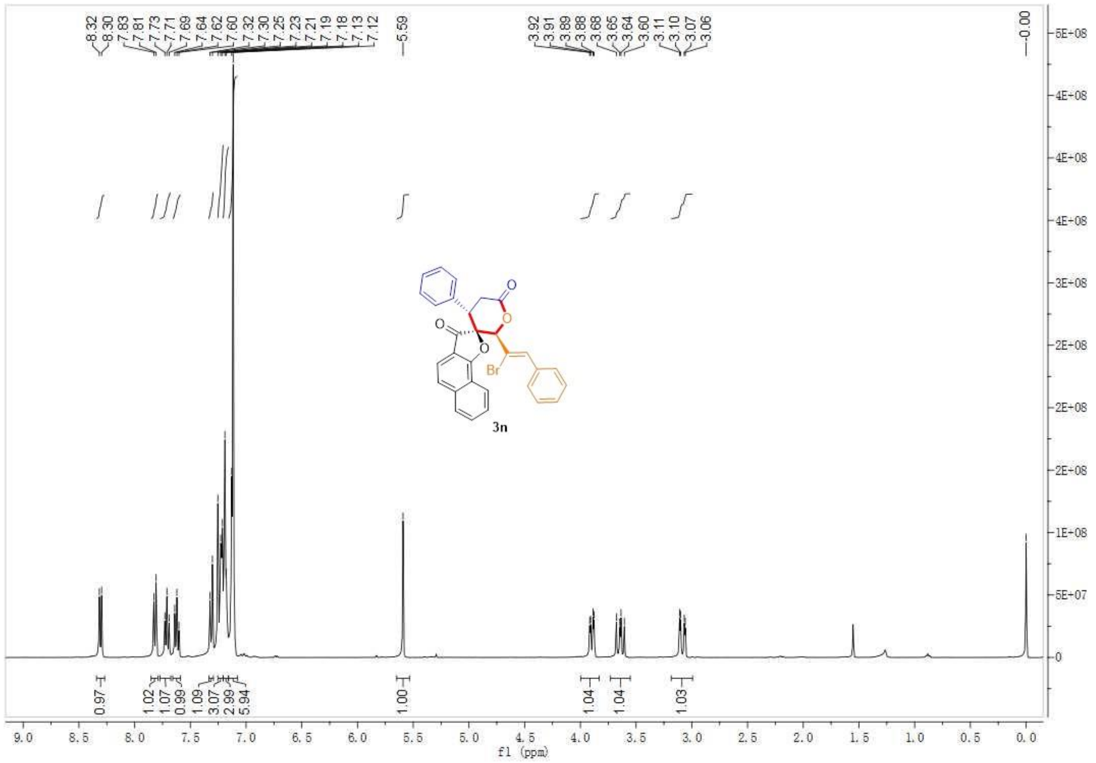
8.32
8.30
7.83
7.81
7.73
7.71
7.69
7.64
7.62
7.60
7.32
7.30
7.25
7.23
7.21
7.19
7.18
7.13
7.12
5.59
3.92
3.91
3.89
3.88
3.68
3.65
3.64
3.60
3.11
3.10
3.07
3.06
0.00
O
O
O
O
Br
3n
0.97
1.02
1.07
0.99
1.09
3.07
2.99
5.94
1.00
1.04
1.04
1.03
f1 (ppm)

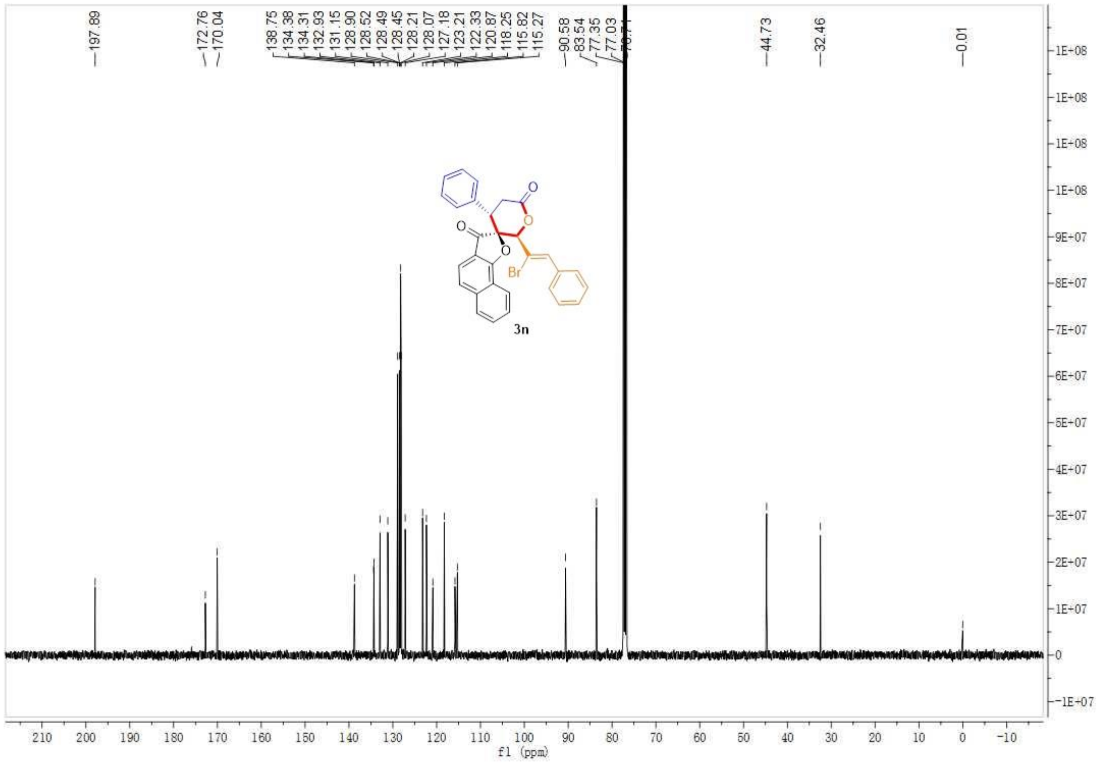
197.89
172.76
170.04
138.75
134.38
134.31
132.93
131.15
128.90
128.52
128.49
128.45
128.21
128.07
127.18
123.21
122.33
120.87
118.25
115.82
115.27
90.58
83.54
77.35
77.03
44.73
32.46
-0.01
O
O
O
O
Br
3n
f1 (ppm)

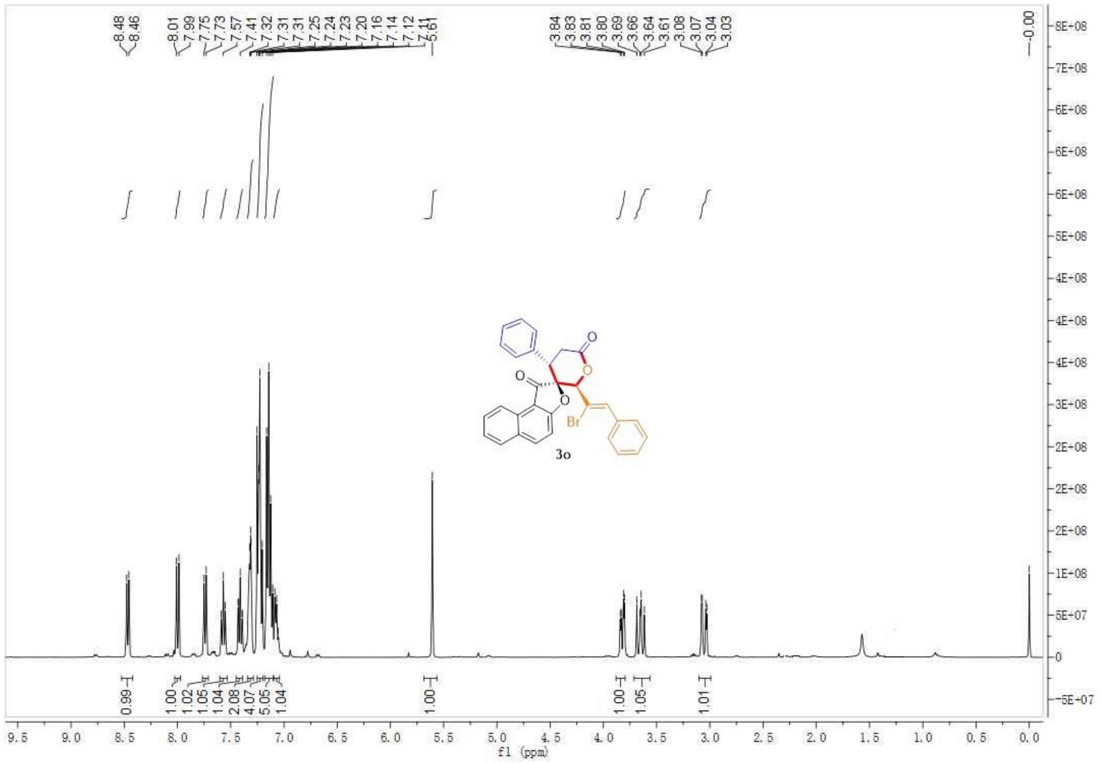

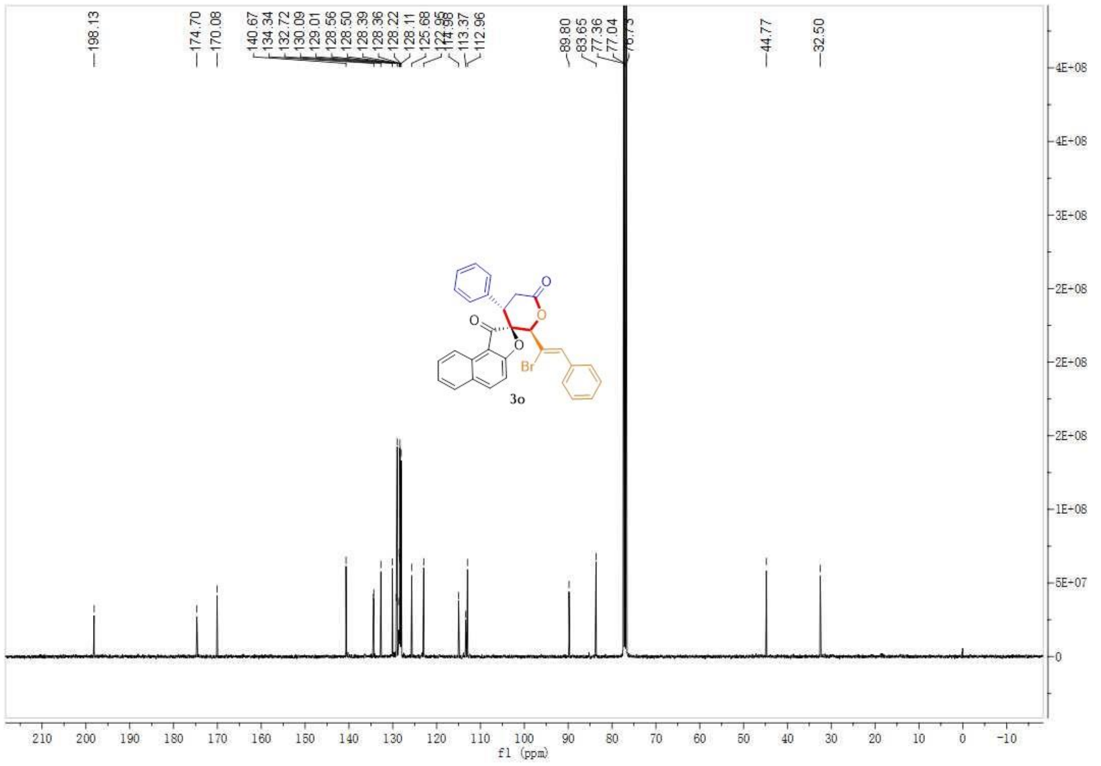

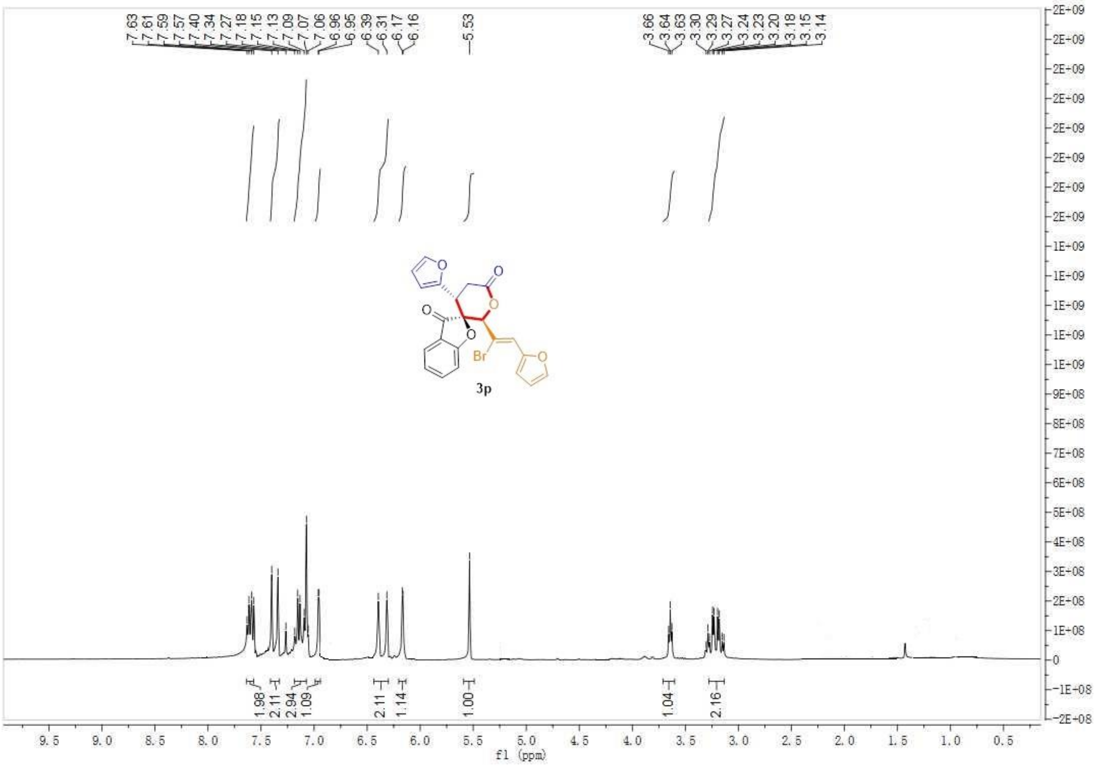
7.63 7.61 7.59 7.57 7.40 7.34 7.27 7.18 7.15 7.13 7.09 7.07 7.06 6.96 6.95 6.39 6.31 6.17 6.16
5.53
3.66 3.64 3.63 3.30 3.29 3.27 3.24 3.23 3.20 3.18 3.15 3.14
Br
3p
1.98 2.11 2.94 1.09 2.11 1.14 1.00 1.04 2.16
f1 (ppm)

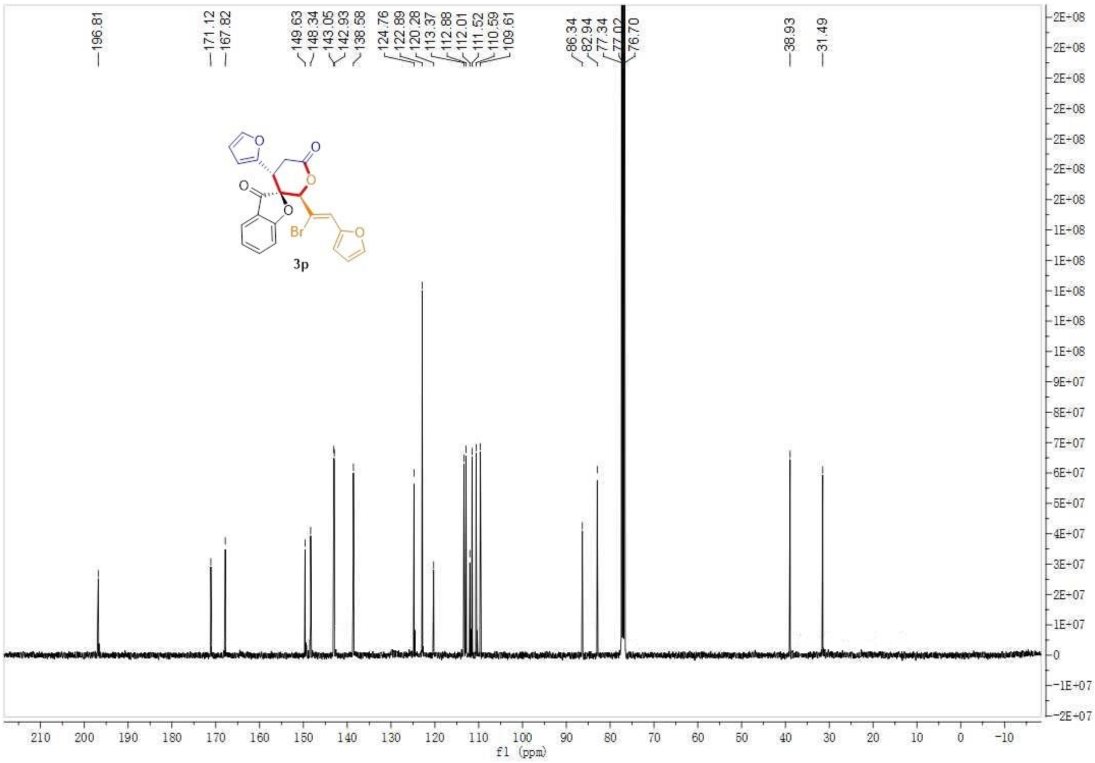
196.81
171.12 167.82
149.63 148.34 143.05 142.93 138.58
124.76 122.89 120.28 113.37 112.88 112.01 111.52 110.59 109.61
86.34 82.94 77.34 77.02 76.70
38.93 31.49
Br
3p
f1 (ppm)

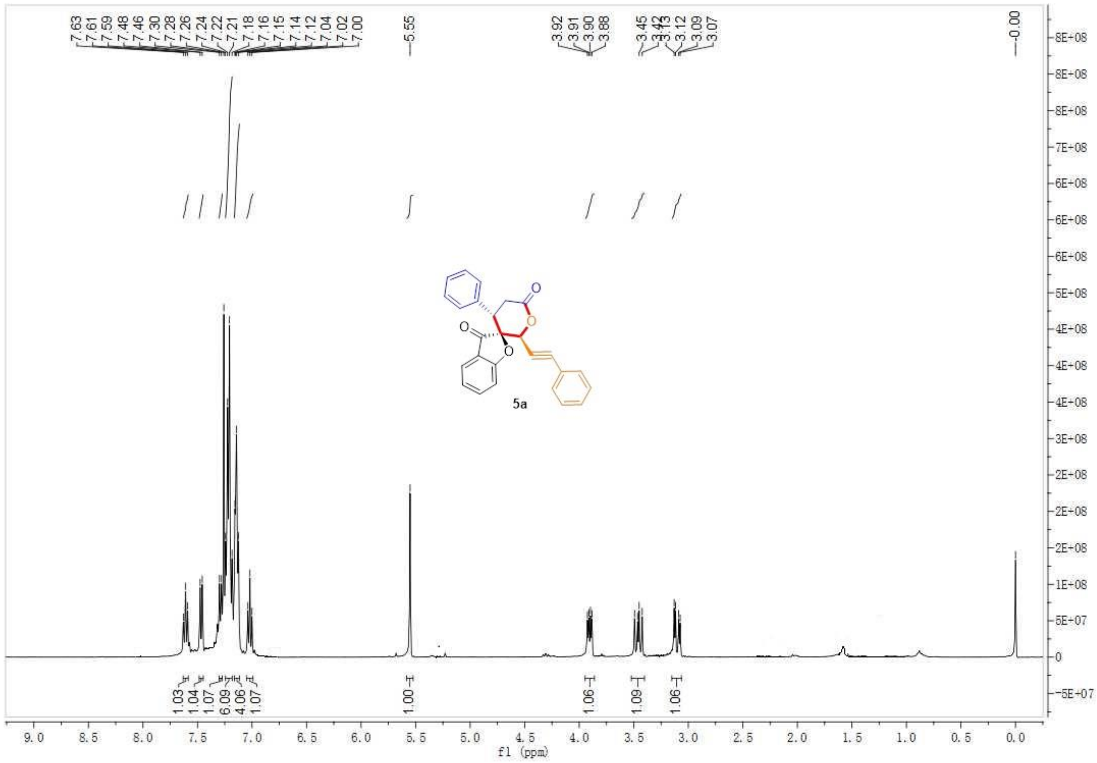

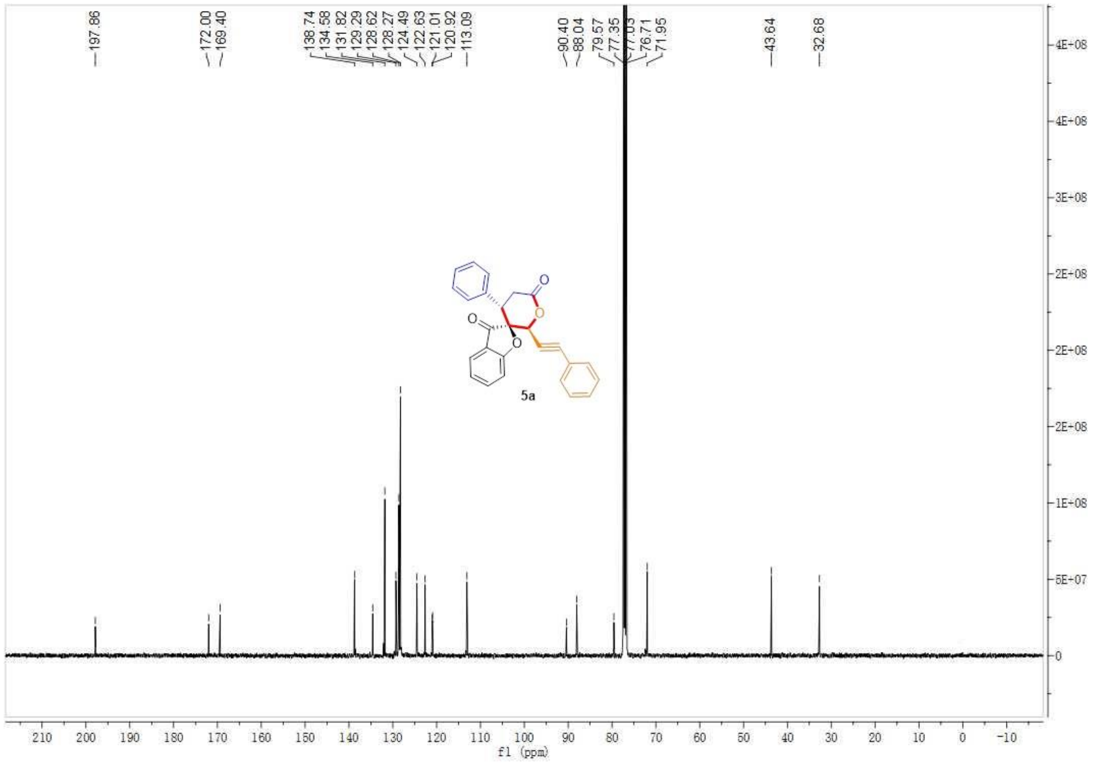

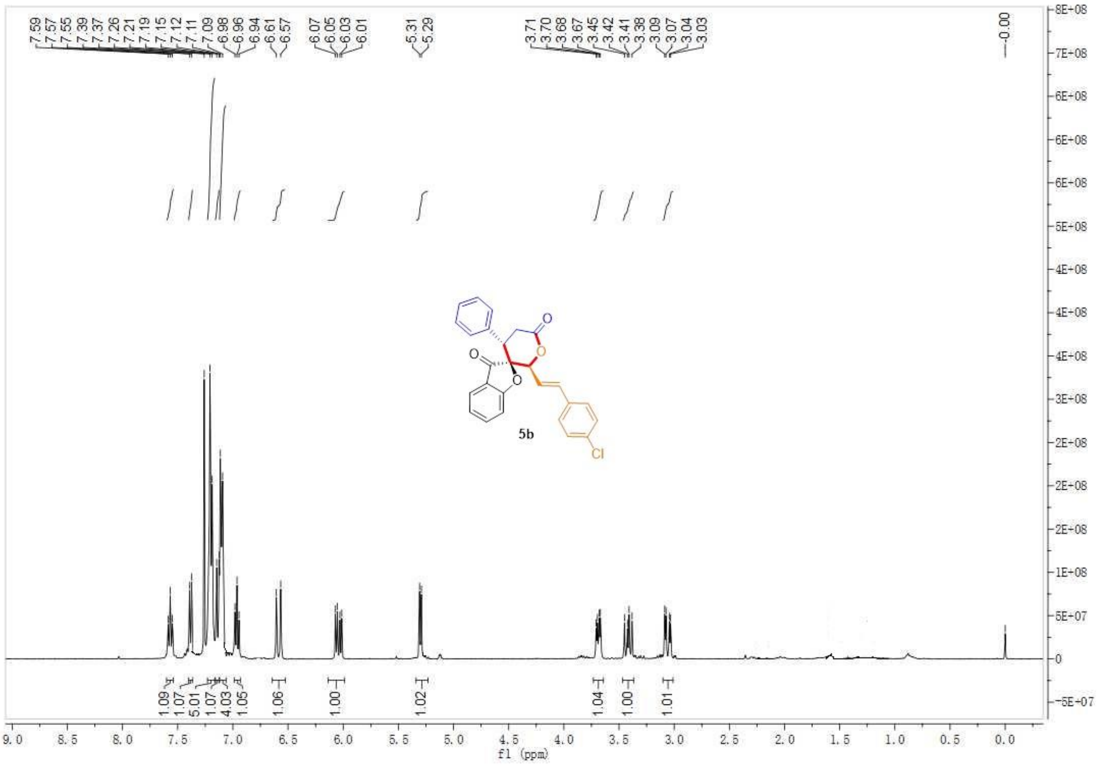

7.59 7.57 7.55 7.39 7.37 7.26 7.21 7.19 7.15 7.12 7.11 7.09 6.98 6.96 6.94 6.61 6.57
6.07 6.05 6.03 6.01
5.31 5.29
3.71 3.70 3.68 3.67 3.45 3.42 3.41 3.38 3.09 3.07 3.04 3.03
0.00
1.09 1.07 5.01 1.07 4.03 1.05 1.06 1.00 1.02 1.04 1.00 1.01
5b
Cl
f1 (ppm)

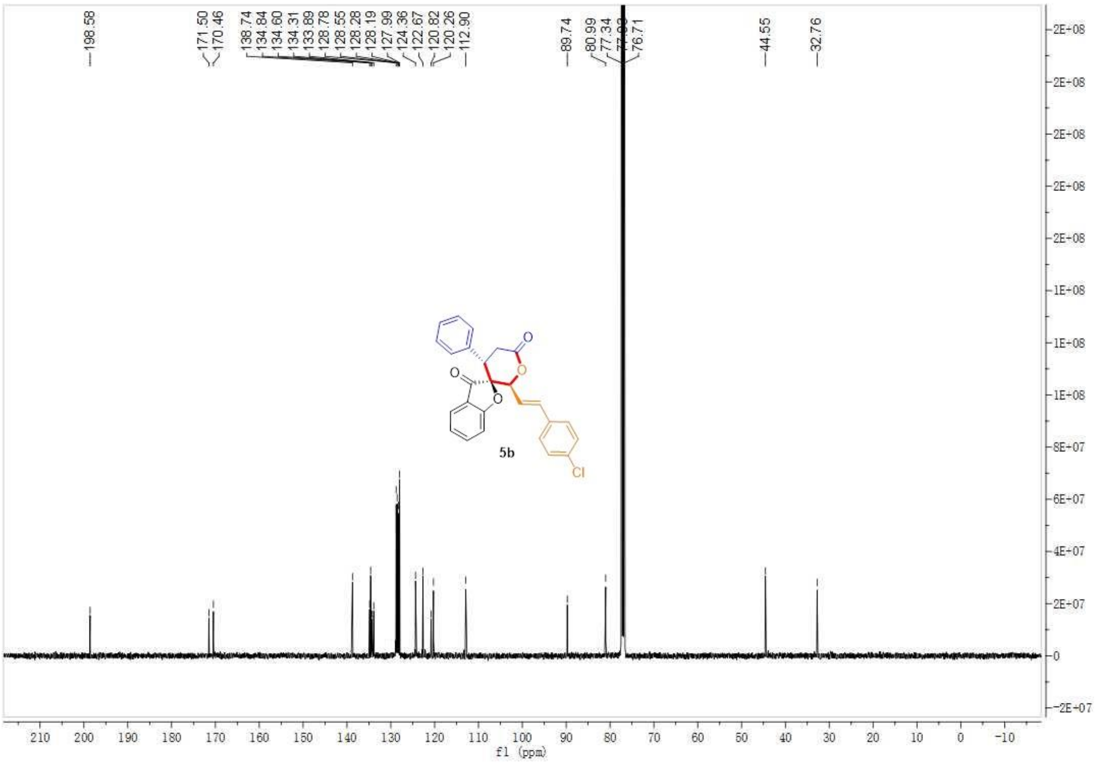

198.58
171.50 170.46
138.74 134.84 134.60 134.31 133.89 128.78 128.55 128.28 128.19 127.99 124.36 122.67 120.82 120.26 112.90
89.74
80.99 77.34 76.71
44.55
32.76
5b
Cl
f1 (ppm)

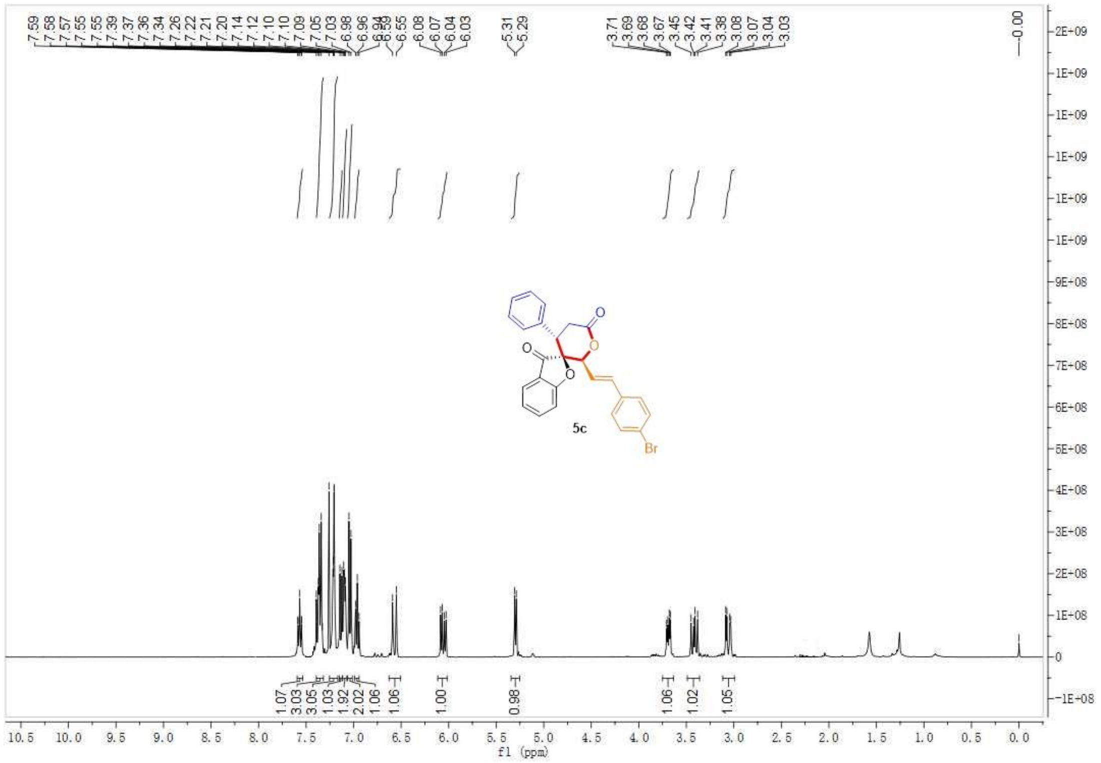
5c
Br
f1 (ppm)

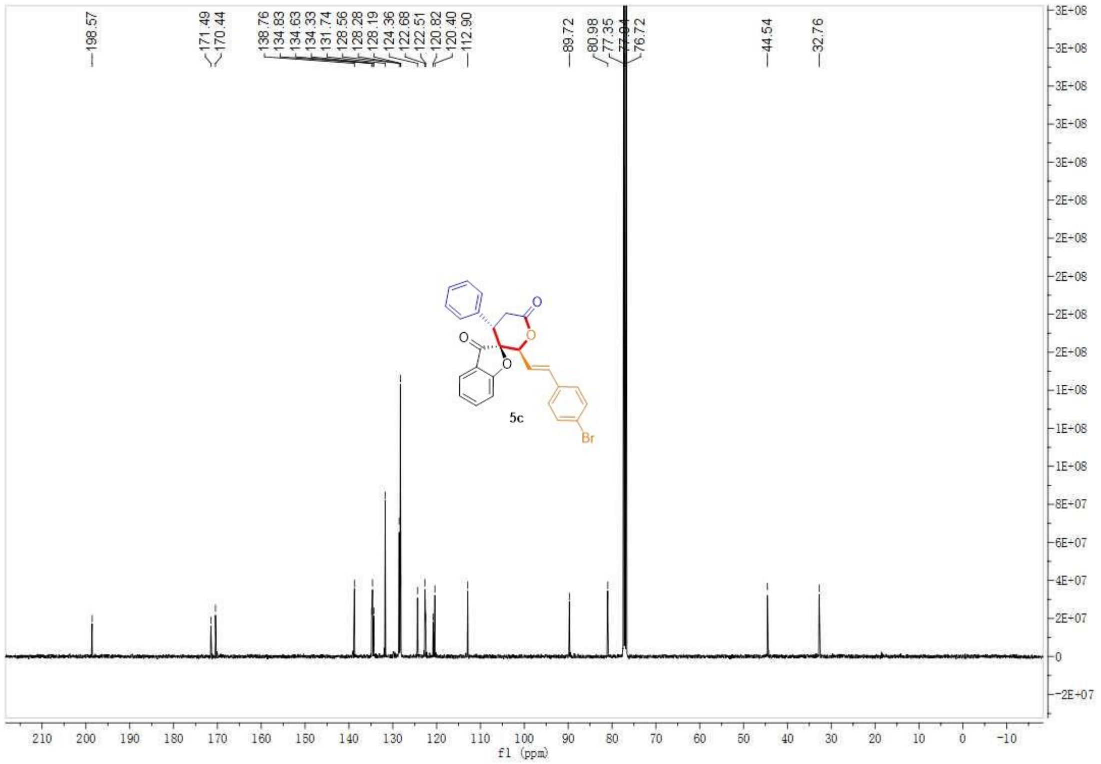
5c
Br
f1 (ppm)

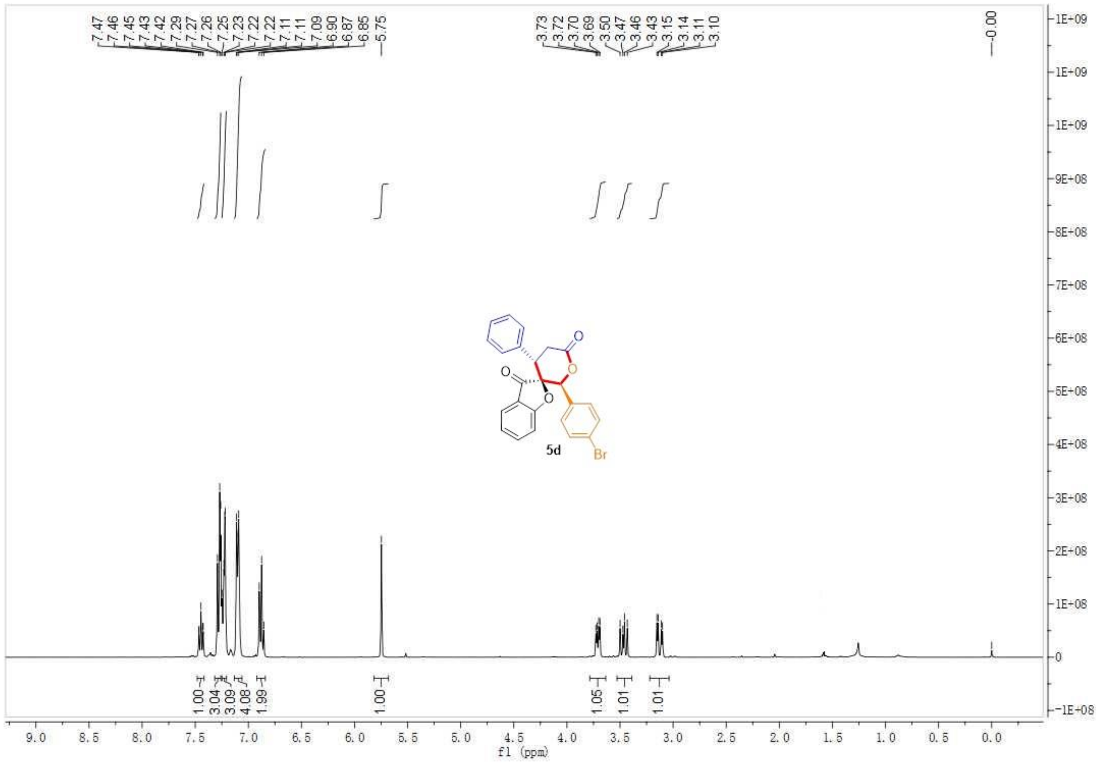

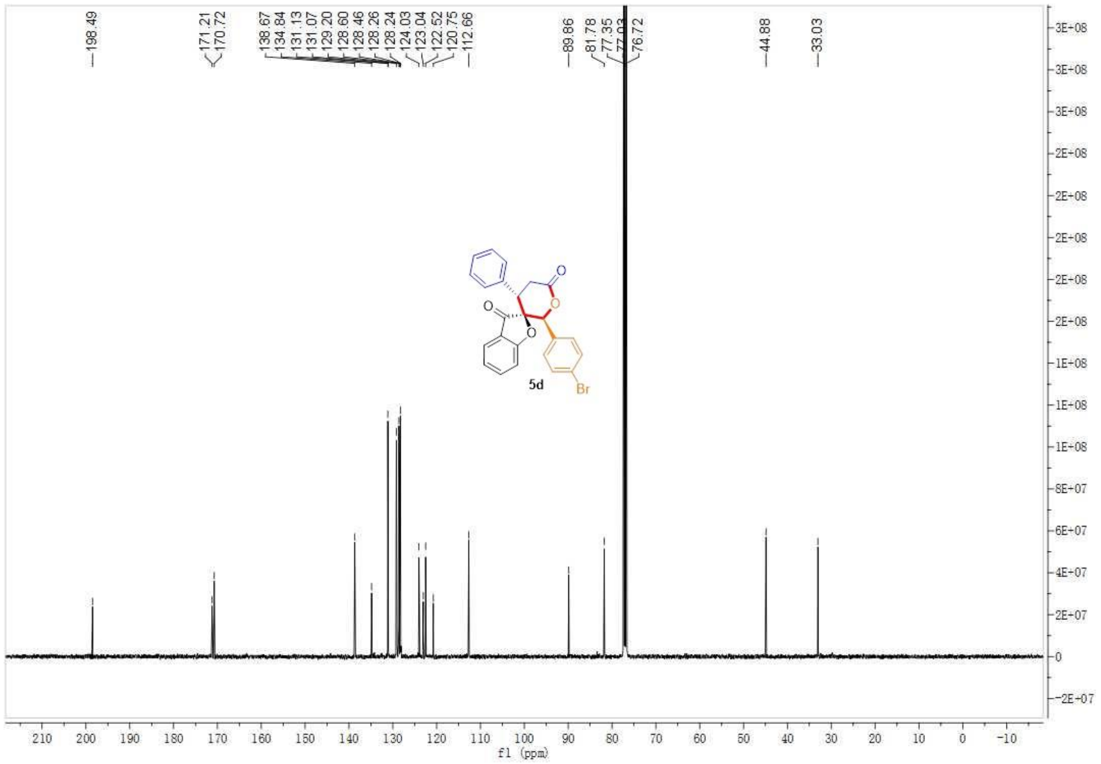

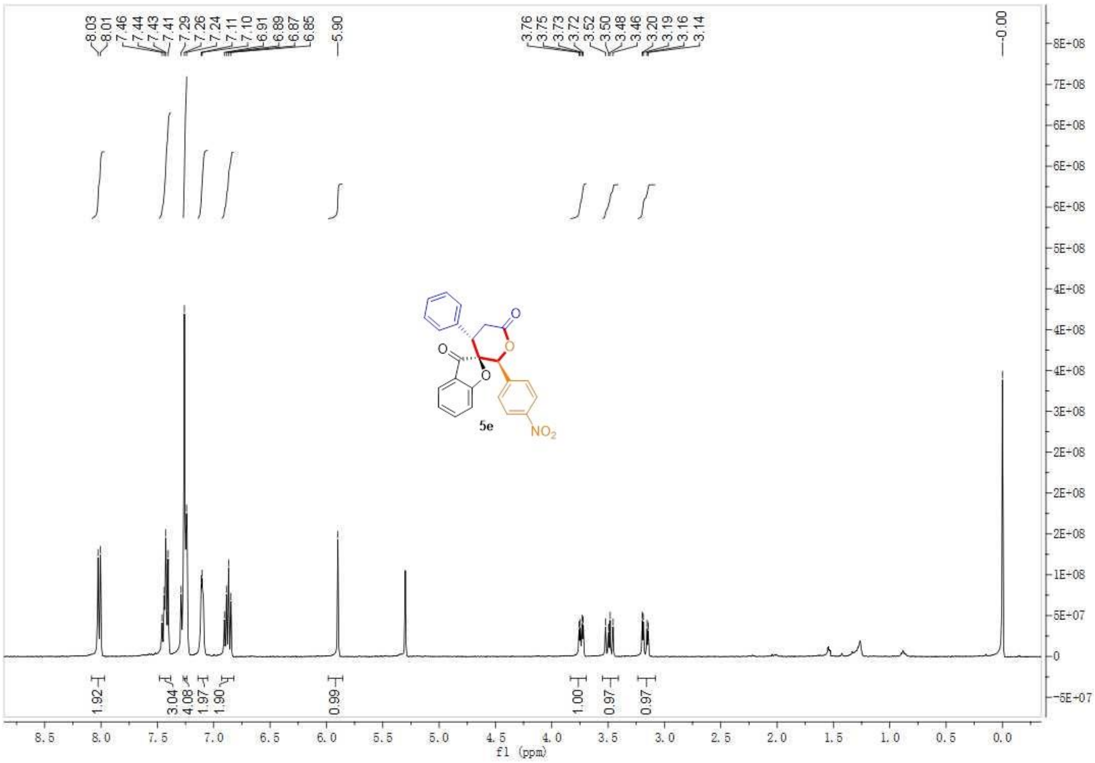
8.03
8.01
7.46
7.44
7.43
7.41
7.29
7.26
7.24
7.11
7.10
6.91
6.89
6.87
6.85
5.90
3.76
3.75
3.73
3.72
3.52
3.50
3.48
3.46
3.20
3.19
3.16
3.14
0.00
5e
NO2
1.92
3.04
4.08
1.97
1.90
0.99
1.00
0.97
0.97
f1 (ppm)

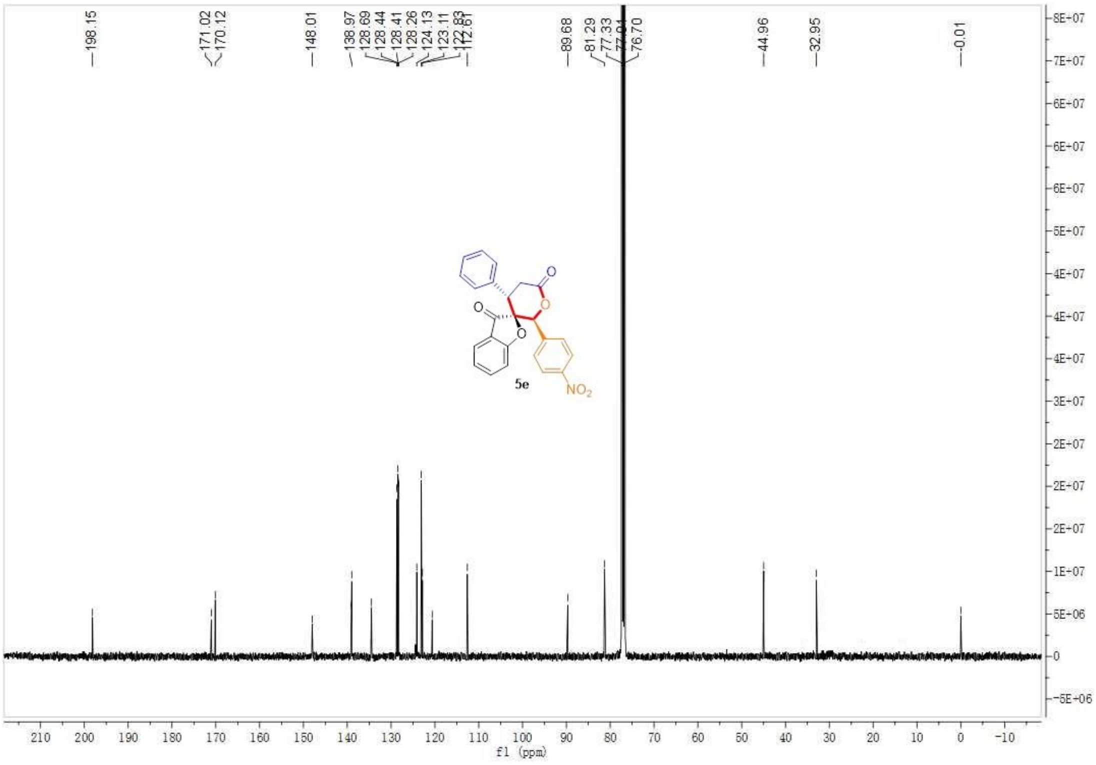
198.15
171.02
170.12
148.01
138.97
128.69
128.44
128.41
128.26
124.13
123.11
122.83
112.61
89.68
81.29
77.33
77.01
76.70
44.96
32.95
-0.01
5e
NO2
f1 (ppm)

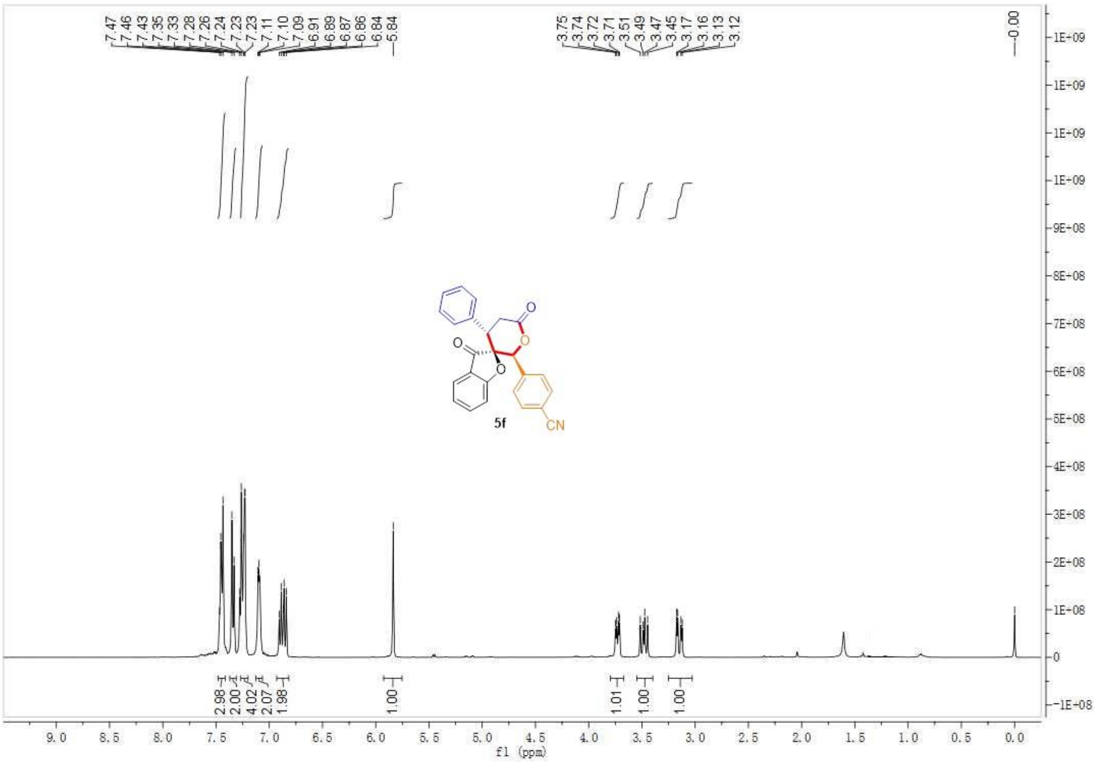
7.47
7.46
7.43
7.35
7.33
7.28
7.26
7.24
7.23
7.23
7.11
7.10
7.09
6.91
6.89
6.87
6.86
6.84
5.84
3.75
3.74
3.72
3.71
3.51
3.49
3.47
3.45
3.17
3.16
3.13
3.12
0.00
2.98
2.00
4.02
2.07
1.98
1.00
1.01
1.00
1.00
5f
CN
f1 (ppm)

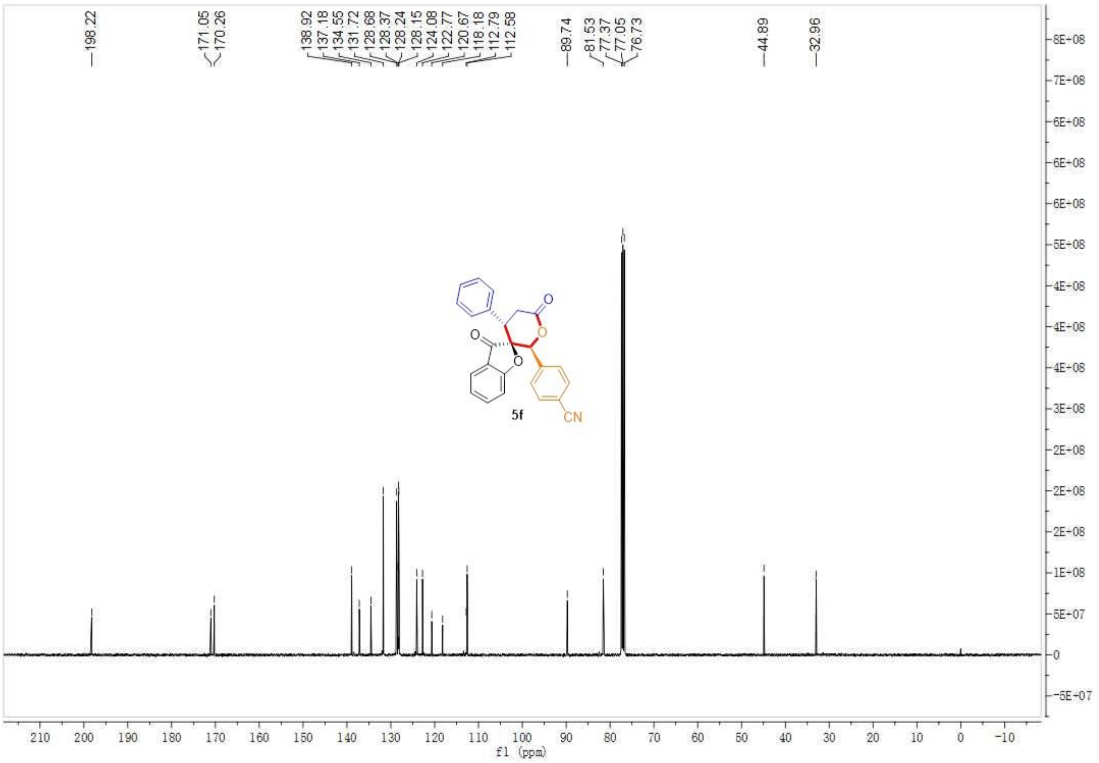
198.22
171.05
170.26
138.92
137.18
134.55
131.72
128.68
128.37
128.24
128.15
124.08
122.77
120.67
118.18
112.79
112.58
89.74
81.53
77.37
77.05
76.73
44.89
32.96
5f
CN
f1 (ppm)

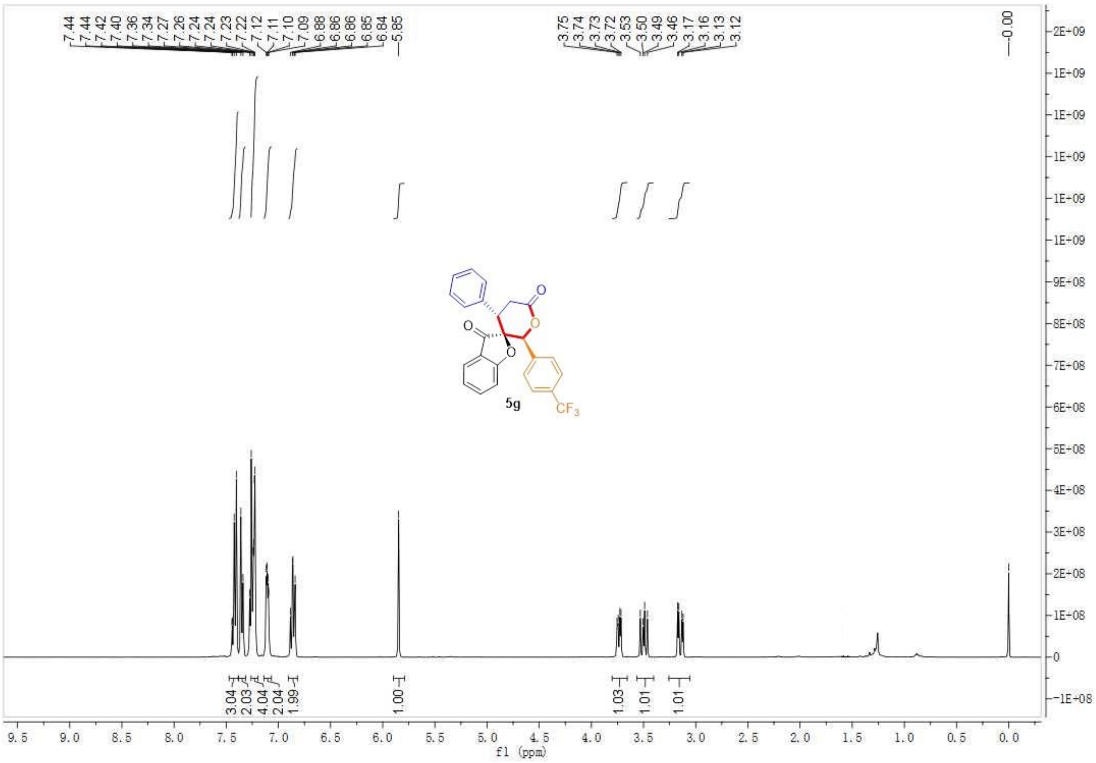

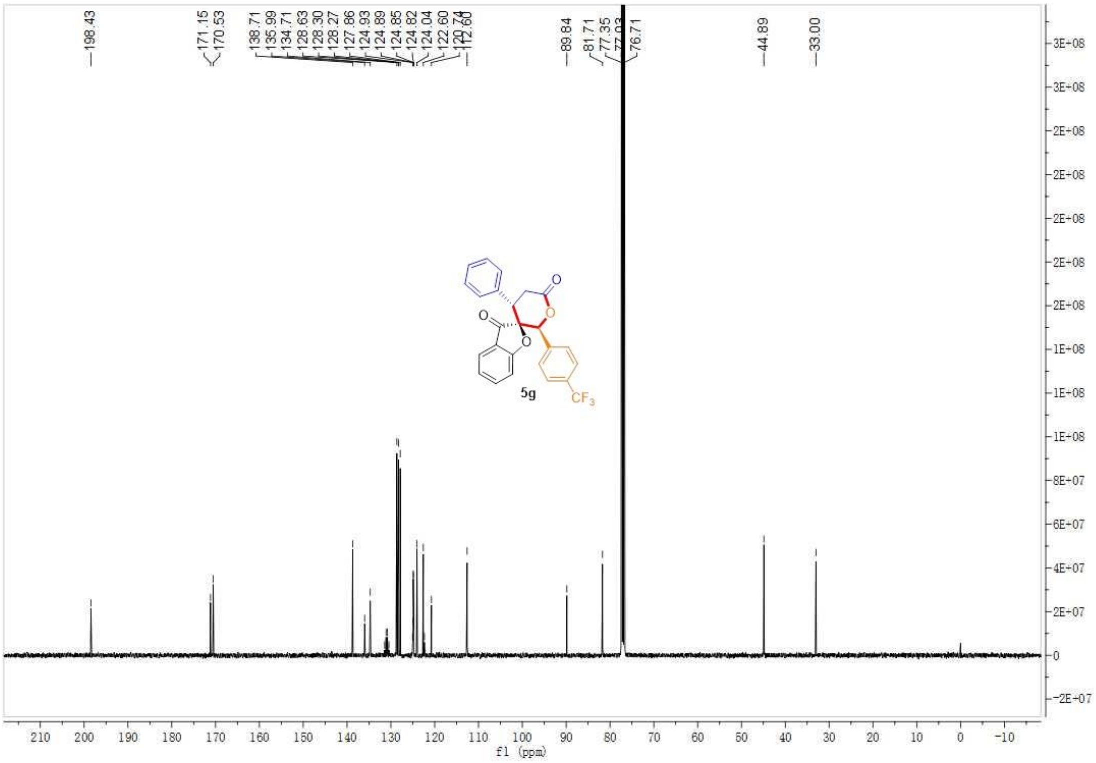

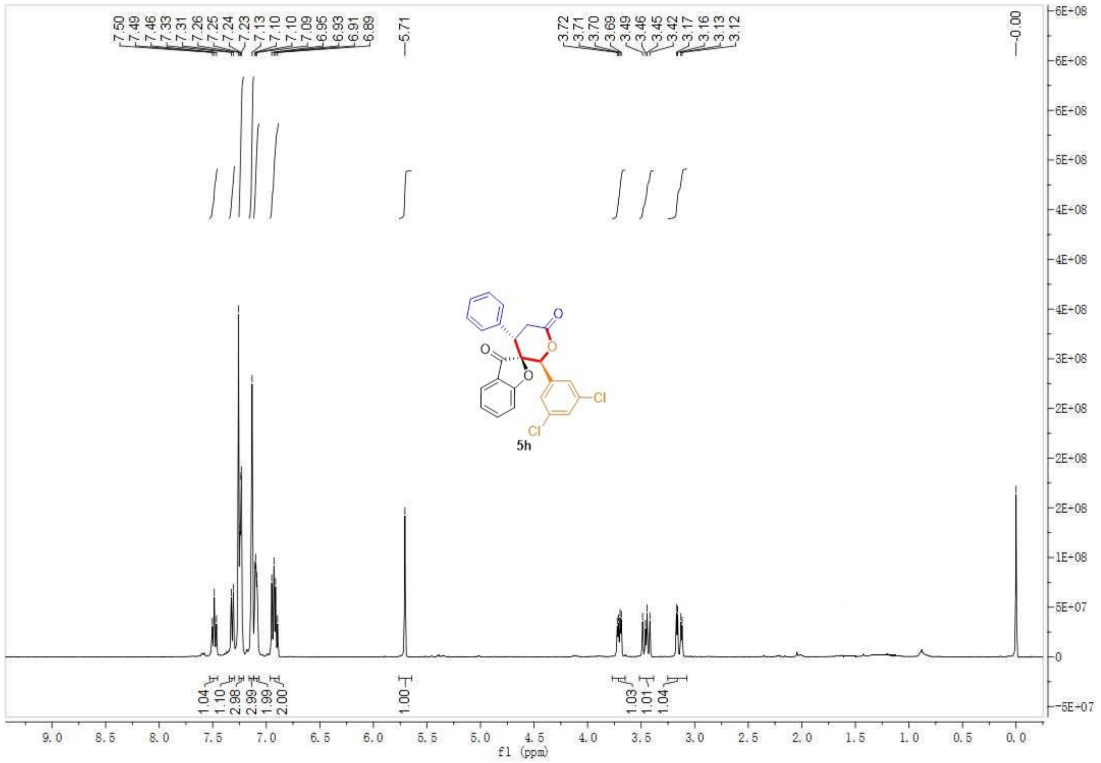
7.50 7.49 7.46 7.33 7.31 7.26 7.25 7.24 7.23 7.13 7.10 7.10 7.09 6.95 6.93 6.91 6.89
5.71
3.72 3.71 3.70 3.69 3.49 3.46 3.45 3.42 3.17 3.16 3.13 3.12
0.00
5h
1.04 1.10 2.98 2.99 1.99 2.00
1.00
1.03 1.01 1.04
f1 (ppm)

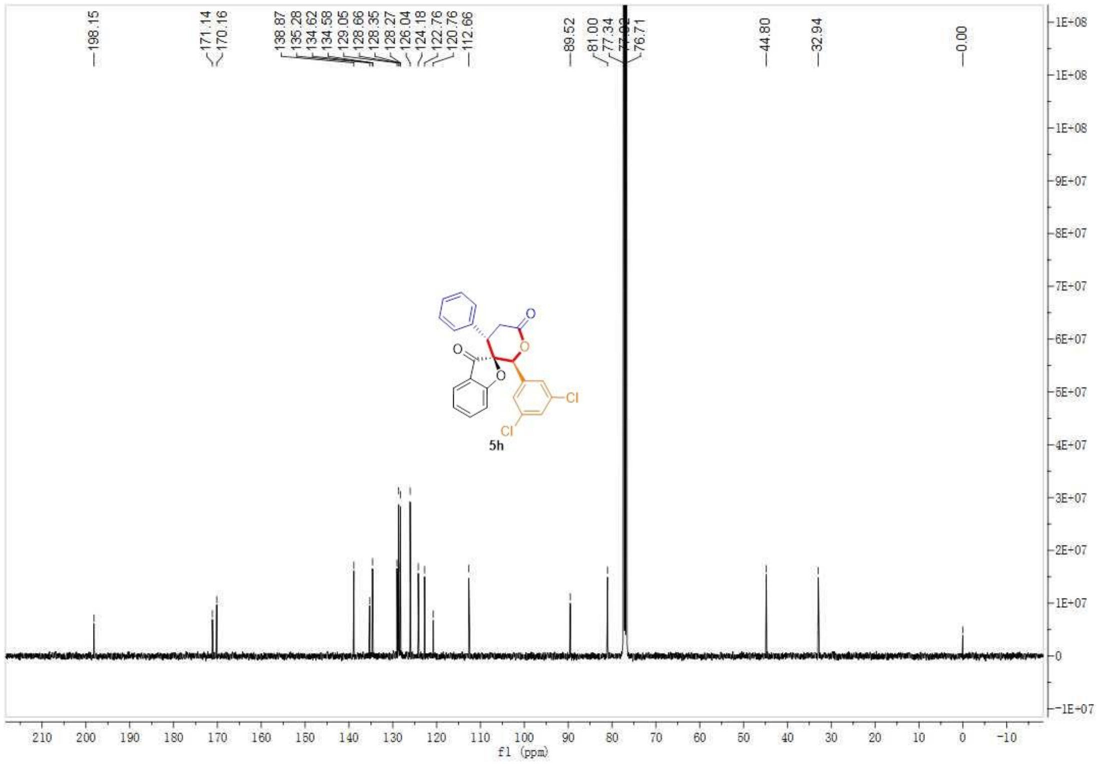
198.15
171.14 170.16
138.87 135.28 134.62 134.58 129.05 128.66 128.35 128.27 126.04 124.18 122.76 120.76 112.66
89.52 81.00 77.34 76.71
44.80
32.94
0.00
5h
f1 (ppm)

4 The data of crystal structure 3a

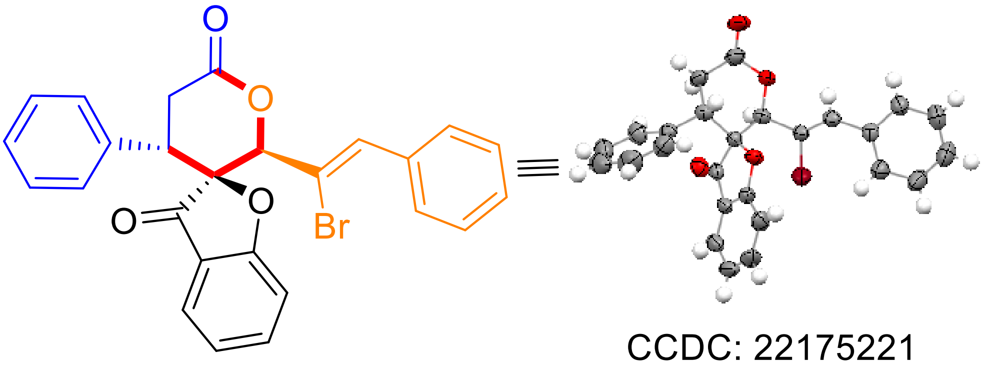

3a

CCDC: 22175221

Datablock: a

Bond precision: C-C = 0.0053 A Wavelength=0.71073

Cell:	a=9.128(3)	b=9.453(3)	c=12.581(4)
	alpha=90	beta=91.044(5)	gamma=90
Temperature:	296 K		

	Calculated	Reported
Volume	1085.4(6)	1085.4(5)
Space group	P 21	P2(1)
Hall group	P 2yb	?
Moiety formula	C26 H19 Br O4	?
Sum formula	C26 H19 Br O4	C26 H19 Br O4
Mr	475.31	475.32
Dx,g cm-3	1.454	1.454
Z	2	2
Mu (mm-1)	1.924	1.924
F000	484.0	484.0
F000'	483.64	
h,k,lmax	10,11,14	10,11,14
Nref	3823[2038]	3394
Tmin,Tmax	0.612,0.668	0.635,0.688
Tmin'	0.600	

Correction method= # Reported T Limits: Tmin=0.635 Tmax=0.688
AbsCorr = MULTI-SCAN

Data completeness= 1.67/0.89 Theta(max)= 25.000

R(reflections)= 0.0303(2948) wR2(reflections)= 0.0578(3394)

S = 1.072 Npar= 280
